# A national experiment reveals where a growth mindset improves achievement

**DOI:** 10.1038/s41586-019-1466-y

**Published:** 2019-08-07

**Authors:** David S. Yeager, Paul Hanselman, Gregory M. Walton, Jared S. Murray, Robert Crosnoe, Chandra Muller, Elizabeth Tipton, Barbara Schneider, Chris S. Hulleman, Cintia P. Hinojosa, David Paunesku, Carissa Romero, Kate Flint, Alice Roberts, Jill Trott, Ronaldo Iachan, Jenny Buontempo, Sophia Man Yang, Carlos M. Carvalho, P. Richard Hahn, Maithreyi Gopalan, Pratik Mhatre, Ronald Ferguson, Angela L. Duckworth, Carol S. Dweck

**Affiliations:** 1 University of Texas at Austin, Austin, TX USA; 2 University of California, Irvine, Irvine, CA USA; 3 Stanford University, Stanford, CA USA; 4 Northwestern University, Evanston, IL USA; 5 Michigan State University, East Lansing, MI USA; 6 University of Virginia, Charlottesville, VA USA; 7 University of Chicago, Chicago, IL USA; 8 Project for Education Research that Scales, San Francisco, CA USA; 9 Paradigm Strategy Inc., San Francisco, CA USA; 10 ICF, Fairfax, VA USA; 11 Arizona State University, Tempe, AZ USA; 12 The Pennsylvania State University, University Park, PA USA; 13 Harvard University, Cambridge, MA USA; 14 University of Pennsylvania, Philadelphia, PA USA

**Keywords:** Human behaviour, Risk factors, Human behaviour

## Abstract

A global priority for the behavioural sciences is to develop cost-effective, scalable interventions that could improve the academic outcomes of adolescents at a population level, but no such interventions have so far been evaluated in a population-generalizable sample. Here we show that a short (less than one hour), online growth mindset intervention—which teaches that intellectual abilities can be developed—improved grades among lower-achieving students and increased overall enrolment to advanced mathematics courses in a nationally representative sample of students in secondary education in the United States. Notably, the study identified school contexts that sustained the effects of the growth mindset intervention: the intervention changed grades when peer norms aligned with the messages of the intervention. Confidence in the conclusions of this study comes from independent data collection and processing, pre-registration of analyses, and corroboration of results by a blinded Bayesian analysis.

## Abstract (web summary)

A US national experiment showed that a short, online, self-administered growth mindset intervention can increase adolescents’ grades and advanced course-taking, and identified the types of school that were poised to benefit the most.

## Main

About 20% of students in the United States will not finish high school on time^1^. These students are at a high risk of poverty, poor health and early mortality in the current global economy^2–4^. Indeed, a *Lancet* commission concluded that improving secondary education outcomes for adolescents “presents the single best investment for health and wellbeing”^5^.

The transition to secondary school represents an important period of flexibility in the educational trajectories of adolescents^6^. In the United States, the grades of students tend to decrease during the transition to the ninth grade (age 14–15 years, UK year 10), and often do not recover^7^. When such students underperform in or opt out of rigorous coursework, they are far less likely to leave secondary school prepared for college or university or for advanced courses in college or university^8,9^. In this way, early problems in the transition to secondary school can compound over time into large differences in human capital in adulthood.

One way to improve academic success across the transition to secondary school is through social–psychological interventions, which change how adolescents think or feel about themselves and their schoolwork and thereby encourage students to take advantage of learning opportunities in school^10,11^. The specific intervention evaluated here—a growth mindset of intelligence intervention—addresses the beliefs of adolescents about the nature of intelligence, leading students to see intellectual abilities not as fixed but as capable of growth in response to dedicated effort, trying new strategies and seeking help when appropriate^12–16^. This can be especially important in a society that conveys a fixed mindset (a view that intelligence is fixed), which can imply that feeling challenged and having to put in effort means that one is not naturally talented and is unlikely to succeed^12^.

The growth mindset intervention communicates a memorable metaphor: that the brain is like a muscle that grows stronger and smarter when it undergoes rigorous learning experiences^14^. Adolescents hear the metaphor in the context of the neuroscience of learning, they reflect on ways to strengthen their brains through schoolwork, and they internalize the message by teaching it to a future first-year ninth grade student who is struggling at the start of the year. The intervention can lead to sustained academic improvement through self-reinforcing cycles of motivation and learning-oriented behaviour. For example, a growth mindset can motivate students to take on more rigorous learning experiences and to persist when encountering difficulties. Their behaviour may then be reinforced by the school context, such as more positive and learning-oriented responses from peers or instructors^10,17^.

Initial intervention studies with adolescents taught a growth mindset in multi-session (for example, eight classroom sessions^15^), interactive workshops delivered by highly trained adults; however, these were not readily scalable. Subsequent growth mindset interventions were briefer and self-administered online, although lower effect sizes were, of course, expected. Nonetheless, previous randomized evaluations, including a pre-registered replication, found that online growth mindset interventions improved grades for the targeted group of students in secondary education who previously showed lower achievement^13,16,18^. These findings are important because previously low-achieving students are the group that shows the steepest decline in grades during the transition to secondary school^19^, and these findings are consistent with theory because a growth mindset should be most beneficial for students confronting challenges^20^.

**Fig. 1:**
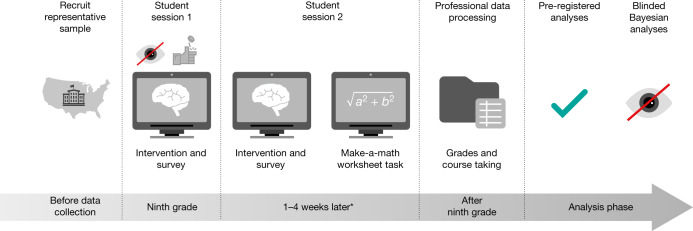
Design of the National Study of Learning Mindsets. Between August and November 2015, 82% of schools delivered the intervention; the remaining 18% delivered the intervention in January or February of 2016. Asterisk indicates that the median number of days between sessions 1 and 2 among schools implementing the intervention in the autumn was 21 days; for spring-implementing schools it was 27 days. The coin-tossing symbol indicates that random assignment was made during session 1. The tick symbol indicates that a comprehensive analysis plan was pre-registered at https://osf.io/tn6g4. The blind-eye symbol indicates that, first, teachers and researchers were kept blinded to students’ random assignment to condition, and, second, the Bayesian, machine-learning robustness tests were conducted by analysts who at the time were blinded to study hypotheses and to the identities of the variables.

Here we report the results of the National Study of Learning Mindsets, which examined the effects of a short, online growth mindset intervention in a nationally representative sample of high schools in the United States (Fig. 1). With this unique dataset we tested the hypotheses that the intervention would improve grades among lower-achieving students and overall uptake of advanced courses in this national sample.

## A focus on heterogeneity

The study was also designed with the purpose of understanding for whom and under what conditions the growth mindset intervention improves grades. That is, it examined potential sources of cross-site treatment effect heterogeneity. One reason why understanding heterogeneity of effects is important is because most interventions that are effective in initial efficacy trials go on to show weaker or no effects when they are scaled up in effectiveness trials that deliver treatments under everyday conditions to more heterogeneous samples^21–23^. Without clear evidence about why average effect sizes differ in later-conducted studies—evidence that could be acquired from a systematic investigation of effect heterogeneity—researchers may prematurely discard interventions that yield low average effects but could provide meaningful and replicable benefits at scale for targeted groups^21,23^.

Further, analyses of treatment effect heterogeneity can reveal critical evidence about contextual mechanisms that sustain intervention effects. If school contexts differ in the availability of the resources or experiences needed to sustain the offered belief change and enhanced motivation following an intervention, then the effects of the intervention should differ across these school contexts as well^10,11^.

Sociological theory highlights two broad dimensions of school contexts that might sustain or impede belief change and enhanced motivation among students treated by a growth mindset intervention^6^. First, schools with the least ‘formal’ resources, such as high-quality curricula and instruction, may not offer the learning opportunities for students to be able to capitalize on the intervention, while those with the most resources may not need the intervention. Second, some schools may not have the ‘informal’ resources needed to sustain the intervention effect, such as peer norms that support students when they take on challenges and persist in the face of intellectual difficulty. We hypothesized that both of these dimensions would significantly moderate growth mindset intervention effects.

Historically, the scientific methods used to answer questions about the heterogeneity of intervention effects have been underdeveloped and underused^21,24,25^. Common problems in the literature are: (1) imprecise site-level impact estimates (because of cluster-level random assignment); (2) inconsistent fidelity to intervention protocols across sites (which can obscure the workings of the cross-site moderators of interest); (3) non-representative sampling of sites (which causes site selection bias^22,26^); and (4) multiple post hoc tests for the sources of treatment effect size heterogeneity (which increases the probability of false discoveries^24^).

We overcame all of these problems in a single study. We randomized students to condition within schools and consistently had high fidelity of implementation across sites (see Supplementary Information section 5). We addressed site selection bias by contracting a professional research company, which recruited a sample of schools that generalized to the entire population of ninth-grade students attending regular US public schools^27^ (that is, schools that run on government funds; see Supplementary Information section 3). Next, the study used analysis methods that avoided false conclusions about subgroup effects, by generating a limited number of moderation hypotheses (two), pre-registering a limited number of statistical tests and conducting a blinded Bayesian analysis that can provide rigorous confirmation of the results (Fig. 1).

## Expected effect sizes

In this kind of study, it is important to ask what size of effect would be meaningful. As a leading educational economist concluded, “in real-world settings, a fifth of a standard deviation [0.20 s.d.] is a large effect”^28^. This statement is justified by the ‘best evidence synthesis’ movement^29^, which recommends the use of empirical benchmarks, not from laboratory studies, but from the highest-quality field research on factors affecting objective educational outcomes^30,31^. A standardized mean difference effect size of 0.20 s.d. is considered ‘large’ because it is: (1) roughly how much improvement results from a year of classroom learning for ninth-grade students, as shown by standardized tests^30^; (2) at the high end of estimates for the effect of having a very high-quality teacher (versus an average teacher) for one year^32^; and (3) at the uppermost end of empirical distributions of real-world effect sizes from diverse randomized trials that target adolescents^31^. Notably, the highly-cited ‘nudges’ studied by behavioural economists and others, when aimed at influencing real-world outcomes that unfold over time (such as college enrolment or energy conservation^33^) rather than one-time choices, rarely, if ever, exceed 0.20 s.d. and typically have much smaller effect sizes.

Returning to educational benchmarks, 0.20 s.d. and 0.23 s.d. were the two largest effects observed in a recent cohort analysis of the results of all of the pre-registered, randomized trials that evaluated promising interventions for secondary schools funded as part of the US federal government’s i3 initiative^34^ (the median effect for these promising interventions was 0.03 s.d.; see Supplementary Information section 11). The interventions in the i3 initiative typically targeted lower-achieving students or schools, involved training teachers or changing curricula, consumed considerable classroom time, and cost several thousand US dollars per student. Moreover, they were all conducted in non-representative samples of convenience that can overestimate effects. Therefore, it would be noteworthy if a short, low-cost, scalable growth mindset intervention, conducted in a nationally representative sample, could achieve a meaningful proportion of the largest effects seen for past traditional interventions, within the targeted, pre-registered group of lower-achieving students.

## Defining the primary outcome and student subgroup

**Extended Data Fig. 1:**
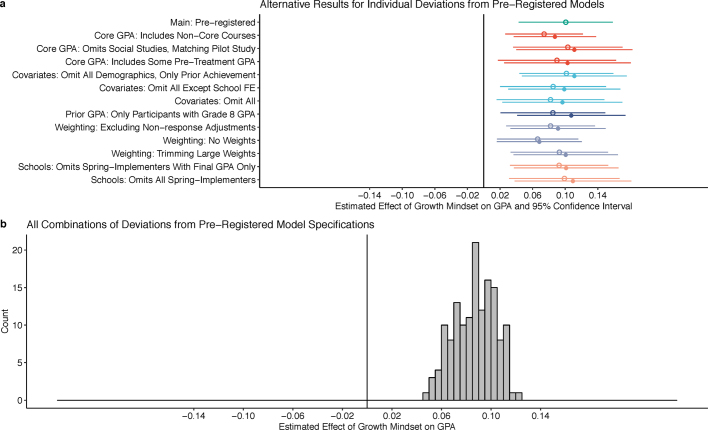
The finding that the growth mindset effect on GPA is positive among lower-achieving students is robust to deviations from the pre-registered statistical model. **a**, **b**, Each estimate represents an unstandardized treatment effect on GPA (on a 0 to 4.3 scale) estimated in separate fixed-effects regression models with school as a fixed effect. Most of the alternative specifications were known to produce less-valid tests of the hypothesis, but some of them required fewer subjective judgments and so it was informative to show that the main conclusion of a positive treatment effect was supported even with a suboptimal model specification. Examples include revising the core GPA outcome to include non-core classes such as speech, debate or electives (because this does not involve coding of core classes; see ‘Includes Non-Core Courses’), or revising the post-treatment marking period to include pre-treatment data in cases in which schools implemented the intervention in the Spring (because this does not involve coding pre- and post-treatment making periods; see ‘Includes Some Pre-Treatment GPA’). **a**, The effects of changing just one or two model specifications at a time while leaving the rest of the pre-registered model specifications the same. Open circles represent the pre-registered definition of lower-achieving students (below the school-specific median), and filled dots represent the alternative definition of lower-achieving students (below the school-specific median and below a 3.0 GPA out of 4.3). **b**, A histogram of all possible combinations of the alternative model specifications that shows that effects are uniformly positive. Note that the treatment effect estimates on the far left of **b** are from clearly less-valid models; for example, they insufficiently control for prior achievement, they drop participants with missing data, they do not use survey weights (so results are not representative and therefore do not answer our research questions). Panels **a** and **b** both show that even exercising all of these degrees of freedom in a way that could obscure true treatment effects still yields positive point estimates. Further explanations of why the alternatives were not selected for the pre-registration are included in Supplementary Information section 7.3. Source data

The primary outcome was the post-intervention grade point average (GPA) in core ninth-grade classes (mathematics, science, English or language arts, and social studies), obtained from administrative data sources of the schools (as described in the pre-analysis plan found in the Supplementary Information section 13 and at https://osf.io^35^). Following the pre-registered analysis plan, we report results for the targeted group of *n* = 6,320 students who were lower-achieving relative to peers in the same school. This group is typically targeted by comprehensive programmes evaluated in randomized trials in education, as there is an urgent need to improve their educational trajectories. The justification for predicting effects in the lower-achieving group is that (1) this group benefitted in previous growth mindset trials; (2) lower-achieving students may be undergoing more academic difficulties and therefore may benefit more from a growth mindset that alters the interpretation of these difficulties; and (3) students who already have a high GPA may have less room to improve their GPAs. We defined students as relatively lower-achieving if they were earning GPAs at or below the school-specific median in the term before random assignment or, if they were missing prior GPA data, if they were below the school-specific median on academic variables used to impute prior GPA (as described in the analysis plan). Supplementary analyses for the sample overall can be found in Extended Data Table 1, and robustness analyses for the definition of lower-achieving students are included in Extended Data Fig. 1 (Supplementary Information section 7).

## Average effects on mindset

Among lower-achieving adolescents, the growth mindset intervention reduced the prevalence of fixed mindset beliefs relative to the control condition, reported at the end of the second treatment session, unstandardized *B* = −0.38 (95% confidence interval = −0.31, −0.46), standard error of the regression coefficient (s.e.) = 0.04, *n* = 5,650 students, *k* = 65 schools, *t* = −10.14, *P* < 0.001, standardized mean difference effect size of 0.33.

## Average effects on core course GPAs

In line with our first major prediction, lower-achieving adolescents earned higher GPAs in core classes at the end of the ninth grade when assigned to the growth mindset intervention, *B =* 0.10 grade points (95% confidence interval = 0.04, 0.16), s.e. = 0.03, *n* = 6,320, *k* = 65, *t* = 3.51, *P* = 0.001, standardized mean difference effect size of 0.11, relative to comparable students in the control condition. This conclusion is robust to alternative model specifications that deviate from the pre-registered model (Extended Data Fig. 1).

To map the growth mindset intervention effect onto a policy-relevant indicator of high school success, we analysed poor performance rates, defined as the percentage of adolescents who earned a GPA below 2.0 on a four-point scale (that is, a ‘D’ or an ‘F’; as described in the pre-analysis plan). Poor performance rates are relevant because recent changes in US federal laws (the Every Student Succeeds Act^36^), have led many states to adopt reductions in the poor performance rates in the ninth grade as a key metric for school accountability. More than three million ninth-grade students attend regular US public schools each year, and half are lower-achieving according to our definition. The model estimates that 5.3% (95% confidence interval = −1.7, −9.0), s.e. = 1.8, *t* = 2.95, *P* = 0.005 of 1.5 million students in the United States per year would be prevented from being ‘off track’ for graduation by the brief and low-cost growth mindset intervention, representing a reduction from 46% to 41%, which is a relative risk reduction of 11% (that is, 0.05/0.46).

## Average effects on mathematics and science GPAs

A secondary analysis focused on the outcome of GPAs in only mathematics and science (as described in the analysis plan). Mathematics and science are relevant because a popular belief in the United States links mathematics and science learning to ‘raw’ or ‘innate’ abilities^37^—a view that the growth mindset intervention seeks to correct. In addition, success in mathematics and science strongly predicts long-term economic welfare and well-being^38^. Analyses of outcomes for mathematics and science supported the same conclusions (*B* = 0.10 for mathematics and science GPAs compared to *B* = 0.10 for core GPAs; Extended Data Tables 1–3).

## Quantifying heterogeneity

**Extended Data Fig. 2:**
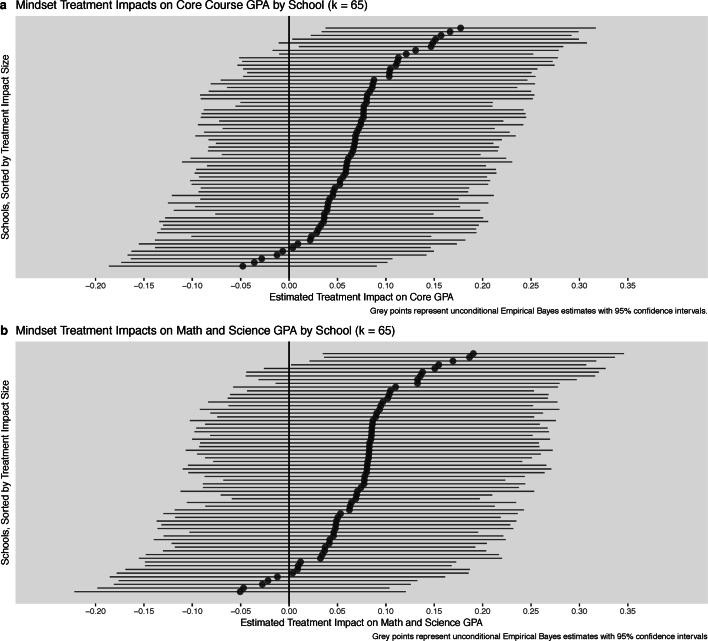
The growth mindset intervention effect in a given school is almost always positive, although there is significant heterogeneity across schools. **a**, **b**, Mindset treatment effects on for core course GPAs (**a**) and mathematics/science GPAs (**b**). Estimates were generated using the pre-registered linear mixed-effects model (see Supplementary Information section 7, RQ3). Note that the treatment effect at any individual school is likely to have a very wide confidence interval even when there is a true positive effect, owing to small sample sizes for each school on its own. Therefore, as with any multi-site trial, effects of individual schools are not expected to be significantly different from zero even though the average treatment effect is significantly different from zero. The plotted treatment effects were estimated in an unconditional model with no cross-level interactions (that is, without consideration of the potential moderators) and so the points are shrunken towards the sample mean. Thus, these plotted estimates do not correspond to the estimated CATEs reported in the paper or in Extended Data Table 3. Source data

The intervention was expected to homogeneously change the mindsets of students across schools—as this would indicate high fidelity of implementation—however, it was expected to heterogeneously change lower-achieving students’ GPAs, as this would indicate potential school differences in the contextual mechanisms that sustain an initial treatment effect. As predicted, a mixed-effects model found no significant variability in the treatment effect on self-reported mindsets across schools (unstandardized $\hat{\tau }=0.08$, *Q*_64_ = 57.2, *P* = 0.714), whereas significant variability was found in the effect on GPAs among lower-achieving students across schools (unstandardized $\hat{\tau }=0.09$, *Q*_64_ = 85.5, *P* = 0.038)^39^ (Extended Data Fig. 2).

## Moderation by school achievement level

First, we tested competing hypotheses about whether the formal resources of the school explained the heterogeneity of effects. Before analysing the data, we expected that in schools that are unable to provide high-quality learning opportunities (the lowest-achieving schools), treated students might not sustain a desire to learn. But we also expected that other schools (the highest-achieving schools) might have such ample resources to prevent failure such that a growth mindset intervention would not add much.

The heterogeneity analyses found support for the latter expectation, but not the former. Treatment effects on ninth-grade GPAs among lower-achieving students were smaller in schools with higher achievement levels, intervention × school achievement level (continuous) interaction, unstandardized *B* = −0.07 (95% confidence interval = 0.02, 0.13), s.e. = 0.03, *z* = −2.76, *n* = 6,320, *k* = 65, *P* = 0.006, standardized *β* = −0.25. In follow-up analyses with categorical indicators for school achievement, medium-achieving schools (middle 50%) showed larger effects than higher-achieving schools (top 25%). Low-achieving schools (bottom 25%) did not significantly differ from medium-achieving schools (Extended Data Table 2); however, this non-significant difference should be interpreted cautiously, owing to wide confidence intervals for the subgroup of lowest-achieving schools.

## Moderation by peer norms

Second, we examined whether students might be discouraged from acting on their enhanced growth mindset when they attend schools in which peer norms were unsupportive of challenge-seeking, whereas peer norms that support challenge-seeking might function to sustain the effects of the intervention over time. We measured peer norms by administering a behavioural challenge-seeking task (the ‘make-a-math-worksheet’ task) at the end of the second intervention session (Fig. 1) and aggregating the values of the control group to the school level.

The pre-registered mixed-effects model yielded a positive and significant intervention × behavioural challenge-seeking norms interaction for GPA among the targeted group of lower-achieving adolescents, such that the intervention produced a greater difference in end-of-year GPAs relative to the control group when the behavioural norm that surrounded students was supportive of the growth mindset belief system, *B* = 0.11 (95% confidence interval = 0.01, 0.21), s.e. = 0.05, *z* = 2.18, *n* = 6,320, *k* = 65, *P* = 0.029, *β* = 0.23. The same conclusion was supported in a secondary analysis of only mathematics and science GPAs (Extended Data Table 2).

## Subgroup effect sizes

**Fig. 2:**
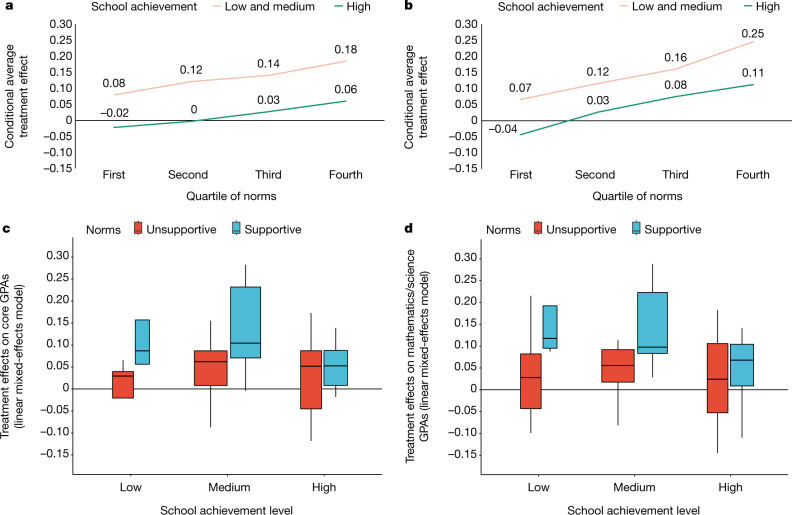
The growth mindset intervention effects on grade point averages were larger in schools with peer norms that were supportive of the treatment message. **a**, **c**, Treatment effects on core course grade point averages (GPAs). **b**, **d**, Treatment effects on GPAs of only mathematics and science. **a**, **b**, The CATEs represent the estimated subgroup treatment effects from the pre-registered linear mixed-effects model, with survey weights, when fixing the racial/ethnic composition of the schools to the population median to remove any potential confounding effect of that variable on moderation hypothesis tests. Achievement levels: low, 25th percentile or lower; middle, 25th–75th percentile; high, 75th percentile or higher, which follows the categories set in the sampling plan and in the pre-registration. Norms indicate the behavioural challenge-seeking norms, as measured by the responses of the control group to the make-a-math-worksheet task after session 2. **c**, **d**, Box plots represent unconditional treatment effects (one for each school) estimated in the pre-registered linear mixed-effects regression model with no school-level moderators, as specified for research question 3 in the pre-analysis plan and described in the Supplementary Information section 7.4. The distribution of the school-level treatment effects was re-scaled to the cross-site standard deviation, in accordance with standard practice. Dark lines correspond to the median school in a subgroup and the boxes correspond to the middle 75% of the distribution (the interquartile range). Supportive schools are defined as above the population median (third and fourth quartiles); unsupportive schools are defined as those below the population median (first and second quartiles). *n* = 6,320 students in *k* = 65 schools. Source data

Putting together the two pre-registered moderators (school achievement level and school norms), the conditional average treatment effect (CATEs) on core GPAs within low- and medium-achieving schools (combined) was 0.14 grade points when the school was in the third quartile of behavioural norms and 0.18 grade points when the school was in the fourth and highest quartile of behavioural norms, as shown in Fig. 2. For mathematics and science grades, the CATEs ranged from 0.16 to 0.25 grade points in the same subgroups of low- and medium-achieving schools with more supportive behavioural norms (for results separating low- and medium-achieving schools, see Fig. 2c, d and Extended Data Table 3). We also found that even the high-achieving schools showed meaningful treatment effects among their lower achievers on mathematics and science GPAs when they had norms that supported challenge seeking—0.08 and 0.11 grade points for the third and fourth quartiles of school norms, respectively, in the high-achieving schools (*P* = 0.002; Extended Data Table 3).

## Bayesian robustness analysis

A team of statisticians, at the time blind to study hypotheses, re-analysed the dataset using a conservative Bayesian machine-learning algorithm, called Bayesian causal forest (BCF). BCF has been shown by both its creators and other leading statisticians in open head-to-head competitions to be the most effective of the state-of-the-art methods for identifying systematic sources of treatment effect heterogeneity, while avoiding false positives^40,41^.

**Extended Data Fig. 3:**
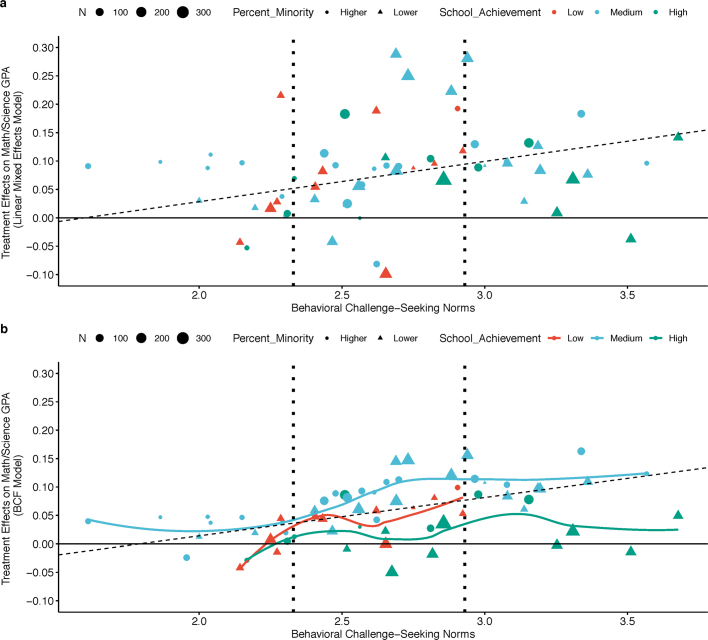
A BCF analysis reproduces the same pattern of moderation by norms as the pre-registered linear mixed-effects model The BCF analysis uses a nonparametric Bayesian model designed to shrink effect sizes to see if any effect can update a relatively strong prior centered on null effects and biased toward low degrees of treatment effect moderation. **a**, **b**, Data points correspond to school-level treatment effects estimated by the pre-registered linear mixed-effects model (**a**) or the BCF model (**b**). Treatment effects refers to the difference between the treatment and control groups in terms of mathematics/science GPAs at the end of ninth grade in a school, adjusting for pre-random-assignment covariates and including survey weights. The models included three school-level moderators of the student-level randomized treatment: the achievement level (categorical, dummies for low and high, medium group as the reference category in the linear model), the behavioural growth mindset norms (continuous) and the percentage of racial or ethnic minority students (continuous) of the schools. School-level treatment effects include the fitted values plus the model-estimated, school-specific random effect. Challenge-seeking behavioural norm refers to the average number of challenging mathematics problems (out of 8) chosen by students in the control group in a given school. *N*, the number of lower-achieving students in a school. Percent minority, the percentage of students who identify as black, African-American, Hispanic, Latino or indigenous American, split at the school-level population median (26% of the student body of the school). The dashed lines represent the estimated intercept and slope for the linear trend of the estimated treatment effects in norms. **b**, The coloured lines represent LOESS smoothing curves for the trend in  norms of the estimated treatment effects, fitted to the estimated school-level treatment effects within achievement groups and weighted by school sample size. The area between thevertical lines is the interquartile range (IQR) of norms, where neither model is extrapolating. The two models agree broadly about average effects, particularly within the IQR of norms, while BCF estimated somewhat lower degrees of heterogeneity and extrapolates in a fundamentally different fashion at the extremes of norms (since it is a nonlinear model).Recall BCF is designed to shrink toward an overall effect size of zero, and to shrink CATEs of similar schools towards one another, in order to avoid over-fitting the data. Unlike the preregistered linear models, BCF was specified with no prior hypotheses about the functional form of moderation (nonlinearities and/or interactions between multiple moderators and treatment) so this shrinkage is necessary to obtain stable estimates of treatment effects. However, it does lead to smaller estimates of effect sizes and a lower estimated degree of moderation relative to the preregistered linear mixed effects model. Source data

The BCF analysis assigned a near-certain posterior probability that the population-average treatment effect (PATE) among lower-achieving students was positive and greater than zero, *P*_PATE > 0_ ≥ 0.999, providing strong evidence of positive average treatment effects. BCF also found stronger CATEs in schools with positive challenge-seeking norms, and weaker effects in the highest-achieving schools (Extended Data Fig. 3 and Supplementary Information section 8), providing strong correspondence with the primary analyses.

## Advanced mathematics course enrolment in tenth grade

The intervention showed weaker benefits on ninth-grade GPAs in high-achieving schools. However, students in these schools may benefit in other ways. An analysis of enrolment in rigorous mathematics courses in the year after the intervention examined this possibility. The enrolment data were gathered with these analyses in mind but since the analyses were not pre-registered, they are exploratory.

Course enrolment decisions are potentially relevant to all students, both lower- and higher-achieving, so we explored them in the full cohort. We found that the growth mindset intervention increased the likelihood of students taking advanced mathematics (algebra II or higher) in tenth grade by 3 percentage points (95% confidence interval = 0.01, 0.04), s.e. = 0.01, *n* = 6,690, *k* = 41, *t* = 3.18, *P* = 0.001, from a rate of 33% in the control condition to a rate of 36% in the intervention condition, corresponding to a 9% relative increase. Notably, we discovered a positive intervention × school achievement level (continuous) interaction, (*B* = 0.04 (95% confidence interval = 0.00, 0.08), s.e. = 0.02, *z* = 2.26, *P* = 0.024, the opposite of what we found for core course GPAs. Within the highest-achieving 25% of schools, the intervention increased the rate at which students took advanced mathematics in tenth grade by 4 percentage points (*t* = 2.37, *P* = 0.018). In the lower 75% of schools—where we found stronger effects on GPA—the increase in the rate at which students took advanced mathematics courses was smaller: 2 percentage points (*t* = 2.00, *P* = 0.045). Thus an exclusive focus on GPA would have obscured intervention benefits among students attending higher-achieving schools.

## Discussion

The National Study of Learning Mindsets showed that a low-cost treatment, delivered in less than an hour, attained a substantial proportion of the effects on grades of the most effective rigorously evaluated adolescent interventions of any cost or duration in the literature within the pre-registered group of lower-achieving students. Moreover, the intervention produced gains in the consequential outcome of advanced mathematics course-taking for students overall, which is meaningful because the rigor of mathematics courses taken in high school strongly predicts later educational attainment^8,9^, and educational attainment is one of the leading predictors of longevity and health^38,42^. The finding that the growth mindset intervention could redirect critical academic outcomes to such an extent—with no training of teachers; in an effectiveness trial conducted in a population-generalizable sample; with data collected by an independent research company using repeatable procedures; with data processed by a second independent research company; and while adhering to a comprehensive pre-registered analysis plan—is a major advance.

Furthermore, the evidence about the kinds of schools where the growth mindset treatment effect on grades was sustained, and where it was not, has important implications for future interventions. We might have expected that the intervention would compensate for unsupportive school norms, and that students who already had supportive peer norms would not need the intervention as much. Instead, it was when the peer norm supported the adoption of intellectual challenges that the intervention promoted sustained benefits in the form of higher grades.

Perhaps students in unsupportive peer climates risked paying a social price for taking on intellectual challenges in front of peers who thought it undesirable to do so. Sustained change may therefore require both a high-quality seed (an adaptive belief system conveyed by a compelling intervention) and conductive soil in which that seed can grow (a context congruent with the proffered belief system). A limitation of our moderation results, of course, is that we cannot draw causal conclusions about the effects of the school norm, as the norms were measured, not manipulated. It is encouraging that a Bayesian analysis, reported in the Supplementary Information section 8, yielded evidence consistent with a causal interpretation of the school norms variable. The present research therefore sets the stage for a new era of experimental research that seeks to enhance both students’ mindsets and the school environments that support student learning.

We emphasize that not all forms of growth mindset interventions can be expected to increase grades or advanced course-taking, even in the targeted subgroups^11,12^. New growth mindset interventions that go beyond the module and population tested here will need to be subjected to rigorous development and validation processes, as the current programme was^13^.

Finally, this study offers lessons for the science of adolescent behaviour change. Beliefs—and particularly beliefs that affect how students make sense of ongoing challenges—are important during high-stakes developmental turning points such as pubertal maturation^43,44^ or the transition to secondary school^6^. Indeed, new interventions in the future should address the interpretation of other challenges that adolescents experience, including social and interpersonal difficulties, to affect outcomes (such as depression) that thus far have proven difficult to address^43^. And the combined importance of belief change and school environments in our study underscores the need for interdisciplinary research to understand the numerous influences on adolescents’ developmental trajectories.

## Methods

### Ethics approval

Approval for this study was obtained from the Institutional Review Board at Stanford University (30387), ICF (FWA00000845), and the University of Texas at Austin (#2016-03-0042). In most schools this experiment was conducted as a programme evaluation carried out at the request of the participating school district^45^. When required by school districts, parents were informed of the programme evaluation in advance and given the opportunity to withdraw their children from the study. Informed student assent was obtained from all participants.

### Participants

Data came from the National Study of Learning Mindsets^45^, which is a stratified random sample of 65 regular public schools in the United States that included 12,490 ninth-grade adolescents who were individually randomized to condition. The number of schools invited to participate was determined by a power analysis to detect reasonable estimates of cross-site heterogeneity; as many of the invited schools as possible were recruited into the study. Grades were obtained from the schools of the students, and analyses focused on the lower-achieving subgroup of students (those below the within-school median). The sample reflected the diversity of young people in the United States: 11% self-reported being black/African-American, 4% Asian-American, 24% Latino/Latina, 43% white and 18% another race or ethnicity; 29% reported that their mother had a bachelor’s degree or higher. To prevent deductive disclosure for potentially-small subgroups of students, and consistent with best practices for other public-use datasets, the policies for the National Study of Learning Mindsets require analysts to round all sample sizes to the nearest 10, so this was done here.

### Data collection

To ensure that the study procedures were repeatable by third parties and therefore scalable, and to increase the independence of the results, two different professional research companies, who were not involved in developing the materials or study hypotheses, were contracted. One company (ICF) drew the sample, recruited schools, arranged for treatment delivery, supervised and implemented the data collection protocol, obtained administrative data, and cleaned and merged data. They did this work blind to the treatment conditions of the students. This company worked in concert with a technology vendor (PERTS), which delivered the intervention, executed random assignment, tracked student response rates, scheduled make-up sessions and kept all parties blind to condition assignment. A second professional research company (MDRC) processed the data merged by ICF and produced an analytic grades file, blind to the consequences of their decisions for the estimated treatment effects, as described in Supplementary Information section 12. Those data were shared with the authors of this paper, who analysed the data following a pre-registered analysis plan (see Supplementary Information section 13; MDRC will later produce its own independent report using its processed data, and retained the right to deviate from our pre-analysis plan).

Selection of schools was stratified by school achievement and minority composition. A simple random sample would not have yielded sufficient numbers of rare types of schools, such as high-minority schools with medium or high levels of achievement. This was because school achievement level—one of the two candidate moderators—was strongly associated with school racial/ethnic composition^46^ (percentage of Black/African-American or Hispanic/Latino/Latina students, *r* = −0.66).

A total of 139 schools were selected without replacement from a sampling frame of roughly 12,000 regular US public high schools, which serve the vast majority of students in the United States. Regular US public schools exclude charter or private schools, schools serving speciality populations such as students with physical disabilities, alternative schools, schools that have fewer than 25 ninth-grade students enrolled and schools in which ninth grade is not the lowest grade in the school.

Of the 139 schools, 65 schools agreed, participated and provided student records. Another 11 schools agreed and participated but did not provide student grades or course-taking records; therefore, the data of their students are not analysed here. School nonresponse did not appear to compromise representativeness. We calculated the Tipton generalizability index^47^, a measure of similarity between an analytic sample and the overall sampling frame, along eight student demographic and school achievement benchmarks obtained from official government sources^27^. The index ranges from 0 to 1, with a value of 0.90 corresponding to essentially a random sample. The National Study of Learning Mindsets showed a Tipton generalizability index of 0.98, which is very high (see Supplementary Information section 3).

Within schools, the average student response rate for eligible students was 92% and the median school had a response rate of 98% (see definitions in Supplementary Information section 5). This response rate was obtained by extensive efforts to recruit students into make-up sessions if students were absent and it was aided by a software system, developed by the technology vendor (PERTS), that kept track of student participation. A high within-school response rate was important because lower-achieving students, our target group, are typically more likely to be absent.

### Growth mindset intervention content

In preparing the intervention to be scalable, we revised past growth mindset interventions to focus on the perspectives, concerns and reading levels of ninth-grade students in the United States, through an intensive research and development process that involved interviews, focus groups and randomized pilot experiments with thousands of adolescents^13^.

The control condition, focusing on brain functions, was similar to the growth mindset intervention, but did not address beliefs about intelligence. Screenshots from both interventions can be found in Supplementary Information section 4, and a detailed description of the general intervention content has previously been published^13^. The intervention consisted of two self-administered online sessions that lasted approximately 25 min each and occurred roughly 20 days apart during regular school hours (Fig. 1).

The growth mindset intervention aimed to reduce the negative effort beliefs of students (the belief that having to try hard or ask for help means you lack ability), fixed-trait attributions (the attribution that failure stems from low ability) and performance avoidance goals (the goal of never looking stupid). These are the documented mediators of the negative effect of a fixed mindset on grades^12,15,48^ and the growth mindset intervention aims to reduce them. The intervention did not only contradict these beliefs but also used a series of interesting and guided exercises to reduce their credibility.

The first session of the intervention covered the basic idea of a growth mindset—that an individual’s intellectual abilities can be developed in response to effort, taking on challenging work, improving one’s learning strategies, and asking for appropriate help. The second session invited students to deepen their understanding of this idea and its application in their lives. Notably, students were not told outright that they should work hard or employ particular study or learning strategies. Rather, effort and strategy revision were described as general behaviours through which students could develop their abilities and thereby achieve their goals.

The materials presented here sought to make the ideas compelling and help adolescents to put them into practice. It therefore featured stories from both older students and admired adults about a growth mindset, and interactive sections in which students reflected on their own learning in school and how a growth mindset could help a struggling ninth-grade student next year. The intervention style is described in greater detail in a paper reporting the pilot study for the present research^13^ and in a recent review article^12^.

Among these features, our intervention mentioned effort as one means to develop intellectual ability. Although we cannot isolate the effect of the growth mindset message from a message about effort alone, it is unlikely that the mere mention of effort to high school students would be sufficient to increase grades and challenge seeking. In part this is because adolescents often already receive a great deal of pressure from adults to try hard in school.

### Intervention delivery and fidelity

The intervention and control sessions were delivered as early in the school year as possible, to increase the opportunity to set in motion a positive self-reinforcing cycle. In total 82% of students received the intervention in the autumn semester before the Thanksgiving holiday in the United States (that is, before late November) and the rest received the intervention in January or February; see Supplementary Information section 5 for more detail. The computer software of the technology vendor randomly assigned adolescents to intervention or control materials. Students also answered various survey questions. All parties were blind to condition assignment, and students and teachers were not told the purpose of the study to prevent expectancy effects.

The data collection procedures yielded high implementation fidelity across the participating schools, according to metrics listed in the pre-registered analysis plan. In the median school, treated students viewed 97% of screens and wrote a response for 96% of open-ended questions. In addition, in the median school 91% students reported that most or all of their peers worked carefully and quietly on the materials. Fidelity statistics are reported in full in Supplementary Information section 5.6; Extended Data Table 2 shows that the treatment effect heterogeneity conclusions were unchanged when controlling for the interaction of treatment and school-level fidelity as intended.

### Measures

#### Self-reported fixed mindset

Students indicated how much they agreed with three statements such as “You have a certain amount of intelligence, and you really can’t do much to change it” (1, strongly disagree; 6, strongly agree). Higher values corresponded to a more fixed mindset; the pre-analysis plan predicted that the intervention would reduce these self-reports.

*GPAs*. Schools provided the grades of each student in each course for the eight and ninth grade. Decisions about which courses counted for which content area were made independently by a research company (MDRC; see Supplementary Information section 12). The GPAs are a theoretically relevant outcome because grades are commonly understood to reflect sustained motivation, rather than only prior knowledge. It is also a practically relevant outcome because, as noted, GPA is a strong predictor of adult educational attainment, health and well-being, even when controlling for high school test scores^38^.

#### School achievement level

The school achievement level moderator was a latent variable that was derived from publicly available indicators of the performance of the school on state and national tests and related factors^45,46^, standardized to have mean = 0 and s.d. = 1 in the population of the more than 12,000 US public schools.

#### Behavioural challenge-seeking norms of the schools

The challenge-seeking norm of each school was assessed through a behavioural measure called the make-a-math-worksheet task^13^. Students completed the task towards the end of the second session, after having completed the intervention or control content. They chose from mathematical problems that were described either as challenging and offering the chance to learn a lot or as easy and not leading to much learning. Students were told that they could complete the problems at the end of the session if there was time. The school norm was estimated by taking the average number of challenging mathematical problems that adolescents in the control condition attending a given school chose to work on. Evidence for the validity of the challenge-seeking norm is presented in the Supplementary Information section 10.

#### Norms of self-reported mindset of the schools

A parallel analysis focused on norms for self-reported mindsets in each school, defined as the average fixed mindset self-reports (described above) of students before random assignment. The private beliefs of peers were thought to be less likely to be visible and therefore less likely to induce conformity and moderate treatment effects, relative to peer behaviours^49^; hence self-reported beliefs were not expected to be significant moderators. Self-reported mindset norms did not yield significant moderation (see Extended Data Table 2).

#### Course enrolment to advanced mathematics

We analysed data from 41 schools who provided data that allowed us to calculate rates at which students took an advanced mathematics course (that is, algebra II or higher) in tenth grade, the school year after the intervention. Six additional schools provided tenth grade course-taking data but did not differentiate among mathematics courses. We expected average effects of the treatment on challenging course taking in tenth grade to be small because not all students were eligible for advanced mathematics and not all schools allow students to change course pathways. However, some students might have made their way into more advanced mathematics classes or remained in an advanced pathway rather than dropping to an easier pathway. These challenge-seeking decisions are potentially relevant to both lower- and higher-achieving students, so we explored them in the full sample of students in the 41 included schools.

### Analysis methods

#### Overview

We used intention-to-treat analyses; this means that data were analysed for all students who were randomized to an experimental condition and whose outcome data could be linked. A complier average causal effects analysis yielded the same conclusions but had slightly larger effect sizes (see Supplementary Information section 9). Here we report only the more conservative intention-to-treat effect sizes. Standardized effect sizes reported here were standardized mean difference effect sizes and were calculated by dividing the treatment effect coefficients by the raw standard deviation of the control group for the outcome, which is the typical effect size estimate in education evaluation experiments. Frequentist *P* values reported throughout are always from two-tailed hypothesis tests.

#### Model for average treatment effects

Analyses to estimate average treatment effects for an individual person used a cluster-robust fixed-effects linear regression model with school as fixed effect that incorporated weights provided by statisticians from ICF, with cluster defined as the primary sampling unit. Coefficients were therefore generalizable to the population of inference, which is students attending regular public schools in the United States. For the *t* distribution, the degrees of freedom is 46, which is equal to the number of clusters (or primary sampling units, which was 51) minus the number of sampling strata (which was 5)^45^.

#### Model for the heterogeneity of effects

To examine cross-school heterogeneity in the treatment effect among lower-achieving students, we estimated multilevel mixed effects models (level 1, students; level 2, schools) with fixed intercepts for schools and a random slope that varied across schools, following current recommended practices^39^. The model included school-centred student-level covariates (prior performance and demographics; see the Supplementary Information section 7) to make site-level estimates as precise as possible. This analysis controlled for school-level average student racial/ethnic composition and its interaction with the treatment status variable to account for confounding of student body racial/ethnic composition with school achievement levels. Student body racial/ethnic composition interactions were never significant at *P* < 0.05 and so we do not discuss them further (but they were always included in the models, as pre-registered).

#### Bayesian robustness analysis

A final pre-registered robustness analysis was conducted to reduce the influence of two possible sources of bias: awareness of study hypotheses when conducting analyses and misspecification of the regression model (see the Supplemental Information, section 13, p. 12). Statisticians who were not involved in the study design and unaware of the moderation hypotheses re-analysed a blinded dataset that masked the identities of the variables. They did so using an algorithm that has emerged as a leading approach for understanding moderators of treatments: BCF^40^. The BCF algorithm uses machine learning tools to discover (or rule out) higher-order interactions and nonlinear relations among covariates and moderators. It is conservative because it uses regularization and strong prior distributions to prevent false discoveries. Evidence for the robustness of the moderation analysis in our pre-registered model comes from correspondence with the estimated moderator effects of BCF in the part of the distribution where there are the most schools (that is, in the middle of the distribution), because this is where the BCF algorithm is designed to have confidence in its estimates (Extended Data Fig. 3).

### Reporting summary

Further information on research design is available in the Nature Research Reporting Summary linked to this paper.

## Online content

Any methods, additional references, Nature Research reporting summaries, source data, extended data, supplementary information, acknowledgements, peer review information; details of author contributions and competing interests; and statements of data and code availability are available at 10.1038/s41586-019-1466-y.

### Supplementary information

Supplementary Information This file contains Supplementary Methods and Information – see the Supplementary Contents page for full details.

Reporting Summary

### Source data

Source Data Fig. 2

Source Data Extended Data Fig. 1

Source Data Extended Data Fig. 2

Source Data Extended Data Fig. 3

## Figures and Tables

**Extended Data Table 1:**
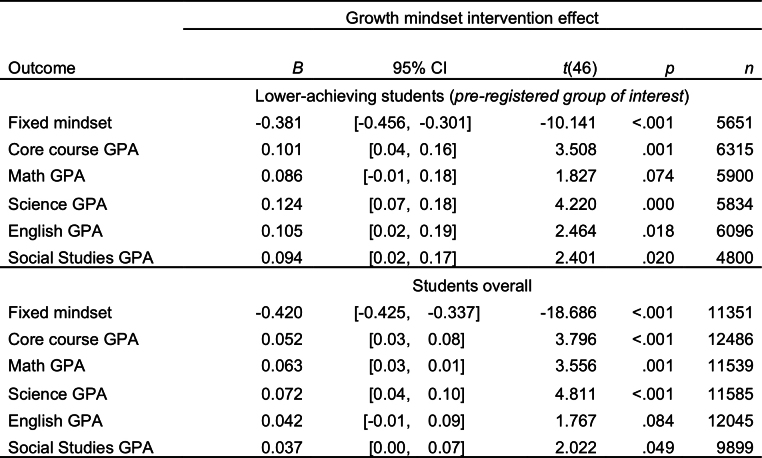
Growth mindset effects were of a similar magnitude across subject areas

*P* values are from two-sided hypothesis tests. Confirming the predictions in the pre-analysis plan, higher-achieving students demonstrated no significant treatment effects on core course GPAs, *B =* 0.01 grade points (95% confidence interval = −0.03–0.06), s.e. = 0.02, *n* = 6,170, *k* = 65, *t* = 0.480, *P* = 0.634, standardized mean difference effect size = 0.01, resulting in a significant intervention × lower-achiever interaction *B =* 0.09 grade points (95% confidence interval = 0.01–0.17), s.e. = 0.04, *n* = 12,490, *k* = 65, *t* = 2.179, *P* = 0.034). This result replicates previous research and supports the pre-registered decision to examine average GPA effects only among lower-achieving students, as higher-achieving students may have already had habits (for example, turning in work on time) and environments (for example, supportive family, teachers or peer groups) that fostered high GPAs even in the control condition.

**Extended Data Table 2:**
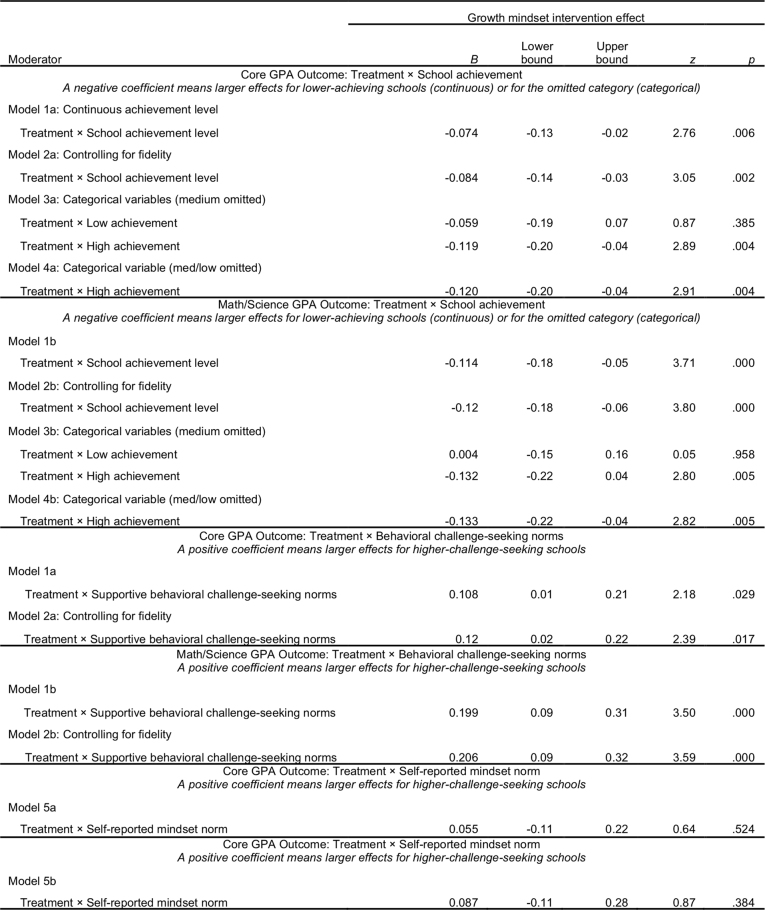
Moderating effects of school achievement level and school norms

Values are from the pre-registered model (see Supplementary Information section 7, RQ4). Moderator estimates with the same model number and letter combination were obtained from the same regression model. *P* values are from two-tailed hypothesis tests.

**Extended Data Table 3:**
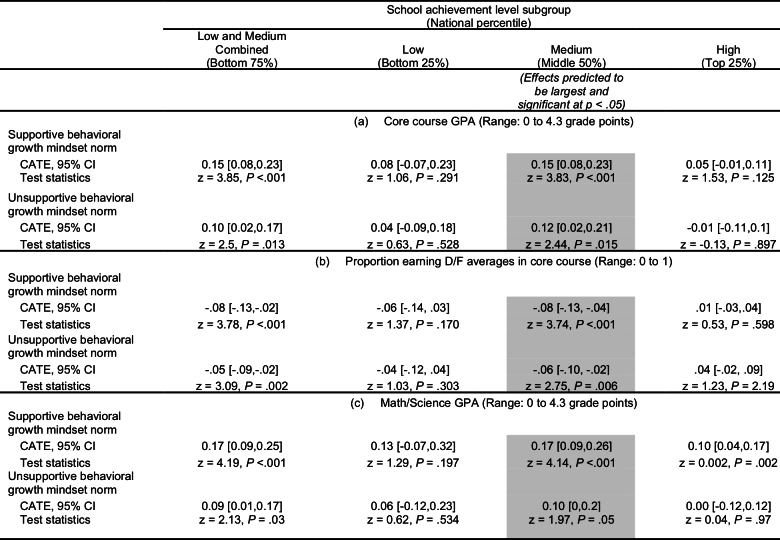
CATEs are largest for medium-achieving schools with supportive norms

CATEs are the average differences between the randomly assigned intervention and control groups in terms of GPA or D/F average rates, for a given set of schools. 95% CI, 95% confidence interval. *P* values are from two-tailed hypothesis tests. Norms refers to behavioural challenge-seeking norms, as measured by the responses of the control group to the make-a-math-worksheet task. Standardized effect sizes for GPA are essentially identical to the unstandardized effect sizes because the standard deviation of GPA is approximately 1. The estimates were generated from the pre-registered linear mixed-effects regressions (equations provided in Supplementary Information section 7) that used survey weights provided by the research company to make estimates generalizable. The models included three school-level moderators of the student-level randomized treatment: the achievement level (categorical, dummies for low and high, medium group omitted), the behavioural growth mindset norms (continuous) and the percentage of racial or ethnic minority students (continuous) of the school. To define the school achievement levels for presentation of school subgroup effects, we followed the analysis plan. The pre-analysis plan did not include a method for post-estimation summarization of the effects of the continuous norms, so the table uses a prominent default: a split at the population median. The full, continuous norms variable was used to estimate the model, so the choice of the median split cut-off point did not affect the estimation of the regression coefficients. Grey shaded columns indicate the subgroup that was expected to have the largest effects in the pre-registered analysis plan. **a**, School achievement level subgroups for core course GPAs. **b**, School achievement level subgroups for reduction in rates of D/F averages in core course GPAs. **c**, School achievement level subgroups for GPAs of only mathematics and science.

## Data Availability

Technical documentation for the National Study of Learning Mindsets is available from ICPSR at the University of Michigan (10.3886/ICPSR37353.v1). Aggregate data are available at https://osf.io/r82dw/. Student-level data are protected by data sharing agreements with the participating districts; de-identified data can be accessed by researchers who agree to terms of data use, including required training and approvals from the University of Texas Institutional Review Board and analysis on a secure server. To request access to data, researchers should contact mindset@prc.utexas.edu. The pre-registered analysis plan can be found at https://osf.io/tn6g4. The intervention module will not be commercialized and will be available at no cost to all secondary schools in the United States or Canada that wish to use it via https://www.perts.net/. Selections from the intervention materials are included in the Supplementary Information. Researchers wishing to access full intervention materials should contact mindset@prc.utexas.edu and must agree to terms of use, including non-commercialization of the intervention.

## Code availability

Syntax can be found at https://osf.io/r82dw/ or by contacting mindset@prc.utexas.edu.
