# Supplementary material for: A national experiment reveals where a growth mindset improves achievement
Source: Nature. 2019 Aug 7;573(7774):364–9. doi: 10.1038/s41586-019-1466-y (PMC6786290; doi:10.1038/s41586-019-1466-y)
Supplement: Supplementary file 1 — This file contains Supplementary Methods and Information – see the Supplementary Contents page for full details. [file 41586_2019_1466_MOESM1_ESM.pdf]

nature
SUPPLEMENTARY INFORMATION
https://doi.org/10.1038/s41586-019-1466-y
In the format provided by the authors and unedited.

A national experiment reveals where a growth mindset improves achievement

David S. Yeager[1]*, Paul Hanselman[2]*, Gregory M. Walton[3], Jared S. Murray[1], Robert Crosnoe[1], Chandra Muller[1], Elizabeth Tipton[4], Barbara Schneider[5], Chris S. Hulleman[6], Cintia P. Hinojosa[7], David Paunesku[8], Carissa Romero[9], Kate Flint[10], Alice Roberts[10], Jill Trott[10], Ronaldo Iachan[10], Jenny Buontempo[1], Sophia Man Yang[1], Carlos M. Carvalho[1], P. Richard Hahn[11], Maithreyi Gopalan[12], Pratik Mhatre[1], Ronald Ferguson[13], Angela L. Duckworth[14] & Carol S. Dweck[3]

[1]University of Texas at Austin, Austin, TX, USA. [2]University of California, Irvine, Irvine, CA, USA. [3]Stanford University, Stanford, CA, USA. [4]Northwestern University, Evanston, IL, USA. [5]Michigan State University, East Lansing, MI, USA. [6]University of Virginia, Charlottesville, VA, USA. [7]University of Chicago, Chicago, IL, USA. [8]Project for Education Research that Scales, San Francisco, CA, USA. [9]Paradigm Strategy Inc., San Francisco, CA, USA. [10]ICF, Fairfax, VA, USA. [11]Arizona State University, Tempe, AZ, USA. [12]The Pennsylvania State University, University Park, PA, USA. [13]Harvard University, Cambridge, MA, USA. [14]University of Pennsylvania, Philadelphia, PA, USA. *e-mail: dyeager@utexas.edu; paul.hanselman@uci.edu

Methods and Supplementary Information

June, 2019

Contents

1 Document Information 2
- 1.1 Version Date 2
- 1.2 Main text 2
- 1.3 Overview 2

2 Research Questions and Concordance with Pre-Registered Analysis Plan 3

3 Representativeness of the Analytic Sample 6
- 3.1 Comparisons of Covariates Across the Analytic Sample and National Sampling Frame 7
- 3.2 Generalizability Index 8

4 Details of the Study Procedure and Data Collection 9
- 4.1 Overview of the Student Participation Process 9
- 4.2 Illustrative Screenshots from the Growth Mindset Intervention 10
- 4.3 Illustrative Screenshots from the Control Condition 15
- 4.4 Student Responses to Three Key Treatment Open Response Prompts 18

5 Implementation of the Intervention in the National Sample 23
- 5.1 Summary of Implementation and Implications 23
- 5.2 Student Survey Response Rates 23
- 5.3 Timing of Intervention Sessions Within the School Year 24
- 5.4 How Long Did Students Spend on the Intervention Exercises? 26
- 5.5 Session Completion 27
- 5.6 Implementation Fidelity 28

6 Data Description 29
- 6.1 Defining Key Variables 29
- 6.2 Summary of Full Sample (unweighted) 29
- 6.3 Descriptive Statistics for Previously Lower-achieving Students (unweighted) 30
- 6.4 Descriptive Statistics for Previously Higher-achieving Students (unweighted) 31
- 6.5 GPA Distributions for Previously Lower- and Higher-Achieving Students 32
- 6.6 Additional Information on Components of the Lower-achieving Designation 33
- 6.7 Experimental Balance on Pre-treatment Characteristics 34
- 6.8 Rates of Attrition 35
- 6.9 Differential Characteristics of Students who Attritted Versus Those Who Did Not 35
- 6.10 Balance for Students with Outcome Information 36
- 6.11 Sample Calculations for CONSORT Report 37

7 Pre-Registered Intervention Impacts on Academic Grade Point Average (GPA) 38
- 7.1 Average Intervention Effects for All Students (Pre-registered RQ1) 38
- 7.2 Conditional Average Intervention Effect for Previously Lower-achieving Students (Pre-registered RQ 2) 38
- 7.3 Robustness and Sensitivity Analyses for RQ1 and RQ2 39
- 7.4 School Heterogeneity (Pre-registered RQ3) 41
- 7.5 Theoretical Justification for School Moderation Analysis (Pre-registered RQ4) 42
- 7.6 School Moderator Definitions and Analyses (Pre-registered RQ4) 43
- 7.7 Pre-registered Mixed Effects Regression Models Testing School-level Moderators (RQ4) 45

8 Multilevel Bayesian Causal Forest Model **45**

9 Complier Average Causal Effects **47**
9.1 Estimated CACE on Core Academic GPA for All Compliers 48
9.2 Estimated CACE on Core Academic GPA for Compliers Among Previously Lower-achieving Students 48

10 Methodological Information About the School Challenge-seeking Norms Measure **49**
10.1 Distribution of School Mindset Norms Measure (Average Number of "Hard" Items Selected by Schools' Control Group) 49
10.2 Mindset Norms and Mathematics AP: All schools 50
10.3 Mindset Norms and Mathematics AP: Schools with AP Data Available Only 52

11 Cohort Analysis of i3 Evaluation Effect Sizes **53**

12 Independent Contractor's Methods for Processing the Grades Variables **54**
12.1 Overview 54
12.2 Phase 1: Coding Course Names 54
12.3 Phase 2: Calculating GPAs 55

13 Pre-registration File **55**

1 Document Information

1.1 Version Date

This document compiled on 2019-06-20 19:20:28.

1.2 Main text

This document provides supplementary information for the main text:

See manuscript for full author list, affiliations, and notes. Address correspondence to David S. Yeager (dyeager@utexas.edu) or Paul Hanselman (paul.hanselman@uci.edu).

1.3 Overview

This document provides methodological information and additional detail about the National Study of Learning Mindsets.

In the Research Questions section, we print the research questions from our pre-registered anlaysis plans.

In the Representativeness section, we compare the National Study of Learning Mindserts sample to the sampling frame to assess the representativeness of this sample.

In the Intervention section, we present methodological details of the intervention, including example screenshots and students' responses.

In the Implementation section, we provide methodological details of implementation and participation in the study.

In the Data Description section, we present descriptive statistics for the achieved sample, balance tests of the effectiveness of random assignment, and attrition.

In the Methods for Pre-registered Analyses section, we present methodological details for how we answered our primary research questions.

In the Methods for CACE Analyses section, we present methods for how we estimated the complier average causal effects, representing the effect of the treatment on the treated, in contrast to the pre-specified intent to treat estimates.

In the Mindset Norms Validity section, we present methodological information about the validity of the school mindset norms moderator measure.

In the Pre-registration File section, we reproduce the full pre-registered analysis plan, which is also available at: https://osf.io/afmb6/

2 Research Questions and Concordance with Pre-Registered Analysis Plan

Below, we list what was planned as an analysis and what we did.

RQ 1: What is the average treatment effect (ATE) of a Growth Mindset (GM) intervention on the GPA of 9th grade students in regular U.S. public high schools?

- The ATE was "expected to be very small and positive" (analysis plan page 2, footnote 1) and was not expected to be statistically significant (see the decision table on page 10 of the analysis plan)
- The analysis plan (p. 10) stated that we would estimate cluster-robust school-fixed-effects regressions with survey weights, and this was done.
- Results showed that the ATE was small and positive, but, surprisingly, it was statistically significantly different from zero.

RQ 2: What is the conditional average treatment effect (CATE) of a GM intervention on the GPA of 9th grade previously low-performing students in regular U.S. public high schools?

- According to the pre-registered analysis plan (pre-registration page 2, footnote 1, decision table on page 10), *"The effect for previously low-performers [was] expected to be moderate positive, relatively larger than for the full sample, and statistically significant."*
- The analysis plan (p. 10) stated that we would estimate cluster-robust school-fixed-effects regressions with survey weights, and this was done.
- The expected results appeared, as shown in the main text.
- Robustness analyses for this question are presented in the Extended Data.

Thus, the data for RQs 1 and 2 led to this conclusion from the decision-rule table on p. 10 of the analysis plan: *"[We] replicated the Yeager/Paunesku low-performer effect and surprisingly showed a main effect as well. Program was effective, on average, for the full sample and for previously low performers."*

RQ 3: How much does the CATE of a GM intervention (on the GPA of 9th grade previously low-performing students) vary across U.S. public high schools?

- We hypothesized that there would be significant cross-site variation (pre-registration p. 2) and this was the case, as shown in the main text.

- The analysis plan (p. 11) stated that we would test a "hybrid" mixed effects model (school fixed intercepts and random slope), and this is what was estimated.

RQ 4: Do school-level factors explain the variability in the size of the CATE of the GM (on GPA for previously low-performing students in U.S. public high schools)?

- We hypothesized that the intervention effect would vary with respect to two factors: school achievement level and school challenge-seeking norms (called "mindset saturation" in the analysis plan, p. 2).
- The analysis plan stated that we would test a "hybrid" mixed effects model (school fixed intercepts and random slope), and this is what was estimated.
- The results of this analysis are reported in the Extended Data; both hypothesized moderators were significantly associated with the size of the treatment effect.
- The analysis plan (p. 9) stated that we predicted moderation by behavioral norms but not by self-reported norms, and the manuscript reports this finding.
- Planned follow-up analyses comparing low and high-achieving schools to medium-achieving schools), described in the analysis plan (section 18, page 12) appear in the Extended Data.
- A planned robustness analysis, which involved providing statisticians with a blinded dataset so they could conducted non-parametric analyses of the moderators (as described in section 18, page 12 of the analysis plan) was conducted via BCF. See model output in the Extended Data.

Follow-up analyses. The pre-registered analysis plan called for several follow-up analyses (section 20, page 12):

- The plan stated that we would take steps to address the robustness of the assumptions of the linear model, namely the homoskedasticity assumption. The models reported below did this by calculating heteroskedasticity-robust standard errors using defaults in the StataSE software.
- The plan stated that non-parametric models would explore the potential different school achievement subgroups, and this is done in the "Bayesian Causal Forest" analysis reported in section 8 below.
- The plan stated that we would assess the representativeness of the participating schools and the potential to generalize to the population, and we do so in section 3 below.

Planned exploratory analyses that were conducted. We stated that these "will be reported in the manuscript or supplement regardless of the outcomes" (analysis plan p. 13).

- The analysis plan stated that we would report treatment effects on the "poor performance rate" (rates of earning D/F averages). This is reported in the manuscript.
- The analysis plan stated that we would report results for separate subjects, and this is done in the Extended DAta.
- The analysis plan also stated that we hypothesized stronger results especially for math and science grades, and this hypothesis was true (with respect to the norms moderation finding) and so we report these results in the manuscript and in the Extended Data.
- The analysis plan stated that we would compute 5 metrics of schools' success at implementing the treatment with high fidelity, that we would aggregate those metrics into a single school-level measure, and that we would test whether this aggregated measure changes the primary heterogeneity findings. The school-level fidelity metrics are reported in the manuscript and in section 5.6 below. Analyses that test the robustness of our moderation conclusions to differences in the fidelity of implementation are presented in the Extended Data; these find that fidelity does not explain the primary moderation results. However this exploratory analysis is preliminary.
- The analysis plan stated that we would explore whether cross-site heterogeneity in the strength of the intervention effect on the manipulation check might explain cross-site heterogeneity in the effects of the intervention on GPA. We report in the manuscript that we did not find significant cross-site heterogeneity in the size of the intervention effect on the manipulation check. Neither the self-reported mindsets, reported below, nor self-reported challenge-seeking, which is a single item reported in Yeager et al., 2016 and not discussed here for parsimony), showed significant variability.

Exploratory analyses that were not conducted. A second set of "planned exploratory analyses" were described in this way (pages 14-15): *"They could be presented as secondary analyses for the primary paper, or they could constitute papers of their own."* In all but one case, we do not answer the second set of planned exploratory analyses because these were beyond the scope of the paper. The only one of these planned exploratory analyses we conducted was #6 ("Interaction of achievement level and mindset saturation"). The "Bayesian Causal Forest" analysis allowed for this interaction and did not find it.

Deviations from the analysis plan. These are the minor deviations from the plan; none substantively impact the main results or conclusions of the study.

- We expected to receive grades data from 66 schools but one school provided only baseline (not post-intervention) data, so the number of schools was 65.

- On page 11 of the analysis plan we stated that we would conduct a permutation test of the variability of the treatment effect across schools, to test its significance, but an expert raised questions about the validity of that test, so we rely on the Q statistic instead (which was also pre-registered on page 11). That test was significant and yielded the same conclusions.
- On the same page we stated that we would compare our cross-site variability statistic to published benchmarks, but this may not be valid because not all past studies were universal prevention studies. So although the current estimate of variability in GPA impacts would be at the higher end of the distribution of the effects reported by Weiss et al,. (2017) in Table 1C., we refrain from explicit comparisons because it would be difficult to justify that they are appropriate.
- On page 12 of the analysis plan we stated that we would test whether there is a significant reduction in cross-site variability in the treatment effect after inclusion of the moderators; however we were not able

to find a satisfactory and valid test of this difference, so we did not conduct that analysis.

Other analysis notes. Unless otherwise noted, analyses employ weights to represent the population of regular public high schools in the U.S. (as pre-specified). Weights were provided by the survey research firm. Robustness analyses (see Extended Data) examine the impact of weights on the results.

In the main text and extended data, we present regression coefficients in terms of unstandardized effect sizes to make it easier to translate impact in terms of the natural metrics of a 0 to 4.0 GPA or the % of students prevented from failing. The standardized effect sizes we present are "Glass's Delta," defined as the group mean difference divided by the control group's standard deviation.

3 Representativeness of the Analytic Sample

Because the National Study of Learning Mindsets (NSLM) had a school response rate of 56%, we evaluated whether site-level non-response compromised the generalizability of the sample. We did so by carrying out a benchmark analysis to assess the representativeness of the NSLM analytic sample relative to the national sampling frame. Here we provide a summary of this comprehensive benchmarkikng analysis. More details are reported in a technical paper on this topic (see Gopalan, 2018[1]).

The school- and district-level benchmarks were obtained from publicly-available data such as the Common Core of Data (CCD)[2], the Office of Civil Rights (OCR)[3], and a school district-level tabulation of American Community Survey (ACS) data[4]. We obtained this information for the sampling frame, which included all regular, U.S. public high schools with at least 25 students in 9th grade and in which 9th grade is the lowest grade.

The NSLM analytic sample had a high degree of similarity to the inference population via two metrics. First, comparisons of school- and district-level characteristics between the NSLM analytic sample and the inference population find few statistically significant differences, as shown below. Second, applying an empirical method to quantify the degree of generalizability, the Tipton (2014 [5]) generalizability index, found that the analytic sample is highly generalizable to the population.

[1]Gopalan, M. (2018 preprint). Is the National Study of Learning Mindsets Nationally-Representative of 9th Graders in the US?. Retrieved from https://psyarxiv.com/dvmr7/

[2]National Center for Education Statistics. (2018a, September 6). Common Core of Data (CCD). Retrieved September 6, 2018, from https://nces.ed.gov/ccd/pubschuniv.asp

[3]U.S. Department of Education, Office for Civil Rights. (2018, September 6). Civil Rights Data Collection (CRDC). Retrieved September 6, 2018, from https://ocrdata.ed.gov/

[4]National Center for Education Statistics. (2018b, September 12). Education demographics and geographic estimates: American Community Survey (ACS). Retrieved from https://nces.ed.gov/programs/edge/Demographic/ACS

[5]Tipton, E. (2014). How Generalizable Is Your Experiment? An Index for Comparing Experimental Samples and Populations. *Journal of Educational and Behavioral Statistics*, 39(6), 478-501

3.1 Comparisons of Covariates Across the Analytic Sample and National Sampling Frame

Benchmarks	Sampling_Frame_Mean	NSLM_Analytic_Mean	SMD	p
Proportion of 9th Grade Male Students	0.52	0.5	0.02	0.109
Proportion of 9th Grade Asian Students	0.04	0.02	0.02	0.028
Proportion of 9th Grade Black Students	0.14	0.16	-0.02	0.625
Proportion of 9th Grade Hispanic Students	0.21	0.19	0.02	0.688
Proportion of 9th Grade White Students	0.57	0.58	-0.01	0.815
Proportion of 9th Grade Other Race Students	0.05	0.05	0	0.564
Total 9th Grade Enrollment	285	297	-0.03	0.811
Proportion of High School Students Enrolled in Algebra 1	0.22	0.22	0	0.887
Proportion of High School Students Enrolled in Algebra 2	0.2	0.21	-0.01	0.333
Proportion of High School Students Enrolled in at least one AP course	0.19	0.2	-0.01	0.692
Proportion of High School Students who took at least 1 AP Exam	0.7	0.68	0.02	0.633
Proportion of Students who are Chronically Absent	0.21	0.2	0.01	0.736

Notes: SMD is the standardized mean difference. For proportions we report the absolute differences. A small number of schools do not have information available from the CCD and/or CRDC. Those schools are excluded from the mean calculation for missing benchmarks, as appropriate. All data shown in the above table were obtained from the same sources and were not estimated from student-level data to maintain valid comparisons. Only a subset of all benchmarks analyzed is shown in the table above to economize on space. The analytic sample means are adjusted to include school-level weights given the known probability of school selection from the sampling frame. P-values shown from one-sample t-tests comparing mean differences

3.2 Generalizability Index

The generalizability index (Tipton, 2014) is a summary measure that provides the degree of distributional similarity between the schools in the analytic sample and the inference population, conditional on a set of covariates. The index is calculated using propensity scores from a sampling propensity score model, which predicts membership into the analytic sample, given a set of observed school-level characteristics, using logistic regression. The generalizability index takes on values between 0 and 1, where a value of 0 means that the analytic sample and inference population are completely different and a value of 1 indicates that the analytic sample is an exact miniature of the inference population on the selected covariates (i.e., all standardized mean differences for the covariates are 0). Please see Tipton (2014) for more details regarding the motivation, proofs, and empirical validity of this index in making generalizability claims. This index is estimated using kernel densities and the R code provided by Tipton (2014) in her online supplement.

Based on a simulation study, Tipton (2014) recommends that experimental samples with generalizability-indices greater than 0.90 can be considered to be as good as a random sample from the population of interest, conditional on the covariates included in the sampling propensity score model. The Table below shows that the generalizability index is .98. Additionally, Gopalan (2018) finds that the analytic sample is similar to four other theoretically-relevant inference populations identified based on school achievement categories and the proportion of stereotyped minority students in school (high vs. low). This is important because our paper seeks to make inferences about conditional average treatment effects within these subgroups. In all, we find that site-level non-response does not compromise the generalizability of the results from the achieved sample of schools in the NSLM.

Inference_Population	Generalizabillity_Index	N_Inference_Population	N_NSLM_Analytic_Sample
All Public High Schools (9th grade lowest level)	0.982	9515	59

Notes: The full NSLM analytic sample that provided grades includes 65 schools, but schools must be omitted from the calculations due to missing values on one or more benchmarks. N refers to the number of schools with non-missing benchmarks used in the sampling propensity score model. The schools in the sampling frame were compared to those in the analytic sample of the NSLM based on the following benchmarks: racial composition (%African American, %Hispanic, %White), socioeconomic composition (%Free/Reduced-Price Lunch Recipients), gender composition (%Male), the number of students in the school, the proportion of students in the district that are English Language Learners in the district, and the proportion of Special Education students in the district.

4 Details of the Study Procedure and Data Collection

4.1 Overview of the Student Participation Process

The participation process is depicted in the Study Overview Figure below. As it shows, the design of the study called for schools to deliver both sessions of the intervention to all students in the school in the fall of 2015, and for the two sessions to be roughly three weeks apart.

A school liason worked with a member of the data collection firm to select teachers who would devote two class periods non-academic to the study. There was no restriction on type of class, and often non-academic subjecs such as PE or Music were selected. Teachers brought their students to the school's computer lab during normal class time and read a brief script explaining that students were about to participate in a study. The study was described as a part of a research project that entailed a survey about the transition to high school. Students then signed into the research website and were randomized by the web server to a growth mindset condition or a "brain basics" control condition. Every person involved in the study was blind to condition assignment throughout the study (indeed, there was no way for a school staff person or research team member to access that information).

Students in the growth mindset condition: (1) were presented with information about neural plasticity that emphasized how brain functions can improve when one confronts new challenges and practices more difficult ways of thinking, (2) completed writing exercises designed to help students understand and internalize the intervention message by applying the message to their own life and restating the message for a future student. For examples of the intervention materials and student writing, see below.

Students in the control group, like those in the intervention group, read a brief article about the brain and answer reflective questions. However, they did not learn about the brain's malleability. Instead, they learn about basic brain functions and their localization, for example, the key functions associated with each cortical lobe. The experimental conditions were designed to look very similar so that students' instructors would remain blind to their condition assignment, and to discourage students from comparing their materials.

4.1.1 Study Overview

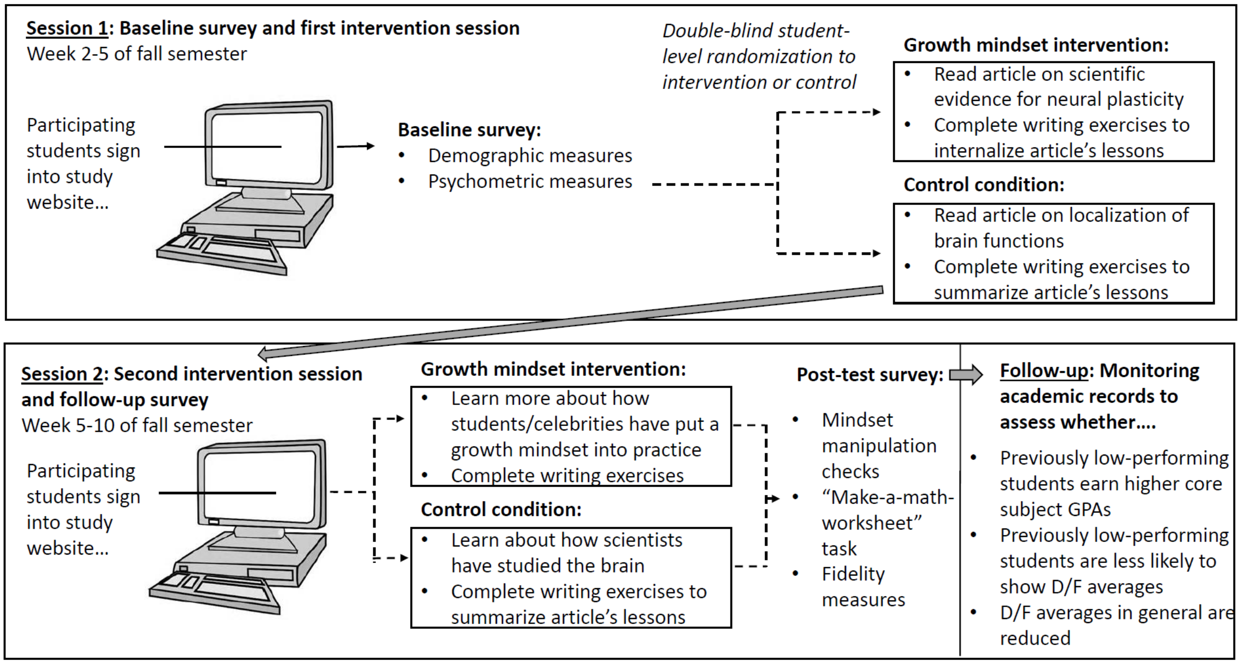

4.2 Illustrative Screenshots from the Growth Mindset Intervention

4.2.1 Treated students are presented with information about the malleability of the brain.

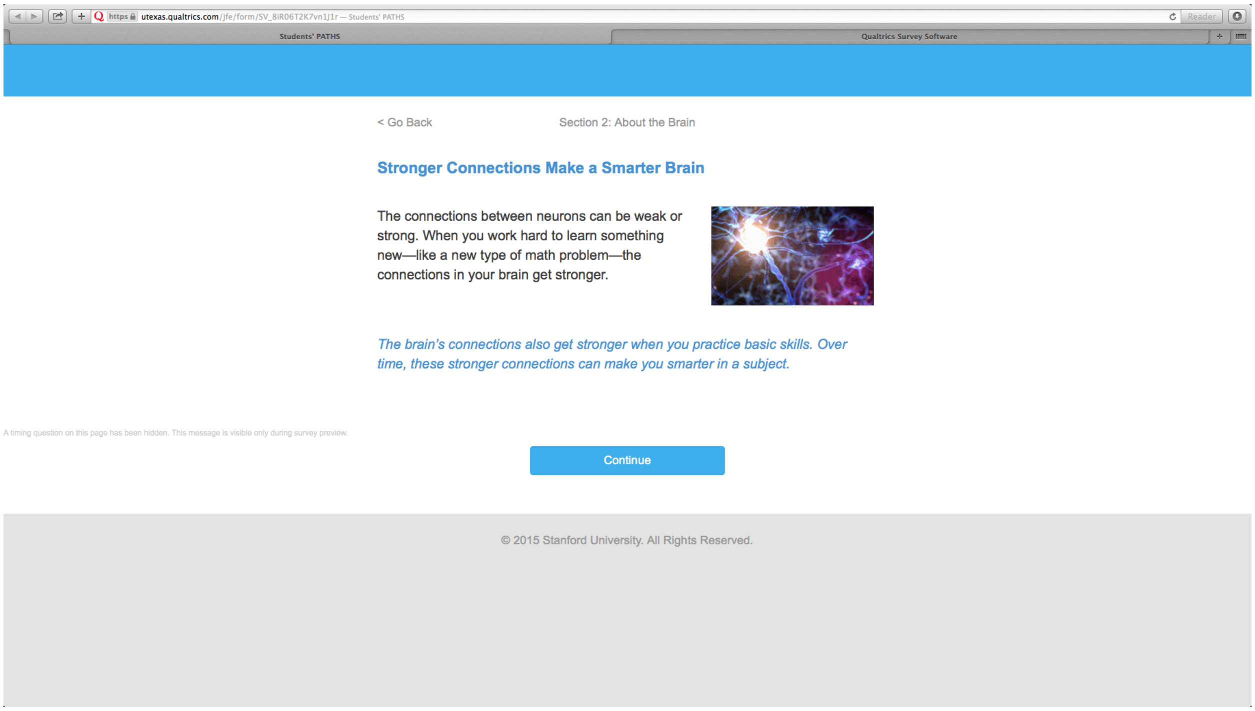

4.2.2 Treated students are presented with relevant scientific information.

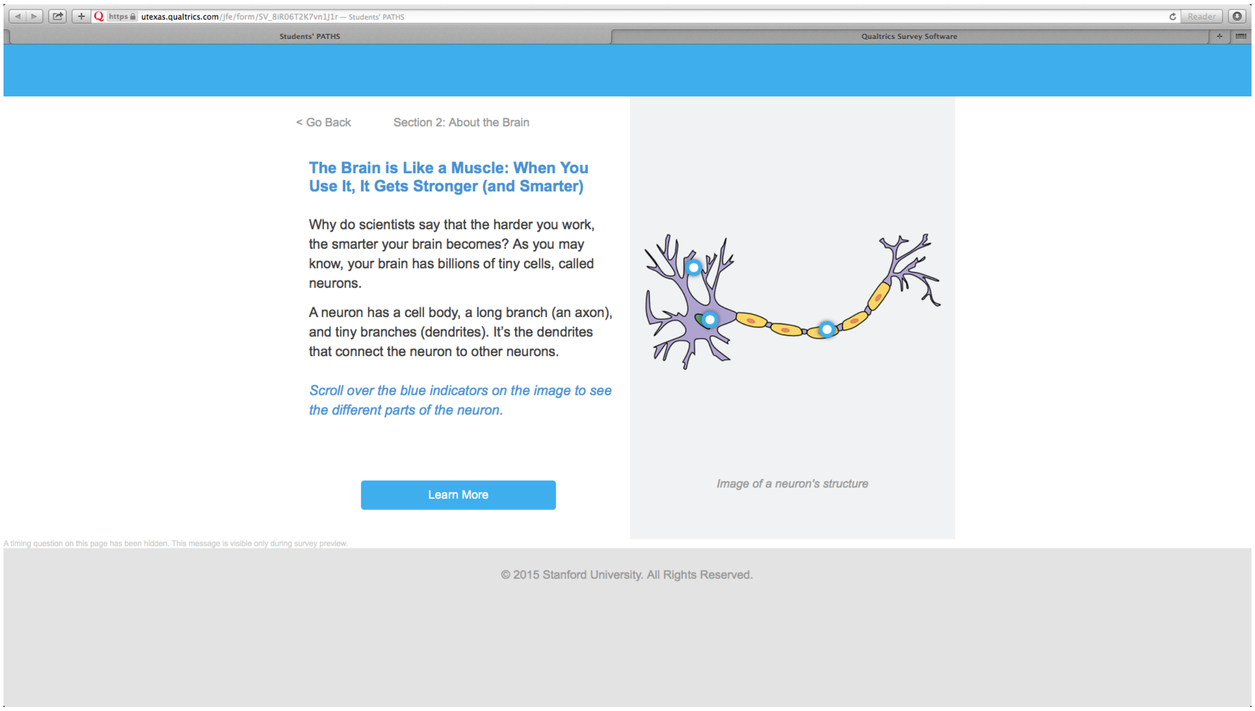

4.2.3 Treated students are asked for their help in communicating these ideas to others as a means of bringing them into the story and including them in the narrative.

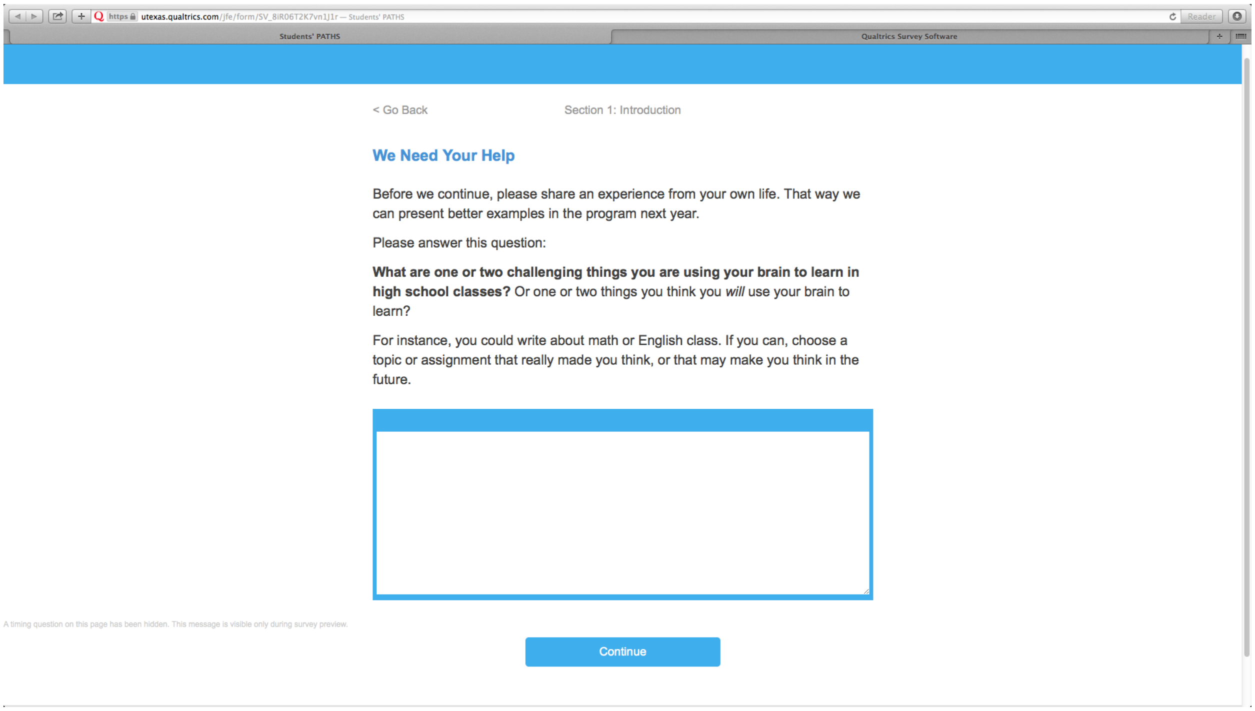

4.2.4 Treated students receive the message is that effort is not enough - you also need strategies to overcome challenges and develop your skills.

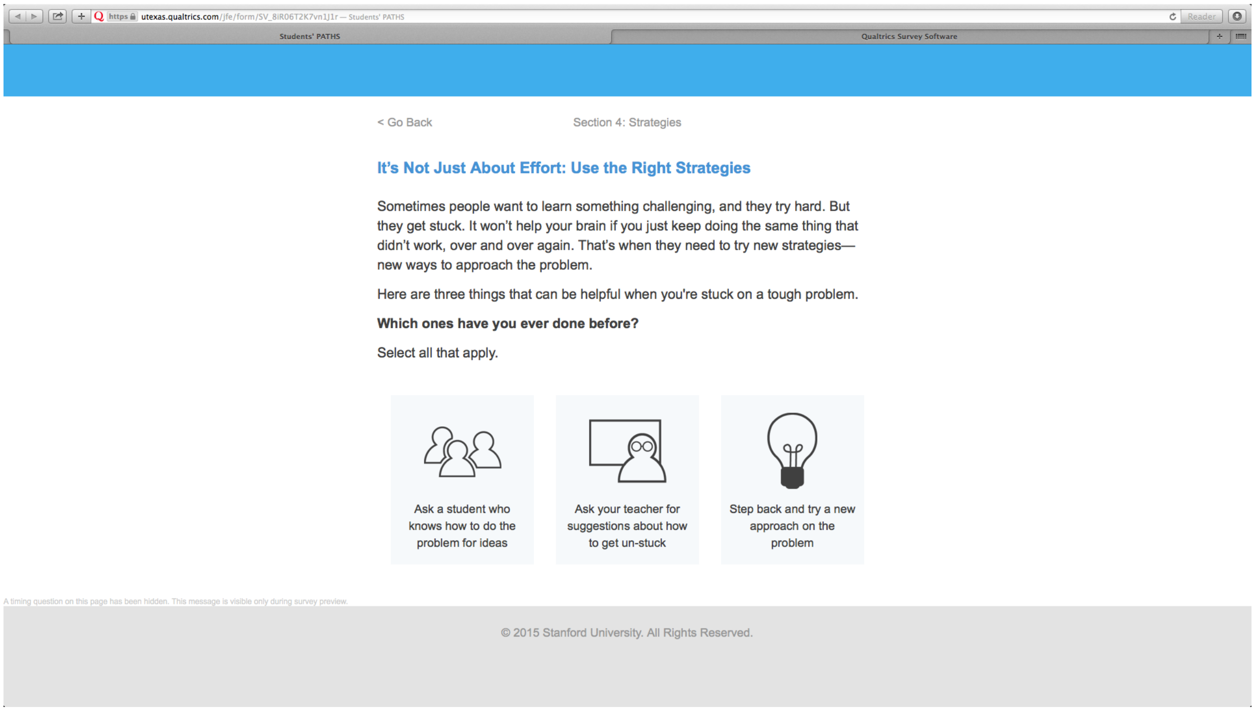

4.2.5 Materals convey that teenage years are a special time for brain growth that students can leverage to their advantage.

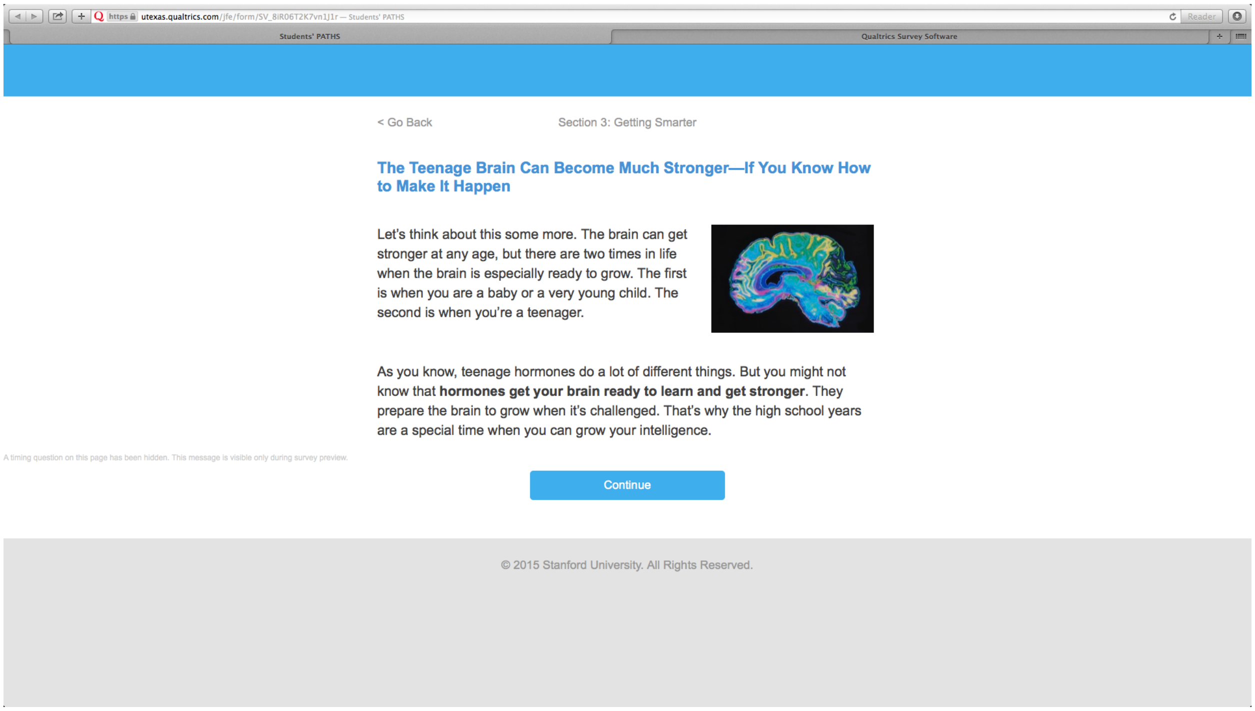
https utexas.qualtrics.com/jfe/form/SV_8iR06T2K7vn1J1r — Students' PATHS

Students' PATHS | Qualtrics Survey Software

< Go Back   Section 3: Getting Smarter

The Teenage Brain Can Become Much Stronger—If You Know How to Make It Happen

Let's think about this some more. The brain can get stronger at any age, but there are two times in life when the brain is especially ready to grow. The first is when you are a baby or a very young child. The second is when you're a teenager.

As you know, teenage hormones do a lot of different things. But you might not know that **hormones get your brain ready to learn and get stronger**. They prepare the brain to grow when it's challenged. That's why the high school years are a special time when you can grow your intelligence.

A timing question on this page has been hidden. This message is visible only during survey preview.

Continue

© 2015 Stanford University. All Rights Reserved.

4.2.6 Relating growth mindset to their own lives helps students internalize the message by customizing it, and reduces defensive reactions that might emanate from the perception that adults are telling the students what to believe.

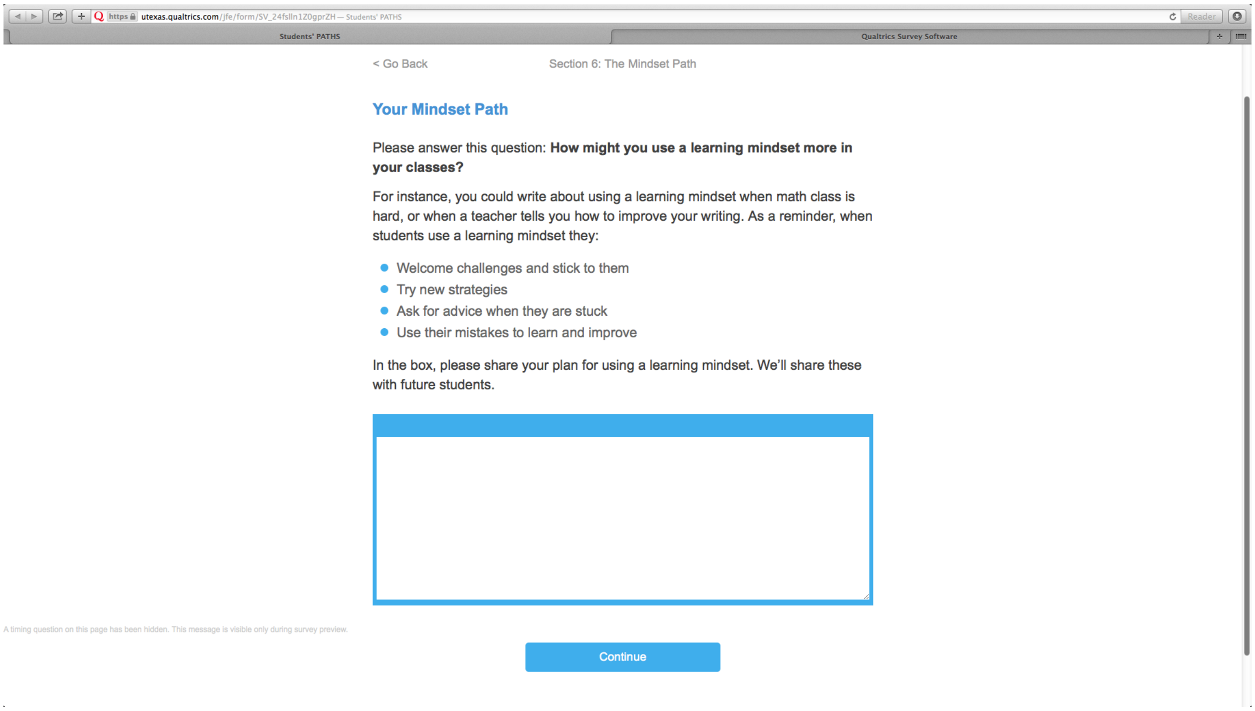
https utexas.qualtrics.com/jfe/form/SV_24fslln1Z0gprZH — Students' PATHS

Students' PATHS | Qualtrics Survey Software

< Go Back   Section 6: The Mindset Path

Your Mindset Path

Please answer this question: **How might you use a learning mindset more in your classes?**

For instance, you could write about using a learning mindset when math class is hard, or when a teacher tells you how to improve your writing. As a reminder, when students use a learning mindset they:

- Welcome challenges and stick to them
- Try new strategies
- Ask for advice when they are stuck
- Use their mistakes to learn and improve

In the box, please share your plan for using a learning mindset. We'll share these with future students.

A timing question on this page has been hidden. This message is visible only during survey preview.

Continue

4.2.7 Treated students are encouraged to see the value of applying a growth mindset to their own lives.

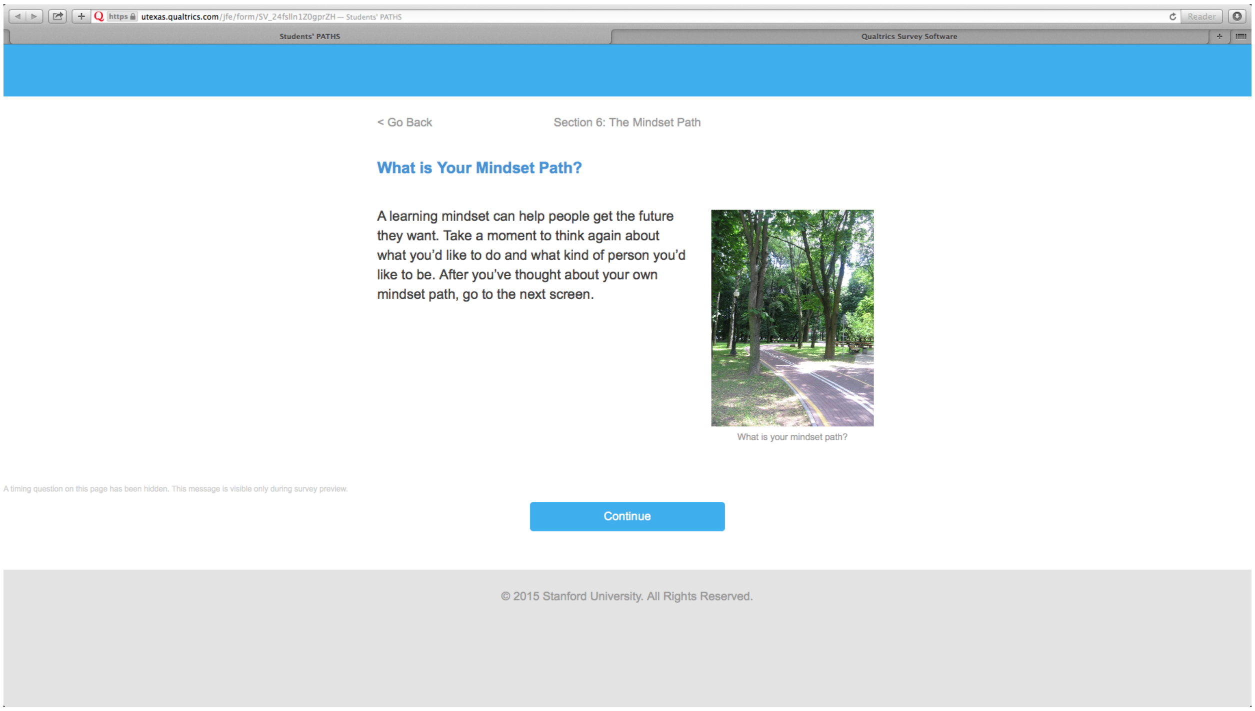

4.2.8 Student testimonials, obtained from prior study participants, help communicate that holding a growth mindset puts them in line with what other students think, and that they're not alone in their concerns about school.

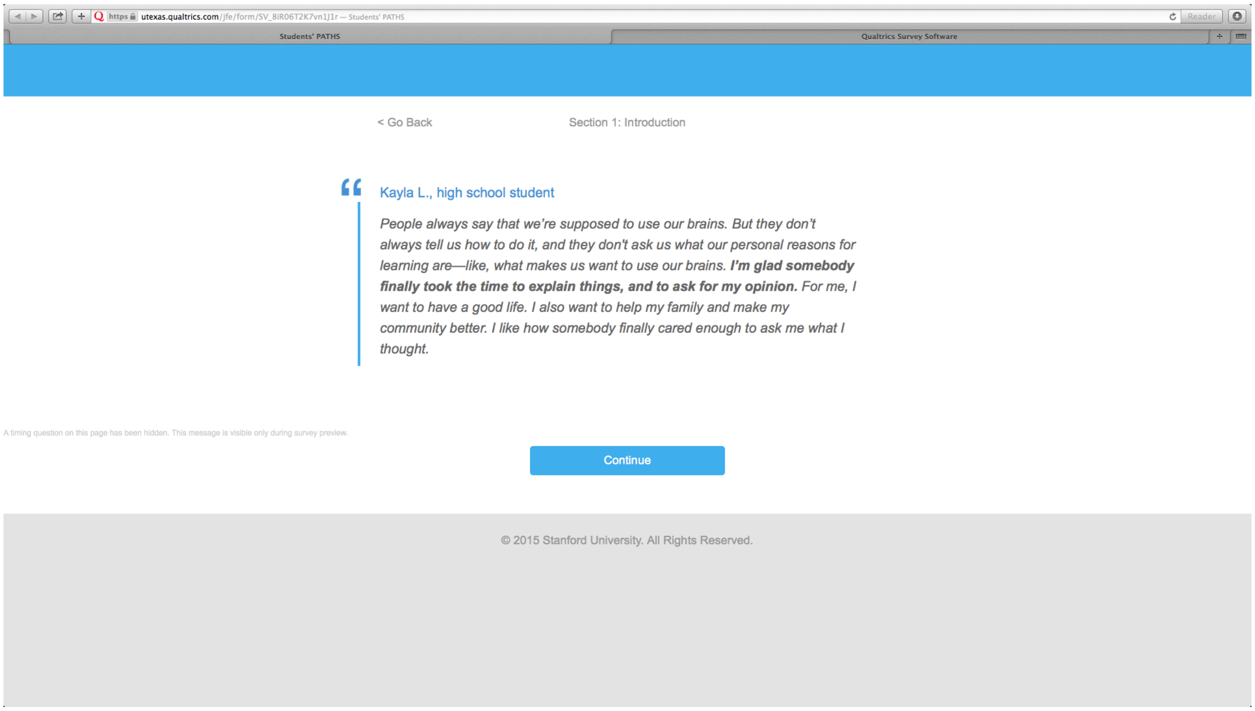

4.2.9 Treatment materials summarize evidence showing that holding a “learning mindset” (the term used for growth mindset hee) is helpful, for instance national data from Chile.

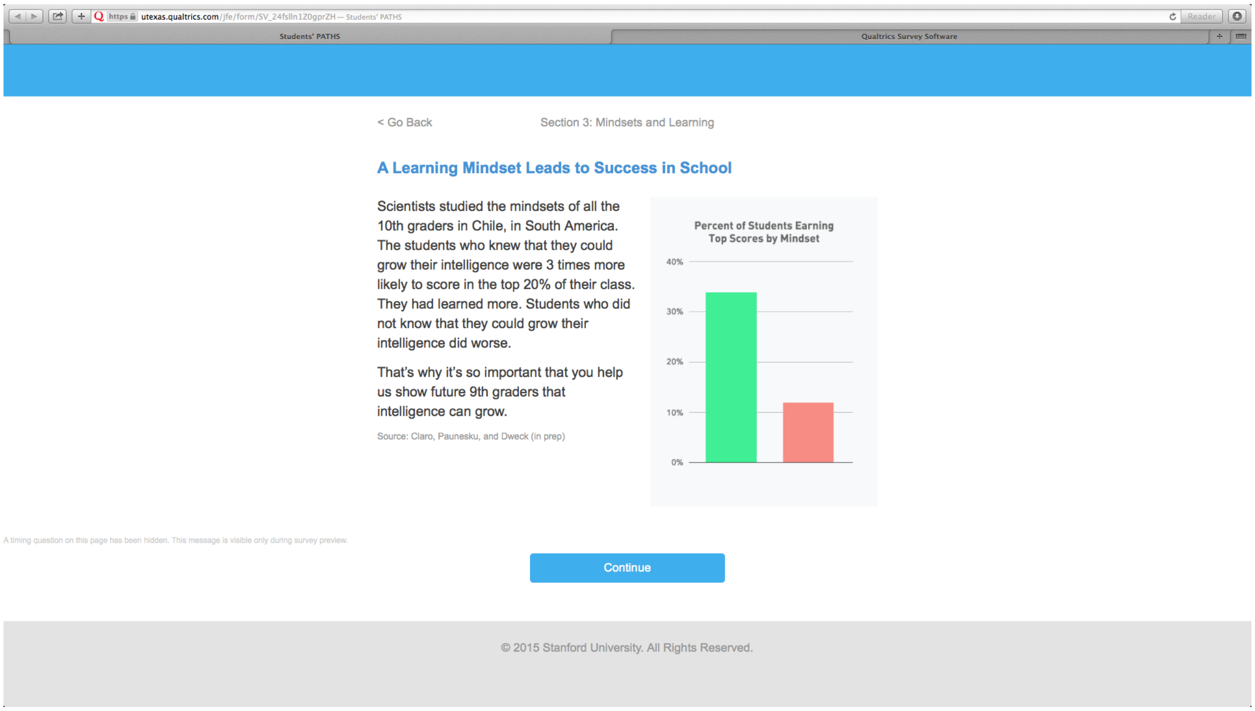

4.3 Illustrative Screenshots from the Control Condition

4.3.1 Control students are asked to help improve a lesson about the brain

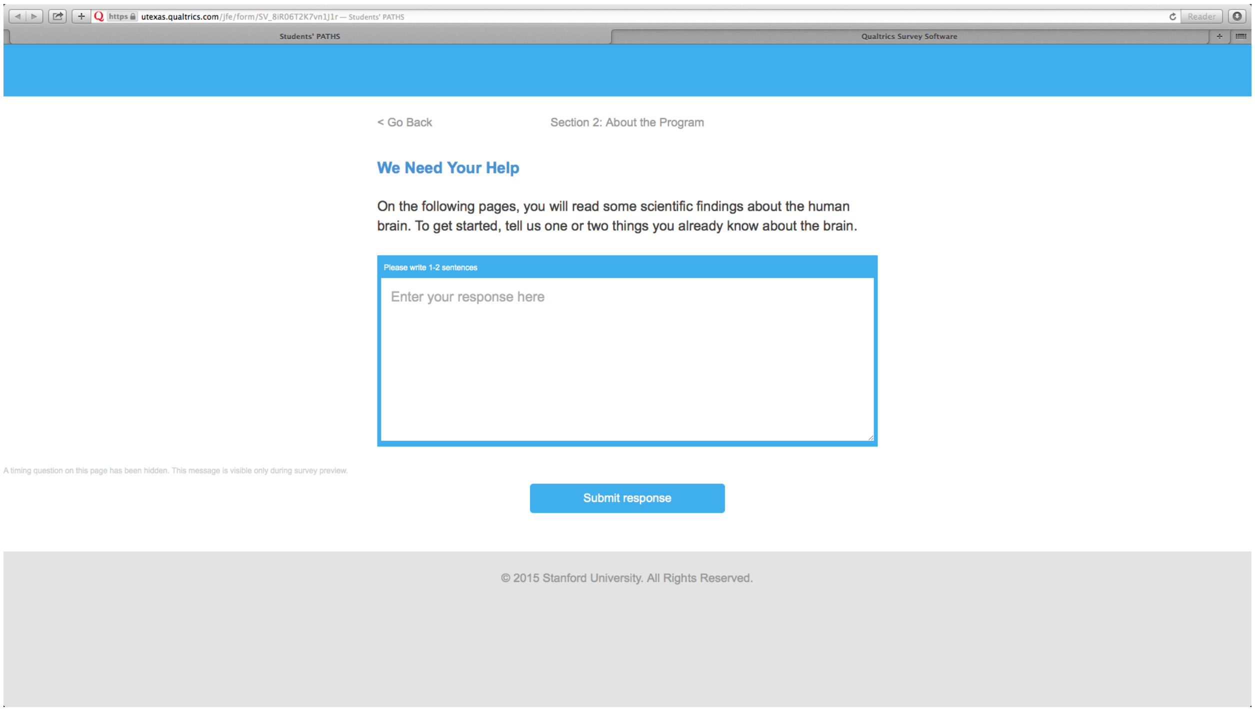

4.3.2 Control students are presented with information about physiological featues of the brain that does not include the mindset content.

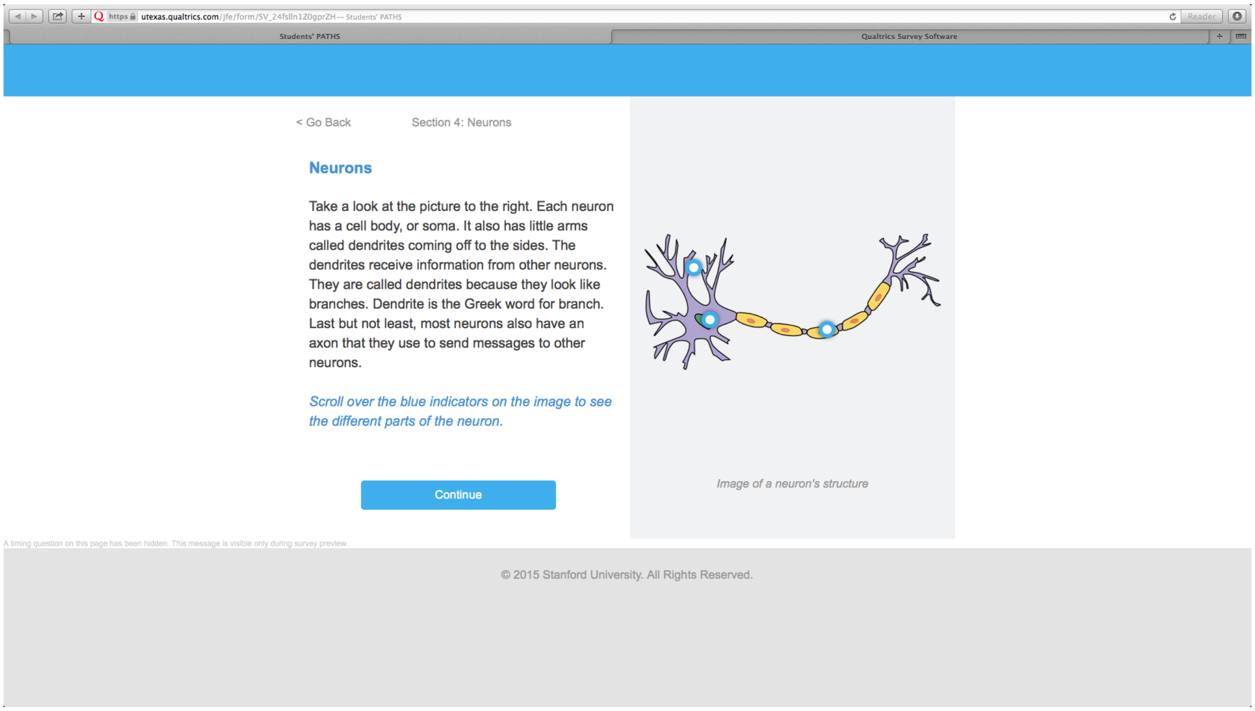

4.3.3 The control condition content includes examples of evidence about the brain.

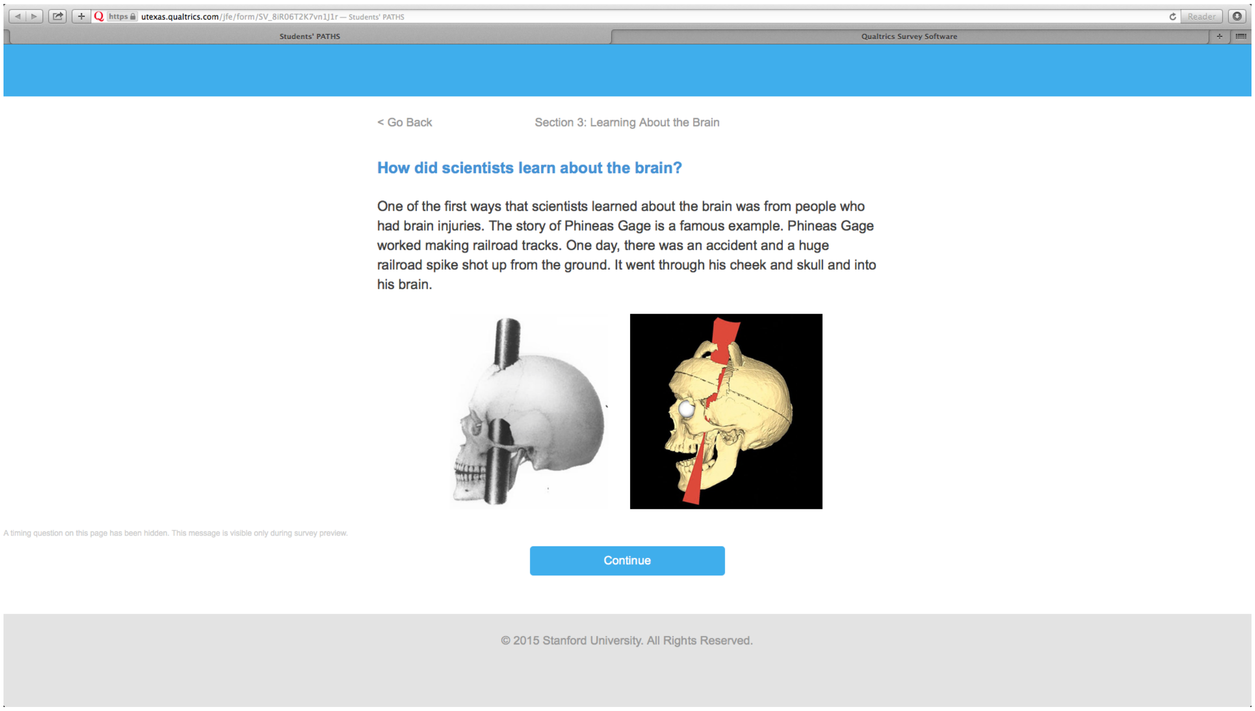

4.3.4 Control students were asked to engage with the material by responsing to short answer prompts.

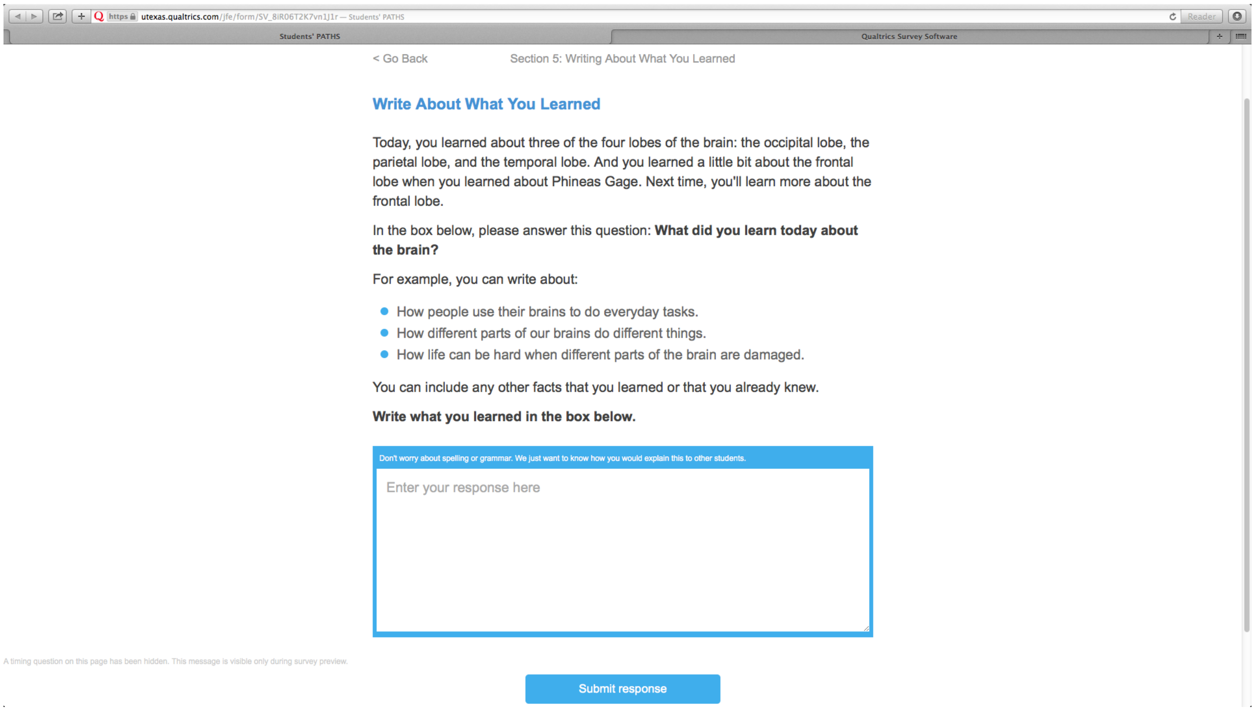

4.3.5 Control students saw student testimonials about the value of the content.

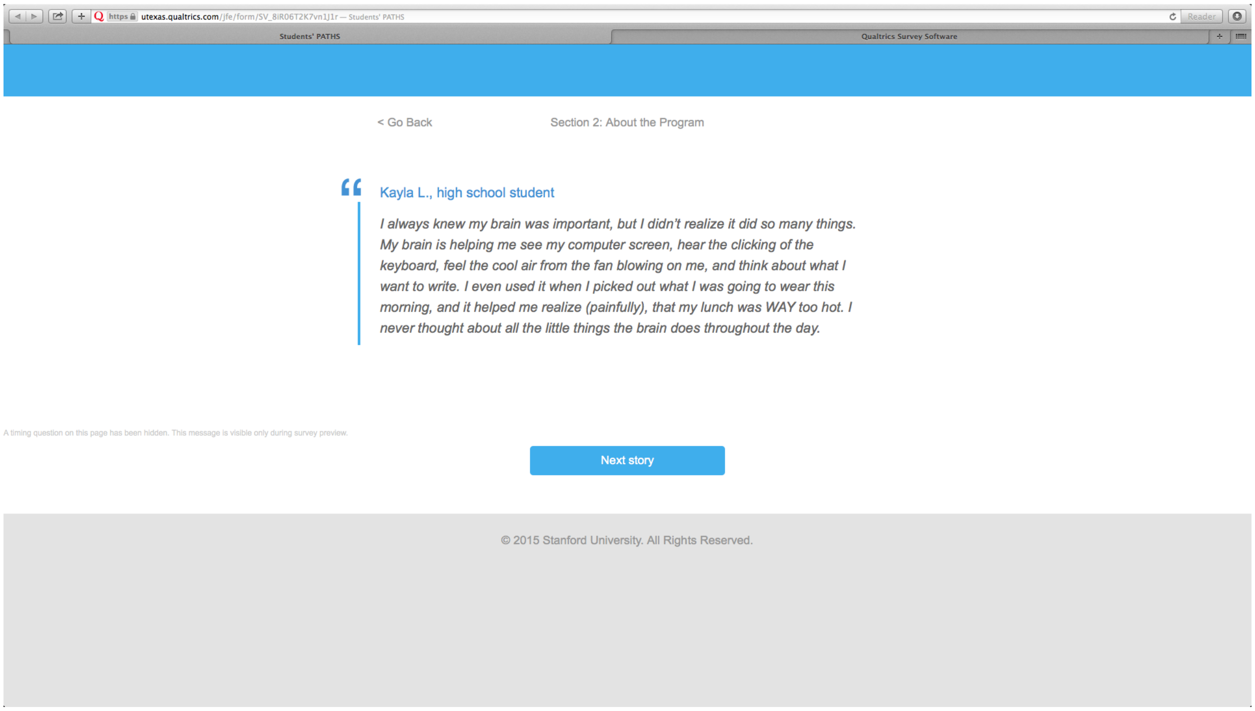

4.4 Student Responses to Three Key Treatment Open Response Prompts

In this section we summarzie students engagement with the growth mindset intervention with information about their responses to three key treatment prompts that asked students to reflect on key aspects of the mindset message. Selected text from each of the three prompts is as follows:

- A. What is a time you grew stronger connections in your brain? Think about a time you had to work really hard to get better at something in school: maybe it was a new kind of writing assignment or a math problem that seemed really hard at first. What was a time you made your brain stronger in school?
- B. This is where we really need your input. Think about new students coming to 9th grade next year. Imagine a student who is struggling in one of their classes and is feeling discouraged. Maybe the work feels too hard for them, or maybe they are having trouble staying motivated. What is the most important thing (or things) you learned today that could help them? Write a personal letter to encourage a 9th grader next year in the box below.
- C. When people have a stronger brain, they're ready to do things that matter to them. And if we want to explain this to next year's students, we need to learn what kinds of issues matter to you. Please answer this question: What issues matter most to you personally? ... Try especially to think of something where having a stronger brain might help a person like you make a difference for the issue one day.

The distribution of word counts represent high individual-level engagement with the interactive intervention materials. Responses were lower in the second session due to the students who did not see session 2 materials (570 did not participate and 60 received control condition materials due to matching errors). Among students who started session 2, engagement was comparable.

Variable	Mean	SD	Min	Q1.25.	Median.50.	Q3.75.	Max	Any	N
Prompt A	32.07	24.29	0	15	28	43	317	0.95	6700
Prompt B	49.17	41.64	0	20	41	68	388	0.93	6700
Prompt C	27.03	27.31	0	8	22	38	620	0.83	6700
Prompt C (saw any S2 treatment)	29.85	27.19	0	12	25	40	620	0.92	6070

In the following subsections we present examples of student responses for these prompts as well as more detail for the distribution of number of words written and a description of qualitative checking of the responses.

4.4.1 Free Response A

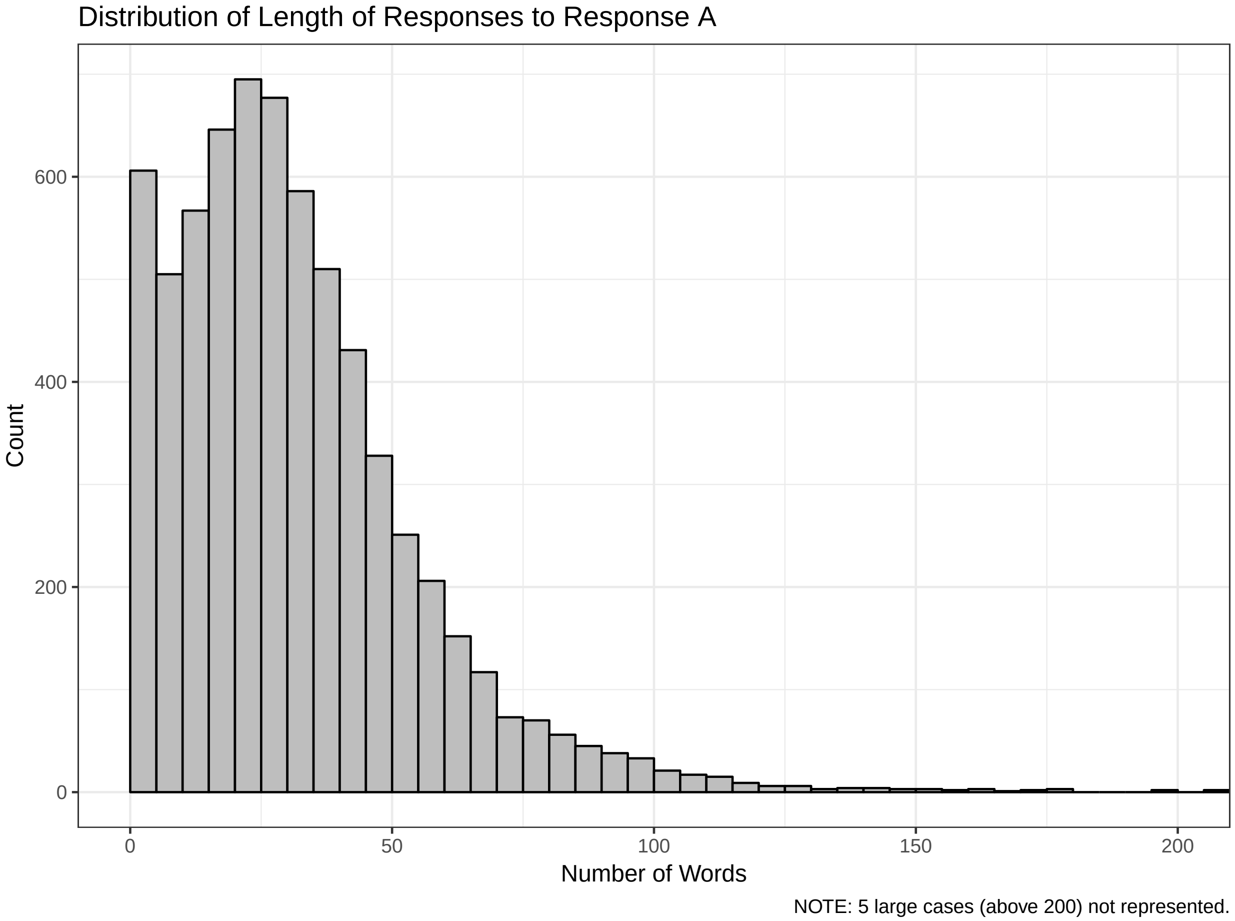

Example Responses. In response to the prompt in the treatment condition asking about a time when students had to stretch and grow their brains, students wrote:

> A time I made my brain stronger in school is every other day when I go to algebra class. It's not that it's a hard subject for me, it's just that when we first have to learn something new it's difficult at first. But then when we keep working and do practice on it, it becomes easier.
>
> There was a math unit that I really didn't understand and when we took the quiz I got a really bad grade. But I studied more and was able to retake that quiz to get a better score. My brain grew stronger during exams and finals because you need to study in order to pass and learn by doing this your brain gets stronger and smarter.
>
> In math because I couldn't really understand some assignments as much . But I started to help my mom with college alegbra so then I stared off again pumped up to do math. Ever since then I have been during real good in that class.

4.4.2 Free Response B

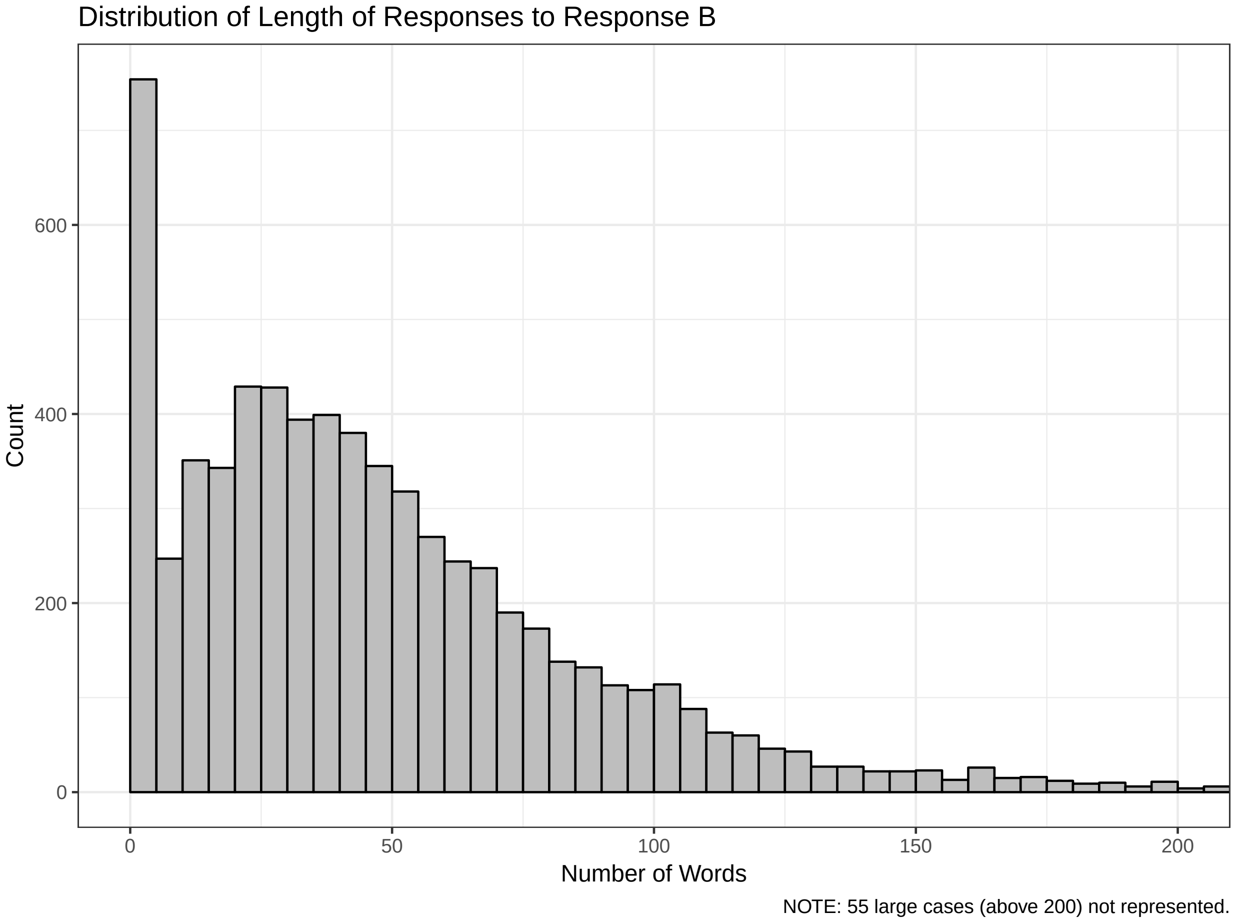

Example Responses. At the end of Session 1, students wrote what appear to be inspiring notes to future students who may be struggling in their freshman year. For instance, students wrote:

> Dear Struggling Student, Don't be afraid to ask for help because once you do you won't regret it. And just because something is hard that doesn't mean you aren't smart.

> It will be your first year in high school which means that it will be hard and you will struggle in some of your classes but that doesn't mean you have to give up and not try any different ideas. For example I thought my math was hard when I was a freshmen but after months passsing by I started to get better at math so then I started to get higher test scores on my test. So my word to you guys is to not give up and keep trying :)

> Don't be afraid or scared to learn. Just know that if you are trying your brain is getting smarter. Just because you don't know how to do it or it's too hard, just ask for help.

4.4.3 Free Response C

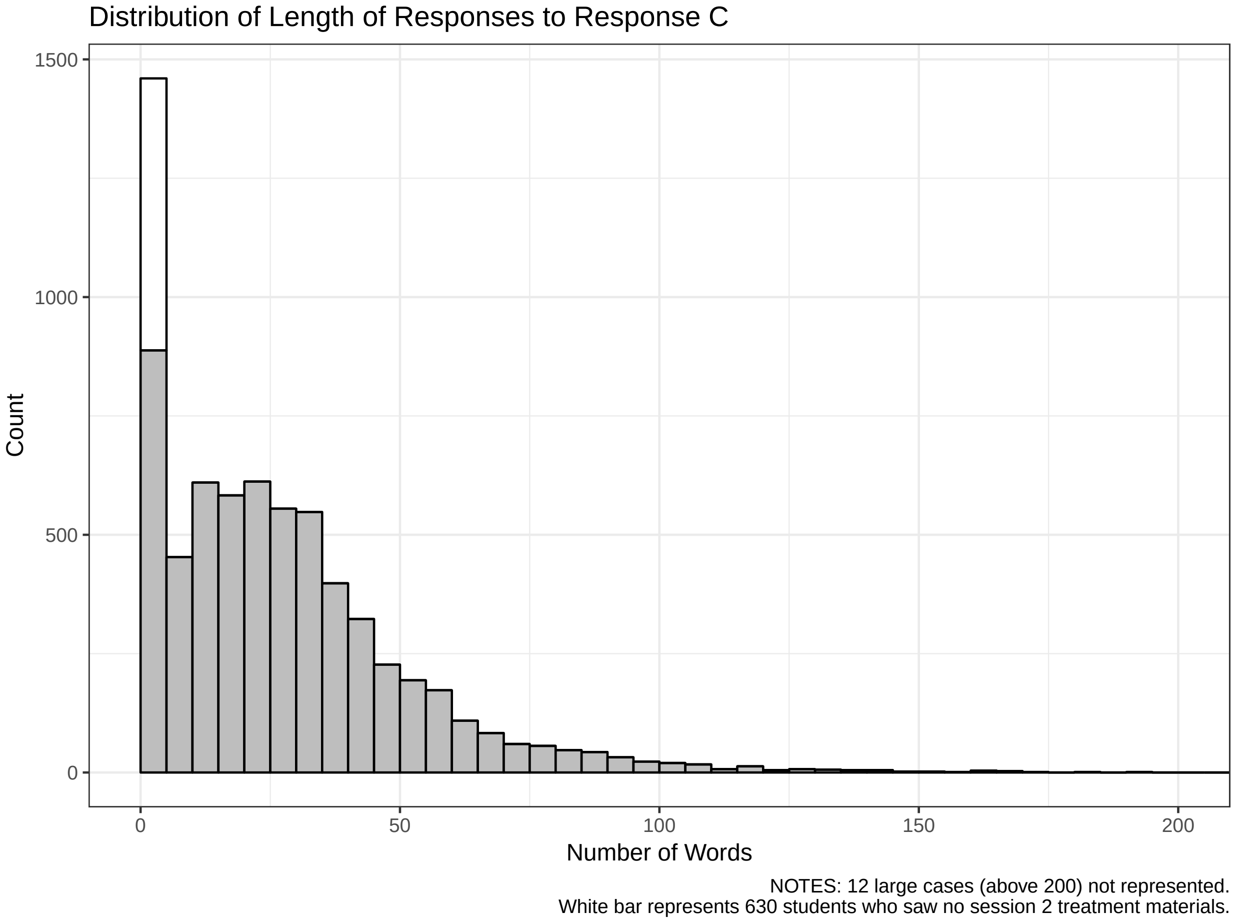

Example Responses. During Session 2, students were invited to reflect on issues that mattered most to them personally, and connect their learning to their desire to make a difference on those issues. Ninth grade students wrote passionately about a broad variety of important societal issues. Here are a few examples:

> The issues that matter most to me personally are helping people who are less fortunate than us get jobs. Society lately has been very cruel to homeless people are those who do not possess a lot of money. They tell them that they need to get a job, yet how can they get a job when they have no money to get a house, or presentable clothes?
>
> The issues that matter most to me personally would have to be dirty water in other countries. While we have nice somewhat clean water it's horrible that other countries have to drink horrible non sanitized drinking water.
>
> One issue that matters to me is the Syrian refugees. In some refugee camps, they are treated very poorly and don't get enough food and water. Also, there are some people who are stuck in Syria and can't get away, and they are stuck in a war- torn country that they can no longer call home.

4.4.4 Qualitative Assessment of Responses

As a simple measure of students' levels of engagement with the intervention content, an analyst coded whether participants wrote valid, good faith (non-gibberish) responses to free response questions A, B, and C listed above. These three were selected because they were considered critical to the intervention content (they were not comprehension checks) and they asked for a substantive, more-involved responses. The analyst drew

a random 10% of participant responses separately for each of the three questions. Blank responses (which were 3% to 8% of responses) were not sampled. According to the codebook, a valid response was an honest attempt to answer the question. An invalid response was any of these: “idk,” “I don’t know,” “nothing,” “no,” “never,” a response that stopped at the sentence starter (i.e. “dear struggling student”) or a nonsense response (e.g. random letters and numbers). In this random sample, 99% of responses were judged to be valid, honest answers, and 1% were judged to be invalid or nonsense answers. This signals high levels of engagement with the open-ended questions.

5 Implementation of the Intervention in the National Sample

5.1 Summary of Implementation and Implications

Below, we present data on the timing of the intervention sessions in the schools.

The results show that, overall, schools were quite compliant with the timing requests. Some schools, however, implemented the intervention in the spring. Moreover, students varied in how long they had between sessions.

These descriptive statistics have three implications for our study. First, although our planned analysis was to use 8th grade GPA as the prior achievement variable and 9th grade fall and spring as the outcome variable, in schools where random assignment happened in the spring, it was preferable to use fall 9th grade as the prior achievement variable. In sensitivity analyses, we examine whether or not the choice of a prior achievement term affects our conclusions.

Second, some students received the intervention very late in the year, and it was not uncommon for spring-implementation schools to deliver the second half of the intervention well into March–just two months before the school year was over. This necessarily limits the potential for the intervention to affect their grades. This problem is especially acute when considering that some schools only provided a year-end grade, not broken out by semester. Therefore some students' outcomes were already mostly determined before random assignment. Thus, the intervention effect sizes in the study are conservative relative to what might be gained with ideal timing of implementation.

Third, the relatively high rate of compliance overall testifies to the scalability of the intervention and to the effectiveness of the study procedures designed by the research team and by the independent data collection firm ICF International.

5.2 Student Survey Response Rates

Response rates, defined as the proportion of eligible students in the school who started the survey and were in the intent-to-treat sample, were high. The mean response rate across all schools for session 1 was 93.5%, and the median was 98.0%. The few cases with very low response rates came from schools that required signed consent from parents. The mean response rate across all schools for session 2 was 88.4%, and the median was 95.0%.

5.3 Timing of Intervention Sessions Within the School Year

Three quarters of schools implemented the intervention in the fall, as planned. Most schools were able to follow the request to space the two intervention sessions 3 to 4 weeks apart.

5.3.1 Semester of Implementation

Implementation	Schools	School Prop.	Students	Student Prop.
Fall	53	0.815	10360	0.773
Spring	12	0.185	3050	0.227

5.3.2 Dates of Implementation

	Fall	Spring
Proportion	0.773	0.227
Session 1 Median	2015-09-17	2016-01-26
Session 1 SD (days)	22.4	13.1
Session 1 N	10360	3050
Session 2 Median	2015-10-12	2016-02-18
Session 2 SD (days)	22.9	15.9
Session 2 N	9770	2520
Median days between Sessions	21	27
SD of days between Sessions	10.8	9.4

5.3.3 Timing of First Session

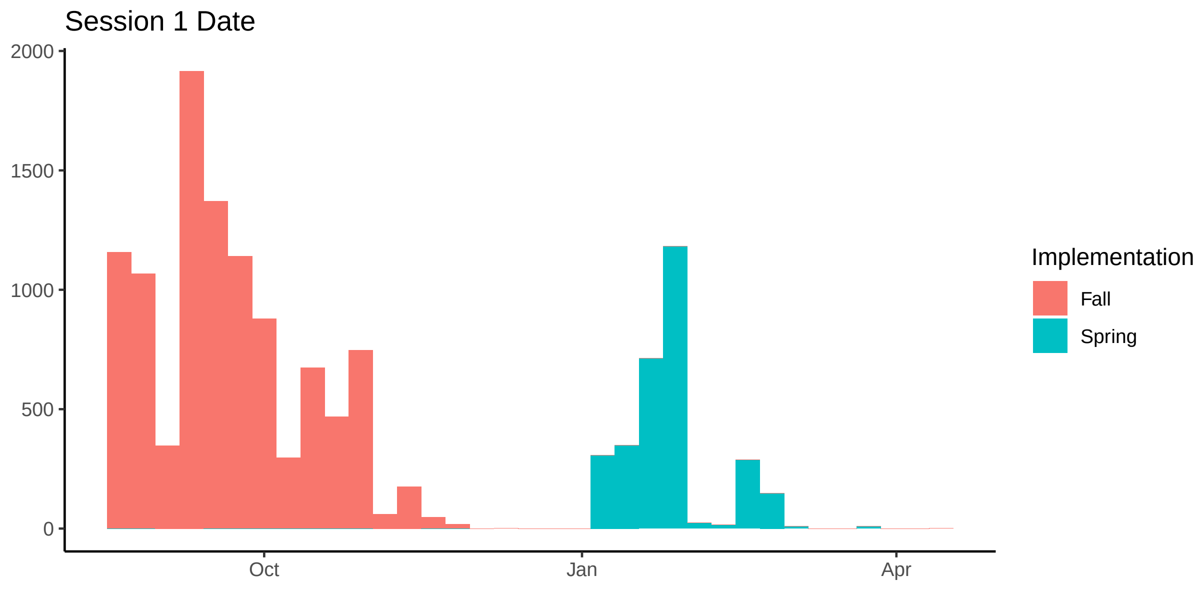

5.3.4 Spacing Between Sessions

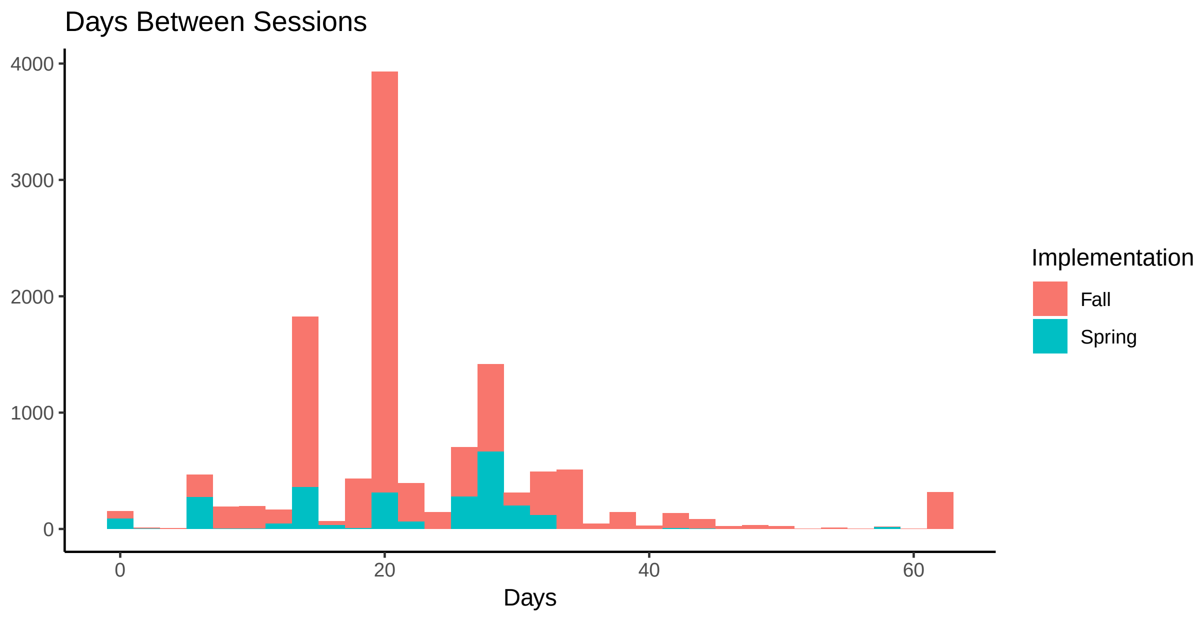

5.4 How Long Did Students Spend on the Intervention Exercises?

Each of the two study sessions took students about 25 minutes on average, for a total of 50 minutes overall. This is notable because the primary analyses are looking for effects of this 50-minute experience on grades across all core classes at the end of the school year, sometimes many months later.

The control group was shorter in session 1, corresponding to somewhat less content than in the intervention condition. The control group was a longer in session 2 because students answered extra questions about the classroom and school climate. These extra survey questions were included so that secondary data analysis of this dataset could be conducted on other topics besides intervention effects.

5.4.1 Average Time Spent on each Session, in Minutes

Session	Mean (minutes)	SD	Median
1	25.4	8.3	24.0
2	26.7	9.2	26.2

5.4.2 Distribution of Time Spent on each Session, in Minutes

Here, we report the distribution of time spent on the intervention materials for each session. This information is relevant to the scalability of the intervention, because if large proportions of students required more time for a given session than a typical class period would allow, it would make the intervention difficult to scale. However, very few students required more than the typical 40-50-minute class period, and those in the high end of the distribution are likely to be students who forgot to close their Internet browsers.

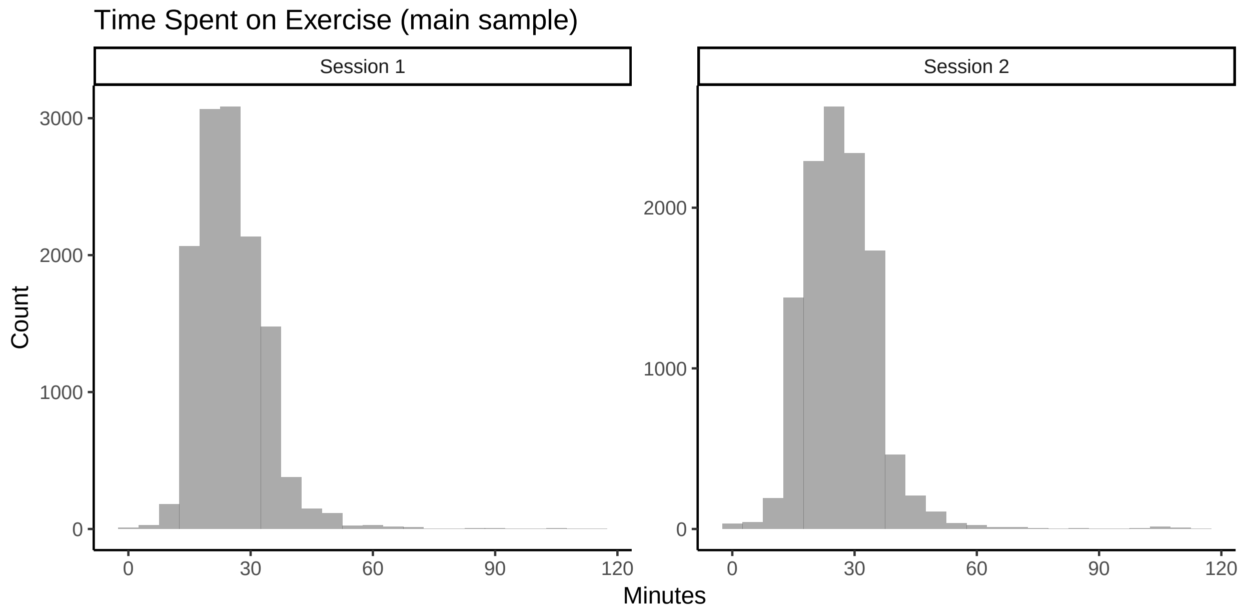

5.4.3 Time Spent by Condition, in Minutes

Variable	Trt Mean	Trt SD	Trt N	Ctl Mean	Ctl SD	Ctl N	Diff	p	ES
s1_minutes	26.412	8.682	6330	24.331	7.876	6460	2.081	0	0.264
s2_minutes	25.860	9.109	5810	27.554	9.296	5780	-1.694	0	-0.182

5.5 Session Completion

Here we present the rates at which students started and finished key aspects of each intervention session. This is informative because it shows that students, in general, showed high compliance with study procedures. We distinguish starting/finishing the overall session (defined as seeing the first and last screen of the survey) from staring/finishing the intervention material (defined as seeing the first/last screen of the intervention materials). In both Sessions 1 and 2 there were screens before and after the intervention content. Survey items preceded the first session and were included after both sessions.

Proportion of Students who Started/Finished Session 1 Sections for All Participants (N = 13410)

	Proportion
Started Session 1	1.000
Started S1 Intervention Materials	0.997
Finished S1 Intervention Materials	0.980
Finished Session 1 Survey Qs	0.869

Proportion of Students Who Started/Finished Session 2 Sections for All Participants (N = 13410)

	Proportion
Started Session 2	0.916
Started S2 Intervention Materials	0.910
Finished S2 Intervention Materials	0.855
Finished Session 2 Survey Qs	0.726

Proportion of Students Who Started/Finished Session 2 Sections for Participants who Started the Session (N = 12290)

	Proportion
Started Session 2	1.000
Started S2 Intervention Materials	0.993
Finished S2 Intervention Materials	0.933
Finished Session 2 Survey Qs	0.792

5.5.1 Completion by Experimental Group

Proportion of Students Who Completed Intervention Sectsion by Experimental Group

Status	Trt Mean	Trt N	Ctl Mean	Ctl N	Diff	p	ES
Started S1 Intervention Materials	0.997	6700	0.997	6710	0.000	1.000	0.000
Finished S1 Intervention Materials	0.974	6700	0.986	6710	-0.012	0.000	-0.103
Finished S1	0.847	6700	0.892	6710	-0.045	0.000	-0.144
Started Session 2	0.915	6700	0.918	6710	-0.004	0.458	-0.013
Started S1 Intervention Materials	0.997	6700	0.997	6710	0.000	1.000	0.000
Finished S1 Intervention Materials	0.974	6700	0.986	6710	-0.012	0.000	-0.103
Finished S1	0.847	6700	0.892	6710	-0.045	0.000	-0.144

Note: Trt = Treatment, Ctl = Control, Diff = Treatment - Control difference, p = p-value for difference, ES = Effect Size (Glass's Delta)

5.6 Implementation Fidelity

We considered the following the following pre-registered measures of school-level fidelity of implementation, ultimately combining:

1. the percentage of open-ended questions that students answered during their on-line sessions,
2. the percentage of screens that students opened (and presumably viewed) during their on-line sessions,
3. the student-level response rate,
4. the amount of distraction that students reported experiencing during their on-line sessions, and
5. the amount of distraction that students reported other students experienced during their on-line sessions

These correspond to the dimensions list in the pre-registration plan, with the final item combining two distraction items (self and others).

Distribution of Fidelity Measures across 65 Schools (All Variables are Proportions)

Variable	Mean	SD	Min	Q1.25.	Median.50.	Q3.75.	Max
1. Open-ended Responses Completed	0.950	0.035	0.810	0.938	0.959	0.975	1
2. Intervention Screens Seen	0.950	0.057	0.775	0.939	0.968	0.992	1
3. Student Response Rate	0.932	0.140	0.120	0.940	0.980	1.000	1
4. Reported Little/No Distraction for Self	0.888	0.070	0.504	0.876	0.900	0.923	1
5. Reported Most or All Students Focused	0.889	0.080	0.504	0.871	0.910	0.936	1

Fidelity is high across these measures and consistently so. This modest variation in fidelity reflects the refined design of the intervention and the ongoing efforts of the independent research firm. It also limits our ability to investigate. However, we conducted a sensitivity analyses to see if including school-level fidelity in school moderation models altered the substantive conclusions. It did not (see below).

6 Data Description

In this section we provide background on the key variables and characteristics of the experimental study.

6.1 Defining Key Variables

- `Growth Mindset Scale` = Post-intervention mindset 3-item scale; values range from 6 (most growth mindset) to 1 (most fixed mindset). The survey items are framed in terms of a fixed mindset, and so they are reverse-scored in order to obtain fixed mindset values.
- `Growth Mindset Indicator` = Post-intervention growth mindset indicator, greater than 4.0 on 3-item growth mindset scale (less than 3.0 on original fixed mindset scale)
- `Hard Problems` = Willingness to seek out challenges in math (number of hard problems selected in make-a-math-worksheet task); also referred to as “challenge-seeking” and the basis for a school mindset saturation measure
- `Self-reported Growth Mindset Norm` = School average of growth mindset self-reports noted above, estimated from all students prior to random assignment. In the pre-registration, we called this “mindset saturation (self-report operationalization).”
- `Behavioral Challenge-Seeking Norm` = School average of hard problems students chose on the “make-a-math-worksheet” task, estimated from all students in the control group who completed the Session 2 survey. In the pre-registration, we called this “mindset saturation (behavioral operationalization).”
- `GPA` = Post-intervention grades in core academic courses (mathematics, English/English Language Arts, science, social studies; omitting support courses like labs or tutorials) in 9th grade, on a 0 to 4.3 scale.
- `D/F Avg` = Core GPA in D or F Range (less than 2.0 on a 0 to 4.3 scale)

6.2 Summary of Full Sample (unweighted)

Below we summarize key pre-intervention and outcome variables in the analytic sample.

6.2.1 Descriptive Statistics for the Full Sample

Variable	Mean	SD	Min	Max	N	ICC
Female	0.490	0.500	0	1.0	13360	0.027
Maternal College	0.289	0.453	0	1.0	13410	0.313
Asian	0.038	0.191	0	1.0	13340	0.408
Black	0.112	0.315	0	1.0	13340	0.755
Hispanic	0.244	0.429	0	1.0	13340	0.622
White	0.430	0.495	0	1.0	13340	0.613
Other or Multiple Race/Ethnicity	0.176	0.381	0	1.0	13340	0.190
Pre-intervention Growth Mindset Scale	3.941	1.157	1	6.0	13390	0.045
Pre-intervention Core GPA	2.801	0.987	0	4.3	11170	0.183
Post-intervention Growth Mindset Scale	4.299	1.189	1	6.0	11980	0.034
Post-intervention Growth Mindset (Dichotomous)	0.584	0.493	0	1.0	11980	0.088
Post-intervention Core Academic GPA	2.596	1.074	0	4.3	12490	0.125
Post-intervention D/F Average	0.270	0.444	0	1.0	12490	0.307
Post-intervention Math/Science GPA	2.514	1.143	0	4.3	12080	0.106

Note: ICC = intraclass correlation coefficient at the school level, representing the proportion of variance estimated to be between schools.

6.3 Descriptive Statistics for Previously Lower-achieving Students (unweighted)

We hypothesize larger intervention effects on GPA for lower-achieving students, defined as those with achievement below the school median prior to random assignment. The steps taken to define this group are described in the pre-registered analysis plan. Below we present the descriptive statistics for this subgroup.

6.3.1 Descriptive Statistics for the Lower-achieving Sub-sample

Variable	Mean	SD	Min	Max	N	ICC
Female	0.414	0.493	0	1.00	6950	0.038
Maternal College	0.218	0.413	0	1.00	6990	0.341
Asian	0.026	0.158	0	1.00	6940	0.340
Black	0.127	0.333	0	1.00	6940	0.743
Hispanic	0.280	0.449	0	1.00	6940	0.644
White	0.367	0.482	0	1.00	6940	0.599
Other or Multiple Race/Ethnicity	0.201	0.401	0	1.00	6940	0.197
Pre-intervention Growth Mindset Scale	3.787	1.161	1	6.00	6980	0.044
Pre-intervention Core GPA	2.112	0.833	0	3.70	5590	0.413
Post-intervention Growth Mindset Scale	4.121	1.221	1	6.00	6090	0.038
Post-intervention Growth Mindset (Dichotomous)	0.518	0.500	0	1.00	6090	0.097
Post-intervention Core Academic GPA	2.010	0.962	0	4.24	6320	0.201
Post-intervention D/F Average	0.452	0.498	0	1.00	6320	0.410
Post-intervention Math/Science GPA	1.891	1.035	0	4.30	6140	0.177

Note: ICC = intraclass correlation coefficient at the school level, representing the proportion of variance estimated to be between schools.

6.4 Descriptive Statistics for Previously Higher-achieving Students (unweighted)

Higher-achieving students are defined as those above the school median prior to random assignment. The steps taken to define this group are described in the pre-registered analysis plan. Below we present the descriptive statistics for this subgroup.

6.4.1 Descriptive Statistics for the Higher-achieving Sub-sample

Variable	Mean	SD	Min	Max	N	ICC
Female	0.572	0.495	0.00	1.0	6410	0.056
Maternal College	0.366	0.482	0.00	1.0	6420	0.349
Asian	0.051	0.221	0.00	1.0	6400	0.461
Black	0.096	0.295	0.00	1.0	6400	0.798
Hispanic	0.205	0.403	0.00	1.0	6400	0.643
White	0.499	0.500	0.00	1.0	6400	0.644
Other or Multiple Race/Ethnicity	0.149	0.356	0.00	1.0	6400	0.205
Pre-intervention Growth Mindset Scale	4.108	1.129	1.00	6.0	6420	0.051
Pre-intervention Core GPA	3.492	0.550	1.02	4.3	5580	0.546
Post-intervention Growth Mindset Scale	4.483	1.125	1.00	6.0	5880	0.038
Post-intervention Growth Mindset (Dichotomous)	0.653	0.476	0.00	1.0	5880	0.110
Post-intervention Core Academic GPA	3.197	0.821	0.00	4.3	6170	0.275
Post-intervention D/F Average	0.084	0.278	0.00	1.0	6170	0.643
Post-intervention Math/Science GPA	3.157	0.858	0.00	4.3	5950	0.215

Note: ICC = intraclass correlation coefficient at the school level, representing the proportion of variance estimated to be between schools.

6.5 GPA Distributions for Previously Lower- and Higher-Achieving Students

Because the pre-specified definition of previously lower-achieving students is relative to their high school, there is some overlap in the distribution of prior achievement for these groups on the absolute grade point scale (which itself may not be fully comparable across schools). The distributions highlight that some students with below-median prior achievement have objectively high grades (e.g., an A-). Intervention impacts on GPA may be lower for this group. They may face fewer immediate academic challenges for which a growth mindest is thought to be most beneficial, and practically, there may be ceiling effects for achievement measures. As a result, we conduct sensitivity analyses with a more restrictive “lower-achieving” subgroup.

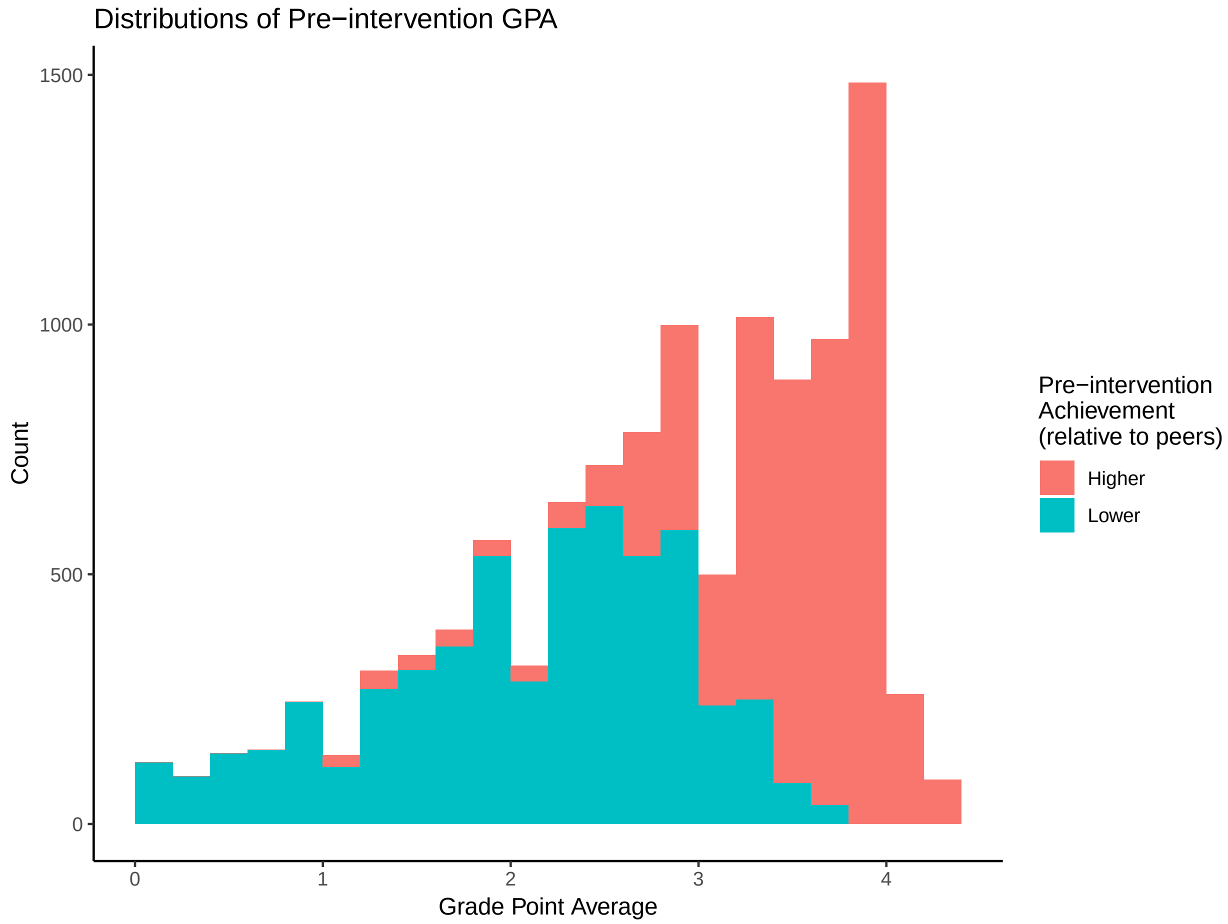

6.6 Additional Information on Components of the Lower-achieving Designation

Our pre-registered lower-achieving group indicator is based on pre-intervention GPA when available, supplemented with self-reported expectations of success or standardized test scores. Our pre-registered plan states:

> Previously low-performing students are defined as students who were earning grades lower (or equal) to 50 percent of his or her 9th grade school peers, prior to random assignment. At an operational level, this is a student whose pre-random assignment GPA is at or below the 50th percentile of his/her 9th grade peers.

In the case of missing prior GPA data, we specificed: "[W]e will impute prior achievement values using their 8th grade test scores and self-reports of expectations for success in the coming year (cf. Hulleman & Harackeiwicz, 2009)."

In this subsection, we report correlations among prior grades, expectations for success, and standardized achievement. These positive associations provide empirical support for our a priori decision to use these variables for imputation, and they show that expeations for success are more closely related to academic grades than standardized test scores.

Correlations Among Pre-Intervention Characteristics among All Students

Variable Pair	Correlation	p	N
GPA-Expectations for Success	0.351	<.001	11162
GPA-Standardized Test Score	0.262	<.001	6057
Expectations-Test Score	0.129	<.001	6730

Among the control group, we see that both expectations for success and standardized test scores are predictive of 9th grade GPA above even when controlling for prior GPA. Moreover, expectations are a stronger predictor of future grades (based on standardized coefficients) than standard achievement.

Estimated Coefficents from a Linear Regression Model of Post-intervention GPA among Control Group

Parameter	Std Estimate	Std SE	z	p	min95	max95
Expectations for Success	0.334	0.016	20.96	0	0.303	0.365
Standardized Test Score	0.196	0.016	12.31	0	0.165	0.228

6.7 Experimental Balance on Pre-treatment Characteristics

Random assignment was effective at producing balance between groups in terms of characteristics measured prior to random assignment.

6.7.1 Pre-intervention Characteristics by Experimental Condition

Variable	Trt Mean	Trt SD	Trt N	Ctl Mean	Ctl SD	Ctl N	Diff	p	ES
Female	0.492	NA	6670	0.488	NA	6690	0.004	0.693	0.007
Maternal College	0.285	NA	6700	0.293	NA	6710	-0.009	0.274	-0.019
Black	0.114	NA	6650	0.110	NA	6690	0.004	0.433	0.014
Asian	0.037	NA	6650	0.039	NA	6690	-0.002	0.542	-0.011
Hispanic	0.246	NA	6650	0.241	NA	6690	0.005	0.519	0.012
White	0.432	NA	6650	0.429	NA	6690	0.003	0.771	0.005
Fixed Mindset Scale	3.052	1.150	6690	3.066	1.164	6700	-0.014	0.491	-0.012
GPA	2.783	0.993	5580	2.819	0.980	5590	-0.036	0.051	-0.037

Note: Trt = Treatment, Ctl = Control, Diff = Treatment - Control difference, p = p-value for difference, ES = Effect Size (Glass's Delta)

6.8 Rates of Attrition

The intervention and control groups did not differ in terms of the proportion of students who were missing data for Grade Point Average or the outcome variables measured in session 2.

6.8.1 Attrition for Each Outcome by Experimental Condition

Outcome	Trt Attrition	Trt N	Ctl Attrition	Ctl N	p
Core GPA	0.072	6700	0.066	6710	0.212
Fixed Mindset Scale	0.110	6700	0.104	6710	0.302
Growth Mindset Indicator	0.110	6700	0.104	6710	0.302
Number of Hard Problems Selected	0.133	6700	0.133	6710	0.963
Hypothetical Challenge-seeking	0.119	6700	0.111	6710	0.168

Note: Trt = Treatment, Ctl = Control

6.9 Differential Characteristics of Students who Attritted Versus Those Who Did Not

Students missing data for the GPA outcome were more likely to be male, not have a mother with a college degree, higher fixed mindset, and lower pre-treatmenbt GPA.

6.9.1 Pre-intervention Characteristics of Students Missing the GPA Outcome

Variable	Attr Mean	Attr SD	Attr N	Sample Mean	Sample SD	Sample N	p	ES
Female	0.450	NA	900	0.493	NA	12460	0.014	-0.086
Maternal College	0.212	NA	920	0.295	NA	12490	0.000	-0.182
Black	0.162	NA	900	0.108	NA	12450	0.000	0.171
Asian	0.019	NA	900	0.039	NA	12450	0.003	-0.105
Hispanic	0.279	NA	900	0.241	NA	12450	0.013	0.088
White	0.341	NA	900	0.437	NA	12450	0.000	-0.192
Fixed Mindset Scale	3.271	1.189	920	3.043	1.153	12470	0.000	0.197
GPA	1.916	1.096	290	2.825	0.973	10880	0.000	-0.934

Note: Attr = Attriter, Trt = Treatment, Ctl = Control, SD = Standard Deviation, p = p-value for difference, ES = Effect Size (Glass's Delta)

6.10 Balance for Students with Outcome Information

Among students who were not missing data, the final sample was nevertheless balanced between conditions on pre-intervention characteristics.

6.10.1 Experimental Balance Among Students with GPA Outcome

Variable	Trt Mean	Trt SD	Trt N	Ctl Mean	Ctl SD	Ctl N	Diff	p	ES
Female	0.494	NA	6210	0.491	NA	6260	0.003	0.739	0.006
Maternal College	0.292	NA	6220	0.297	NA	6270	-0.005	0.550	-0.011
Black	0.111	NA	6190	0.106	NA	6260	0.005	0.381	0.016
Asian	0.038	NA	6190	0.041	NA	6260	-0.003	0.479	-0.013
Hispanic	0.243	NA	6190	0.239	NA	6260	0.005	0.552	0.011
White	0.437	NA	6190	0.436	NA	6260	0.002	0.875	0.003
Fixed Mindset Scale	3.034	1.145	6210	3.052	1.161	6260	-0.018	0.385	-0.015
GPA	2.810	0.978	5420	2.840	0.968	5450	-0.031	0.098	-0.032

Note: Trt = Treatment, Ctl = Control, SD = Standard Deviation, Diff = Treatment - Control difference, p = p-value for difference, ES = Effect Size (Glass's Delta)

6.10.2 Experimental Balance Among Students with Session 2 Challenge Worksheet Outcome

Variable	Trt Mean	Trt SD	Trt N	Ctl Mean	Ctl SD	Ctl N	Diff	p	ES
Female	0.491	NA	5790	0.494	NA	5800	-0.003	0.737	-0.007
Maternal College	0.297	NA	5810	0.303	NA	5820	-0.005	0.550	-0.011
Black	0.096	NA	5770	0.096	NA	5810	-0.001	0.929	-0.002
Asian	0.038	NA	5770	0.041	NA	5810	-0.003	0.515	-0.013
Hispanic	0.238	NA	5770	0.231	NA	5810	0.007	0.420	0.015
White	0.456	NA	5770	0.452	NA	5810	0.004	0.668	0.008
Fixed Mindset Scale	3.032	1.146	5800	3.054	1.162	5810	-0.021	0.320	-0.018
GPA	2.862	0.947	4820	2.886	0.939	4840	-0.024	0.215	-0.025

Note: Trt = Treatment, Ctl = Control, SD = Standard Deviation, Diff = Treatment - Control difference, p = p-value for difference, ES = Effect Size (Glass's Delta)

6.11 Sample Calculations for CONSORT Report

6.11.1 Enrollment

Participants were identified for participation by the third party research firm (ICF International) in consultation with school officials by virtue of grade membership and enrollment in targetted classes.

6.11.2 Students Enrolled in the Experimental Study

Considered	Parental Refusal	Intention to Treat (Randomized)
13490	70	13420

6.11.3 Allocation

Students were randomized at the start of the computerized activity. Students received the allocated intervention for session 1. Some students were absent and received no session 2 materials. Other students incorrectly inputted their names at session 2, and they were always given the control group materials.

All analyses are Intention-to-Treat, regardless of whether students saw the session 2 materials.

6.11.4 Students Allocated to Experimental Groups

treatment	Total	As Allocated, Both Sessions	Absent	Non-allocated Session 2 Materials
0	6720	6160	550	10
1	6700	6070	570	60

6.11.5 Follow-up

There are two primary reasons why participants were lost to follow-up for the primary analyses of GPA outcomes. First, one school did not provide administrative records. Second, some students' GPAs could not be matched with the administrative data, usually because their names or student IDs could not be matched, or because schools no longer had their records by the end of the year. We cannot discern every reason for non-matching records. However as noted above the characteristics of students who were missing the grades data were not differential by condition in terms of baseline characteristics.

6.11.6 Students with Follow-up Data

treatment	Intention to Treat	Grades not Available	Analytic Sample
0	6720	450	6270
1	6700	480	6220

7 Pre-Registered Intervention Impacts on Academic Grade Point Average (GPA)

We pre-specified four core questions about the impacts of the growth mindset intervention on core academic GPA. These questions build to the primary research question, RQ4, which is the cross-site heterogeneity in the treatment effect effect among lower-achieving students. In the sections that follow, we present the analysis methods that we used to answer the four questions.

7.1 Average Intervention Effects for All Students (Pre-registered RQ1)

Here we explain how we answered the first reserach question, which was:

1. What is the average treatment effect (ATE) of a Growth Mindset (GM) intervention on the GPA of 9th grade students in regular U.S. public high schools? (Note that the pre-registration did not predict a significant main effect, but instead only predicted a significant effect for RQ2).

Following the pre-registration plan, the analytic model for RQ1 was:

$$Y_i = \alpha + \beta_i(T_i) + \gamma(P_i) + \sum_{k=1}^{K} \theta_k(X_{ki}) + \sum_{j=1}^{J} \rho_j(S_{ji}) + e_i$$

Where:

- Y_i is the outcome for student i (GPA)
- T_i is an indicator for experimental group (1 if treatment, 0 if control)
- P_i is the prior achievement for student i, z-scored within schools
- X_{ki} is school-mean-centered baseline covariate k for student i
- S_{ji} is an indicator variable indicating that student i attends school j

Also following the pre-analysis plan, we estimated parameters using person-level weights and cluster-robust standard errors, clustered at the level of primary sampling unit (typically pairs of schools). Given the survey design, the primary sampling unit is more appropriate than the school level (which we originally indicated in our pre-registration).

The Stata code used to estimate parameters of this model is as follows:

```
svyset psu [pw = g9wt], strata(Stratum)

svy, subpop(if analysis_flag == 1) : reg gpa_post_avg treatment pregpa_imputed_smc pregpa_missing_smc pretest_imputed pretest_missing s1_exp_suc_1_imputed s1_exp_suc_1_missing pre_gpa_self_sch_centered pre_gpa_self_dummy_sch_centered gender_sch_centered asian_sch_centered black_sch_centered hisp_sch_centered native_sch_centered mideast_sch_centered pacisl_sch_centered white_sch_centered pared_1_sch_centered pared_2_sch_centered pared_3_sch_centered pared_4_sch_centered pared_5_sch_centered pared_6_sch_centered pared_7_sch_centered pared_8_sch_centered ell_sch_centered sped_sch_centered gt_sch_centered firstyearfreshman_sch_centered lunch_sch_centered i.school_id
```

7.2 Conditional Average Intervention Effect for Previously Lower-achieving Students (Pre-registered RQ 2)

Here we explain how we answered the second research question, which was:

2. What is the conditional average treatment effect (CATE) of a GM intervention on the GPA of 9th grade previously lower-performing students in regular U.S. public high schools?

The models for RQ2 are similar to RQ1, except that analyses are restricted to the subgroup of lower-achieveing students.

7.3 Robustness and Sensitivity Analyses for RQ1 and RQ2

In addition to the pre-specified analyses, we considered the sensitivity of results to several alternative specifications, listed below. Note that pre-specified options are highlighted in bold. The results of these analyses appear in the Extended Data.

Survey weights:

1. **Grade 9 weights** [pre-specified] = records weighted by weighted based on sampling design and non-response (including missing grade 9 GPA outcomes); weights calculated by survey firm
2. Grade 9 weights trimmed = records weighted by a modified version of the Grade 9 weights, with weights top coded to the 3rd highest value within school achievement groups
3. Design weights = records weighted by the inverse of intervention selection for the school, given the sampling design
4. No weights = all individual records assigned a constant weight, maintaining clustering corrections for strata and primary sampling unit; expected to yield *conservative* estimates because the study over-sampled schools expected to show small or null effects

Alternate GPA Outcomes:

1. **Grade 9 post core** [pre-specified] = GPA in core academic courses (Mathematics, English Lanugage Arts, Science, Social Studies) from the intervention term to the end of the year; support courses not included
2. Grade 9 post academic = GPA in all academic courses (including support courses and Foreign Lanugage, etc.) from the intervention term to the end of the year; expected to yield *conservative* estimates
3. Grade 9 post English/math/science = core GPA without social studies (English, Mathematics, and Science courses) from the intervention term to the end of the year; this variable replicates the pilot study's outcome, as explained below.
4. Grade 9 average core = GPA in core academic courses for all of 9th grade; expected to yield *conservative* estimates because includes some pre-intervention information

Prior Low Performance Definition:

1. **Lower-achiever** [pre-specified] = below school-level median pre-intervention GPA relative to high school peers (using prior expecations or standardized tests when grades are unavailable)
2. Restricted Lower-achiever = a more restrictive subset that starts with the pre-specified relative definition and omits students above absolute thresholds (GPA above 3.3 or highest self-reported expectations of academic success).

Alternative Covariates:

1. None; expected to yield *conservative* estimates
2. School fixed effects = indicators for each school; expected to yield *conservative* estimates
3. Prior achievement = school fixed effects and prior GPA; expected to yield *conservative* estimates
4. **Full** [pre-specified] = school fixed effects, prior GPA, and demographic/academic characteristics (*)

(*) Covariates:

- standardized achievement, imputed zero if missing
- indicator for missing standardized achievement
- pre-intervention expectancy for school success, imputed zero if missing
- indicator for missing expectancy
- prior gpa self-report, imputed zero if missing
- indicator for missing prior gpa self-report
- gender

- race/ethnicity indicators (Asian, Black, Hispanic, Native American, Multi-racial, White)
- parental education categorical indicators (1-8)
- English Language Learner classification
- Special Education classification
- First year freshman indicator
- Free/reduced lunch indicator

Alternative prior achievement specification:

1. **Most recent pre-intervention GPA** [pre-specified] = Most recent pre-intervention GPA: grade 8 if intervention conducted in semester 1; grade 9 semester 1 if intervention conducted in semester 2
2. Grade 8 = Grade 8 GPA for all students; expected to yield *conservative* estimates

Alternate School Samples

1. **All Schools** [pre-specified] = all 65 schools
2. Fall Implementers = 54 schools that implemented the intervention in the fall semester of 9th grade
3. No Prior Grads in Outcome = 61 schools, omitting only 4 spring-implementing school that only provided year-long GPA, meaning that the majority of the GPA outcome is based on pre-intervention performance

7.4 School Heterogeneity (Pre-registered RQ3)

Here we explain how we answered the third research question, which was:

3. How much does the CATE of a GM intervention (on the GPA of 9th grade previously lower-performing students) vary across U.S. public high schools?

Following the pre-registration plan, the analytic model for RQ3 was a multilevel model:

Level one (students):

$$Y_i = \alpha_j + \beta_j(T_{ij}) + \gamma(P_{ij}) + \sum_{k=1}^{K} \theta_k(X_{kij}) + +e_{ij}$$

Level two (schools):

$$\beta_j = \beta + r_j$$

with:

$$e_{ij}\ N(0, \sigma_T^2)$$

$$r_j\ N(0, \tau_T^2)$$

Where:

- Y_{ij} is the outcome for student i in school j (GPA)
- α_j is a school-specific intercept
- T_{ij} is an indicator for experimental group (1 if treatment, 0 if control)
- P_{ij} is the prior achievement for student i in school j, z-scored within schools
- X_{kij} is school-mean-centered baseline covariate k for student i from school j

The Stata code used to estimate parameters of this model is as follows:

> mixed gpa_post_avg treatment pregpa_imputed_smc pregpa_missing_smc pretest_imputed_smc pretest_missing_smc s1_exp_suc_1_imputed_smc s1_exp_suc_1_missing_smc pre_gpa_self_smc pre_gpa_self_dummy_smc gender_smc asian_smc black_smc hisp_smc native_smc mideast_smc pacisl_smc white_smc pared_1_smc pared_2_smc pared_3_smc pared_4_smc pared_5_smc pared_6_smc pared_7_smc pared_8_smc ell_smc sped_smc gt_smc firstyear-freshman_smc lunch_smc i.school_id if analysis_flag == 1 || schoolid: treatment, nocons , reml

The parameter of interest is tau, the standard deviation of intervention impacts across schools. Multi-level model heterogeneity analyses are estimated with restricted maximum likelihood (REML), because this is the ideal method for estimating the random effect, but this particular model cannot account for sampling weights because REML does not function with weights. In the unweighted sample, the estimated intervention effect for lower-achieving students on post-intervention GPA in an average schools is 0.066 (SE = 0.022. The estimated standard deviation of school impacts is 0.09.

To test whether tau is statistically significantly greater than zero, we use the Q-statistic proposed by Bloom et al. (2017). The Q statistic is 85.5 (df = 64, p = 0.038).

7.5 Theoretical Justification for School Moderation Analysis (Pre-registered RQ4)

Given that we have found that the treatment impact varies across schools, we now justify the approach we take for understanding this variability, which was our fourth research question.

7.5.1 Definition of Average Treatment Effects and Conditional Average Treatment Effects

Following standard notation for causal effects from the potential outcomes model[6], the individual treatment effect is defined as the difference between student i in school j's potential outcomes:

$$Y_{ij}(1) - Y_{ij}(0)$$

The *sample average treatment effect* (i.e., the treatment effect in the sample of participating students) is given by

$$\frac{1}{n}\sum_{j=1}^{m}\sum_{i=1}^{n_j} Y_{ij}(1) - Y_{ij}(0)$$

where there are m sampled schools, school j has n_j students participating in the study, and there are $n = n_1 + n_2 + \ldots n_m$ total students in the sample. Notice that all we are doing here to obtain the overall sample average treatment effect is taking the average of all participating students' individual treatment effects. The population average treatment effect is defined similarly, with the sum running over all the students in the population:

$$\frac{1}{N}\sum_{j=1}^{M}\sum_{i=1}^{N_j} Y_{ij}(1) - Y_{ij}(0)$$

where there are M total schools, school j has N_j students, and $N = N_1 + N_2 + \ldots N_m$ total students in the population. This parameter was estimated in RQ1.

Conditional average treatment effects (CATEs) are defined as the average difference in potential outcomes under treatment vs. control for individuals in a given subgroup of students g either in the sample or in the population. In RQ2, we estimated this for the population subgroup of previously-lower-achieving students. In RQ4 we will estimate conditional average treatment effects for previously-lower-achieving students attending particular types of schools, such as students in high-achieving schools or students in medium-achieving schools.

The population average treatment effect, then, is simply the weighted average of all of the population conditional average treatment efects for all of the subgroups g:

$$\frac{1}{N}\sum_{j=1}^{M}\sum_{i=1}^{N_j} Y_{ij}(1) - Y_{ij}(0) = \sum_{g=1}^{G}\frac{N_g}{N}\left(\frac{1}{N_g}\sum_{i\in g} Y_{ij}(1) - Y_{ij}(0)\right)$$

where N_g is the number of students in group g. Here $i \in g$ indexes the students in subgroup g in the population. We can represent all the averages in (7.5.1) as conditional expectations over the population:

$$\mathrm{E}_p[Y_{ij}(1) - Y_{ij}(0)] = \sum_{g=1}^{G}\frac{N_g}{N}\left(\sum_{i\in g}\mathrm{E}_p[Y_{ij}(1) - Y_{ij}(0) \mid i \in g]\right)$$

[6] Imbens, G. W., & Rubin, D. B. (2015). *Causal inference for statistics, social, and biomedical sciences: An introduction* Cambridge: Cambridge University Press.

With a probability sample, we can estimate these population conditional average treatment effects consistently provided that we make appropriate adjustments for the sampling design (described below)

Next, we present the specific estimands of interest.

7.6 School Moderator Definitions and Analyses (Pre-registered RQ4)

We tested two school moderators:

1. School achievement level = Composite of standardized achievement (PSAT, AP scores) and other indicators (see Tipton, Yeager et al. in press for information on how this was generated).
2. School challenge-seeking norms = school mean number of "hard" problem selections among control students on the worksheet task (called school mindset saturation, behavioral operationalization in the pre-registration). We also considered a self-reported operationalization as a secondary measure, based on the concerns stated in our pre-registration.

7.6.1 School Achievement Level

Following the pre-registered analysis plan, we divide schools into 3 categories based on a school achievement composite (see Tipton, Yeager, et al., in press, for details on the composite construction).

- The low-achieving group is schools at or below the 25th percentile for school achievement level.
- The medium-achieving group is schools between the 25th and 75th percentile for school achievement level.
- The high-achieving group is schools at or above the 75th percentile for school achievement level.

7.6.2 School Challenge-Seeking Norm

The school challenge-seeking norm is defined as the prevalence of growth mindset-relevant beliefs and behavior in the school environment (in the pre-registration, we called this "mindset saturation").

We test the hypothesis that there will be larger effects on GPA in higher challenge-seeking norm schools. The reason why might be that the environment reinforces the message over time. Giving the intervention in a high mindset norm schools might be like "planting a seed in tilled soil."

At the same time, it was possible that students might benefit most when they attend schools with unsupportive norms. Giving the intervention in low mindset norm schools might be like "water on parched soil."

Challenge-seeking, as noted previously, is measured by the number of hard problems selected on the behavioral make-a-math-worksheet task. For model summarization purposes, after estimating models that used the full behavioral norms measure (as pre-specified), we label schools with above (below) the population mean number of hard problems selected as high (low) challenge-seeking norm.

7.6.3 Defining Conditional Average Treatment Effects for School Achievement and Mindset Norms

Having plotted variability in treatment effects, by our two pre-specified moderators, here we define the estimands of interest more formally, and explain our approach to modeling them.

Under randomization to treatment and SUTVA (Imbens & Rubin, 2015),

$$\mathrm{E}[Y_{ij}(z) \mid i \in g] = \mathrm{E}[Y_{ij} \mid T_{ij} = t, i \in g]$$

where $i \in g$ indexes the students in subgroup g, $Y_{ij}(z)$ are the potential outcomes for each student under the different treatment statuses z, and T_{ij} is the random assignment to treatment or control.

To get estimates of these conditional expectations, we can use mixed effects models:

$$Y_{ij} = (\theta(x_{ij}) + \alpha_j) + (\lambda(q_{ij}) + r_j)\, T_{ij} + \epsilon_{ij}$$

where $\mathrm{E}[\epsilon_{ij}] = 0$, $\mathrm{Var}[\epsilon_{ij}] = \sigma^2$.[7] In this model α_j is a school-level intercept (treated as fixed in the linear mixed effects models, following Bloom et al (2017)[8], and random $N(0, \phi^2)$ in the Bayesian models, $\theta(x_{ij})$ is a function of school and individual level control covariates, centered at the school level and collected in a vector x_{ij} (see the pre-registration for definitions), q_{ij} is a variable or variables defining subgroups of interest, $\lambda(q_{ij})$ is the subgroup-dependent portion of the treatment effect for student i, and r_j is random school-level variability in treatment effects, modeled $N(0, \tau^2)$.

Under this model, for a student with $Q_{ij} = q_{ij}$ in school j,

$$\begin{aligned}
\mathrm{E}[Y_{ij}(1) - Y_{ij}(0) \mid Q_{ij} = q_{ij}] &= \mathrm{E}[Y_{ij}(1) \mid Q_{ij} = q_{ij}] - \mathrm{E}[Y_{ij}(0) \mid Q_{ij} = q_{ij}] \\
&= \mathrm{E}[Y_{ij} \mid T_{ij} = 1, Q_{ij} = q_{ij}] - \mathrm{E}[Y_{ij} \mid T_{ij} = 0, Q_{ij} = q_{ij}] \\
&= \lambda(q_{ij}) + r_j
\end{aligned}$$

(Note that since the treatment effects do not depend on the control variables x_{ij}, they are omitted from the conditioning set in the conditional expectations above.)

In the BCF analyses we report estimated changes in conditional average treatment effects for a given change in moderators, namely an increase in mindset norms (as measured by challenge-seeking behavior on the make-a-worksheet task at baseline) of 0.5 of a difficult question (out of 8). Mathematically, this is simply the difference between two conditional average treatment effects:

$$\mathrm{E}[Y_{ij}(1) - Y_{ij}(0) \mid Q_{ij} = q'_{ij}] - \mathrm{E}[Y_{ij}(1) - Y_{ij}(0) \mid Q_{ij} = q_{ij}]$$

where q is constructed using the realized moderators (including norms) and q' is the same, except the continuous norms variable is increased by 0.5.

In order to estimate population (conditional) average quantites it is necessary to account for the complex sampling design of the study. Unless the conditioning set q_{ij} includes all the variables used to determine the probability of selection, model-based estimates of the treatment effects will be biased when computed naively using sample data. In the linear mixed effects models we adjusted for the complex sampling design by maximizing a weighted likelihood function constructed to estimate the population likelihood, rather than the (biased) sample likelihood, given the over-sampling of rare subgroups of schools and modest non-response. In the Bayesian models, we include the sampling weight as a control and as an effect moderator, and estimate population expected values using the relationship:

$$E(Y_{ij}(1) - Y_{ij}(0) \mid q_{ij} = q_{ij}) \approx \frac{\sum_{j=1}^{J} \sum_{i=1}^{n_j} w_{ij} E(Y_{ij}(1) - Y_{ij}(0) \mid q_{ij} = q_{ij}, w_{ij})}{\sum_{j=1}^{J} \sum_{i=1}^{n_j} w_{ij}}$$

where w_{ij} is the sampling weight for student i in school j, and substituting model-based estimates for the conditional expectations on the right-hand side of the equation, similar to model-based post-stratification[9]. Conditioning on the sampling weight makes the sampling design ignorable, enabling consistent estimation of the population expectations on the right-hand side of the equation from models fit to the sample data.

Raudenbush & Bloom (2015) note that we might expect the error variance to depend on the treatment arm when there is unmodeled heterogeneity. However, the bias due to ignoring this heteroskedasticity depends on the magnitude of the difference between the two variances. We expect any bias is very small, since the difference in the error variances is due to unmodeled treatment effect heterogeneity and the range of heterogeneous treatment effects is small relative to the unexplained variability in outcomes.

[7] U.S. Department of Education, Office for Civil Rights. (2018, September 6). Civil Rights Data Collection (CRDC). Retrieved September 6, 2018, from https://ocrdata.ed.gov/

[8] Bloom, H. S., Raudenbush, S. W., Weiss, M. J., & Porter, K. (2017). Using multisite experiments to study cross-site variation in treatment effects: A hybrid approach with fixed intercepts and a random treatment coefficient. *Journal of Research on Educational Effectiveness, 10(4),* 817-842

[9] Gelman, Andrew. Struggles with survey weighting and regression modeling. *Statistical Science, 22* (2007): 153-164.

7.7 Pre-registered Mixed Effects Regression Models Testing School-level Moderators (RQ4)

Here is the primary model we used to answer our fourth and primary research question:

4. Do school-level factors explain the variability in the size of the CATE of the GM (on GPA for previously lower-performing students) in U.S. public high schools?

Following the pre-registration plan, the analytic model for RQ4 was a multilevel model:

Level one (students):

$$Y_i = \alpha_j + \beta_j(T_{ij}) + \gamma(P_{ij}) + \sum_{k=1}^{K} \theta_k(X_{kij}) + +e_{ij}$$

Level two (schools):

$$\beta_j = \beta + \lambda_A(A_j) + \lambda_M(M_j) + \lambda_N(N_j) + r_j$$

with:

$$e_{ij}\ N(0, \sigma_T^2)$$
$$r_j\ N(0, \tau_T^2)$$

Where:

- Y_ij is the outcome for student i in school j (GPA)
- α_j is a school-specific intercept
- T_ij is an indicator for experimental group (1 if treatment, 0 if control)
- P_ij is the prior achievement for student i in school j, z-scored within schools
- X_kij school-mean-centered baseline covariate k for each student i from school j
- A_j is the grand-mean-centered achievement level for school j, coded continuously
- M_j is the grand-mean-centered percent minority (black, Hispanic, Native American) in school j
- N_j is the school challenge-seeking norm on its natural metric from 0 to 8 for school j

As for RQ3, school moderation hypothesis tests do not employ survey weights. (Survey weights were later applied to generate the conditional average treatment effects reported in the main paper, so that estimated effect sizes generalized to the population of inference).

8 Multilevel Bayesian Causal Forest Model

The multilevel Bayesian causal forest model is specified as

$$Y_{ij} = (\theta(x_{ij}, w_{ij}) + \alpha_j) + (\lambda(q_{ij}, w_{ij}) + r_j)\,T_{ij} + \epsilon_{ij}$$

Note that unlike the linear model, the sampling weights w_{ij} are included among the controls and the moderators as discussed above. The other moderators q_{ij} include our continuous measure of challenge-seeking norms, the school achievement categories, and the percent minority variable. Multilevel BCF generalizes the linear mixed effects model by allowing θ and λ to include interactions and nonlinearities of the variables in their arguments. These features are inferred from the data and need not be pre-specified. However, this requires the use of prior distributions to avoid overfitting. Specifically, we use Bayesian additive regression tree (BART) priors on θ and λ. These prior distributions encode conservative beliefs about λ in particular:

The prior on the λ function is centered on a constant function at zero, and the prior favors simple forms for λ such as additive functions. Our prior specification follows Hahn et al. (2018)[10]. The multilevel version above uses standard prior distributions for α_j and r_j (normal, with half-Cauchy priors on their standard deviations; c.f. Gelman, 2006[11]).

8.0.1 Interpreting the Results of the Bayesian Causal Forest Model

In addition to reproducing the primary analyses' results, the Bayesian analysis added three contributions beyond the conclusions of the main analysis.

First, the treatment effects on math or science GPA revealed that lower-achieving schools' estimated treatment effects fell between higher-achieving schools' and medium-achieving schools' effects. This is reflected by even posterior odds that lower- and higher-achieving schools differed, pr(CATEAch=High > CATEAch=Low) = .49, and a moderate probability that medium- and lower-achieving schools differed pr(CATEAch=Medium > CATEAch=Low) = .78, both updated by the data from a prior probability of .5. This result matched our pre-specified hypothesis that the lowest-achieving schools in the U.S. may not have as much access as other schools to the formal resources needed to sustain the effects of an initial boost in motivation from the mindset intervention. Therefore, this result justifies future research into the potential minimal achievement level needed to produce a growth mindset treatment effect on GPA.

Second, the BCF model generated information that could serve as the basis for future research on the causal effects of growth mindset norms. We used the fitted model to estimate the increase in growth mindset treatment effects that could be expected under the hypothetical scenario in which schools were moved from being a low-norm school to being a high-norm school, assuming all other school- and student-level characteristics were left untouched (as in a random-assignment experiment). To estimate this, we used the model parameters to draw new posterior probability distributions for the average treatment effect for each low-norm school, but with norms set to a level corresponding to an increase of 0.50 additional challenging math problems chosen (out of 8), which is roughly the size of the school-level IQR. All other characteristics of students and schools were fixed at their true levels in the data. The original posterior distributions of treatment effects for each low-norm school were then subtracted from the counterfactual distributions, yielding a posterior distribution for the expected increase in treatment effect due to improvements in the norms holding all other moderators constant. The average increase in treatment effect expected for low-norms schools was .031 grade points (95% PI, -.012, .135), relative to the original distribution of treatment effects within the subgroup of low-norms schools (.024, 95% PI, -.096, .103). Thus, the model estimated a 130% increase. In other words, the treatment would be more than twice as effective on average. Moreover, the partial effect of norms on the size of the treatment effect was not different across school achievement levels or racial composition, justifying the primary linear model specification.

Third, the BCF Extended Data figure shows that was some heterogeneity that remained unexplained even after accounting for the pre-specified moderators. Therefore, exploratory analyses might be able to advance theory about the mechanisms for long-run growth mindset effects even further.

[10] Hahn, P. R., Murray, J. S., & Carvalho, C. (2018). Bayesian regression tree models for causal inference: regularization, confounding, and heterogeneous effects. Retrieved from http://arxiv.org/abs/1706.09523.

[11] Gelman, A. (2006). Prior distributions for variance parameters in hierarchical models (comment on article by Browne and Draper). Bayesian analysis, 1(3), 515-534.

9 Complier Average Causal Effects

Students were randomized at the start of the computerized activity, and received the allocated intervention for session 1. Therefore, the intent-to-treat (ITT) sample was defined to include all students who started session 1 regardless of whether they completed the key intervention components, and/or engaged with the treatment message, and/or if they saw the session 2 materials. The ITT effect on core academic GPA of the students is the most conservative, policy-relevant effect of interest because it provides an estimate of the average impact of intervention.

As a secondary step, we estimate the causal effect of the treatment on those students who engaged adequately with the treatment message-or those who "took-up" the treatment. However, defining treatment take-up in an online social-psychological intervention is not straightforward. Engagement with the treatment message can be measured in several ways-such as did the students complete the key modules of the intervention? Did the students internalize the treatment message by responding to open-ended questions asked immediately after the treatment materials? Indeed, ITT estimates under-estimate the causal effectiveness of the treatment in the case of partial non-compliance. Thus, we estimate the complier average causal effect (CACE) of the growth mindset treatment under certain assumptions.

We use a key measure of engagement with the treatment to define treatment "take-up". At the end of session 1, students in the treatment condition were asked to write a note to future students who may be struggling in their freshman year (Open Response Prompt B above). This "saying-is-believing" exercise used in past successful social-psychological interventions (Walton & Cohen, 2011[12]) has shown to be effective and integral in helping students internalize the treatment message (Yeager et al., 2016[13]).In other words, we identify those students who wrote a note to future students as those who internalized the treatment and therefore "took up" the treatment.

Next, we estimate the CACE, also known as the "treatment on the treated" (TOT) effect, by instrumenting for treatment take-up with the randomized treatment assignment indicators T_i defined earlier in a two-stage least squares (TSLS) framework. We follow standard best practices (Imbens & Angrist, 1994[14]; Angrist et al., 1996[15]; Bloom, 1984[16]) including guidelines set forth by the What Works Clearinghouse[17] to estimate the CACE. Formally, we estimate specifications of the form:

$$Y_i = \alpha_1 + \beta_1(D_i) + \gamma_1(P_i) + \sum_{k=1}^{K} \theta_1 k(X_{ki}) + \sum_{j=1}^{J} \rho_1 j(S_{ji}) + u_i$$

Where:

- Y_i is the outcome for student i (GPA)
- D_i is an indicator for the endogenous treatment take-up indicator (1 if "took-up" treatment, 0 if control). The indicator D_i is instrumented with T_i, an indicator for experimental group (1 if treatment, 0 if control) in a TSLS framework
- P_i is the prior achievement for student i, z-scored within schools
- $X_k i$ is a vector of school-mean-centered baseline covariates k for each student i
- $S_j i$ is a set of indicator variables indicating that student i attends school j

[12]Walton, G. M., & Cohen, G. L. (2011). A brief social-belonging intervention improves academic and health outcomes of minority students. *Science*, 331(6023), 1447-1451.

[13]Yeager, D. S., Romero, C., Paunesku, D., Hulleman, C. S., Schneider, B., Hinojosa, C., ... & Trott, J. (2016). Using design thinking to improve psychological interventions: The case of the growth mindset during the transition to high school. *Journal of Educational Psychology*, 108(3), 374.

[14]Imbens, G. W., & Angrist, J. D. (1994). Identification and estimation of local average treatment effects. *Econometrica*, 62, 467-475.

[15]Angrist, J., Imbens, G., & Rubin, D. (1996). Identification of causal effects using instrumental variables. *Journal of the American Statistical Association*, 91, 444-472.

[16]Bloom, H. (1984). Accounting for no-shows in experimental evaluation designs. *Evaluation Review*, 8(2), 225-246

[17]What Works Clearinghouse (n.d) Reviewer Guidance for Use with the Procedures and Standards Handbook version 3.0. Retrieved from https://ies.ed.gov/ncee/wwc/Docs/ReferenceResources/wwc_reviewer_guidance_030416.pdf

We estimate parameters using person-level weights and robust standard errors.

Formally, the First-stage equation in the TSLS framework is:

$$D_i = \chi_i + \lambda_{2i}(T_i) + \gamma_{2i}(P_i) + \sum_{k=1}^{K} \theta_{2k}(X_{ki}) + \sum_{j=1}^{J} \rho_{1j}(S_{ji}) + \quad {}_i$$

Under certain assumptions, described briefly below, we can show that the CACE estimates are valid when using the TSLS instrumental variable approach discussed above:

- (1): Random assignment: Experimental group assignment is random
- (2): One-sided non-compliance - i.e., control group members are restricted access to treatment altogether
- (3): Valid exclusion restriction- i.e., treatment assignment works entirely through the internalization of treatment message as measured by student response on a saying-is-believing exercise

While assumptions (1) and (2) are easily satisfied in a randomized intervention, the exclusion restriction cannot be directly verified. However, there is sufficient theoretical evidence to show that the "saying-is-believing" exercise may be an essential component of the treatment exercise. This writing exercise enables the student to internalize the growth mindset message through reflection after adequately engaging with the treatment materials (Aronson, 1999[18]; Yeager et al., 2016; Yeager & Walton, 2011). Therefore, while the exclusion restriction might not be strictly true, we believe that the effect of the treatment on those who did not engage with the materials and/or internalize the treatment message are likely to be much smaller. Future research should explore the determinants of take-up and exploit variation in take-up rates across schools to explore additional mediating mechanisms of the intervention.

The Stata code used to estimate parameters of this model is as follows:

```
xi: ivreg2 gpa__post__avg pregpa__imputed__smc pregpa__missing__smc pretest__imputed__smc
pretest__missing__smc s1__exp__suc__1__imputed__smc s1__exp__suc__1__missing__smc pre__gpa__self__smc
pre__gpa__self__dummy__smc gender__smc asian__smc black__smc hisp__smc native__smc
mideast__smc pacisl__smc white__smc pared__1__smc pared__2__smc pared__3__smc pared__4__smc
pared__5__smc pared__6__smc pared__7__smc pared__8__smc ell__smc sped__smc gt__smc
firstyearfreshman__smc lunch__smc i.schoolid (takeup = treatment) [pweight = g9wt], first
```

Here are the results for the CACE analyses:

9.1 Estimated CACE on Core Academic GPA for All Compliers

estimate	stderr	z	p	n	n_schools	first_stage_F	compliance_treatment
0.055	0.016	3.36	0.001	12490	65	94474.67	0.945

9.2 Estimated CACE on Core Academic GPA for Compliers Among Previously Lower-achieving Students

estimate	stderr	z	p	n	n_schools	first_stage_F	compliance_treatment
0.107	0.025	4.2	<0.001	6320	65	44617.92	0.939

[18] Aronson, E. (1999). The power of self-persuasion. *American Psychologist*, 54, 875-884. http://dx.doi.org/10.1037/h0088188

10 Methodological Information About the School Challenge-seeking Norms Measure

In this section, we assess the predictive validity of the school challenge-seeking behavioral measure, which we label challenge-seeking norms. Recall that this measure consists of the mean number of hard items selected on the make-a-worksheet task among the control group students in each school.

We test whether challenge-seeking norms predicts school-level AP mathematics course-taking for previous cohorts of students, using data collected from official administrative sources. Results show that the behavioral measure is predictive of advanced mathematics course-taking, even when controlling for average scores on standardized tests administered earlier in high school to previous cohorts of students.

10.1 Distribution of School Mindset Norms Measure (Average Number of "Hard" Items Selected by Schools' Control Group)

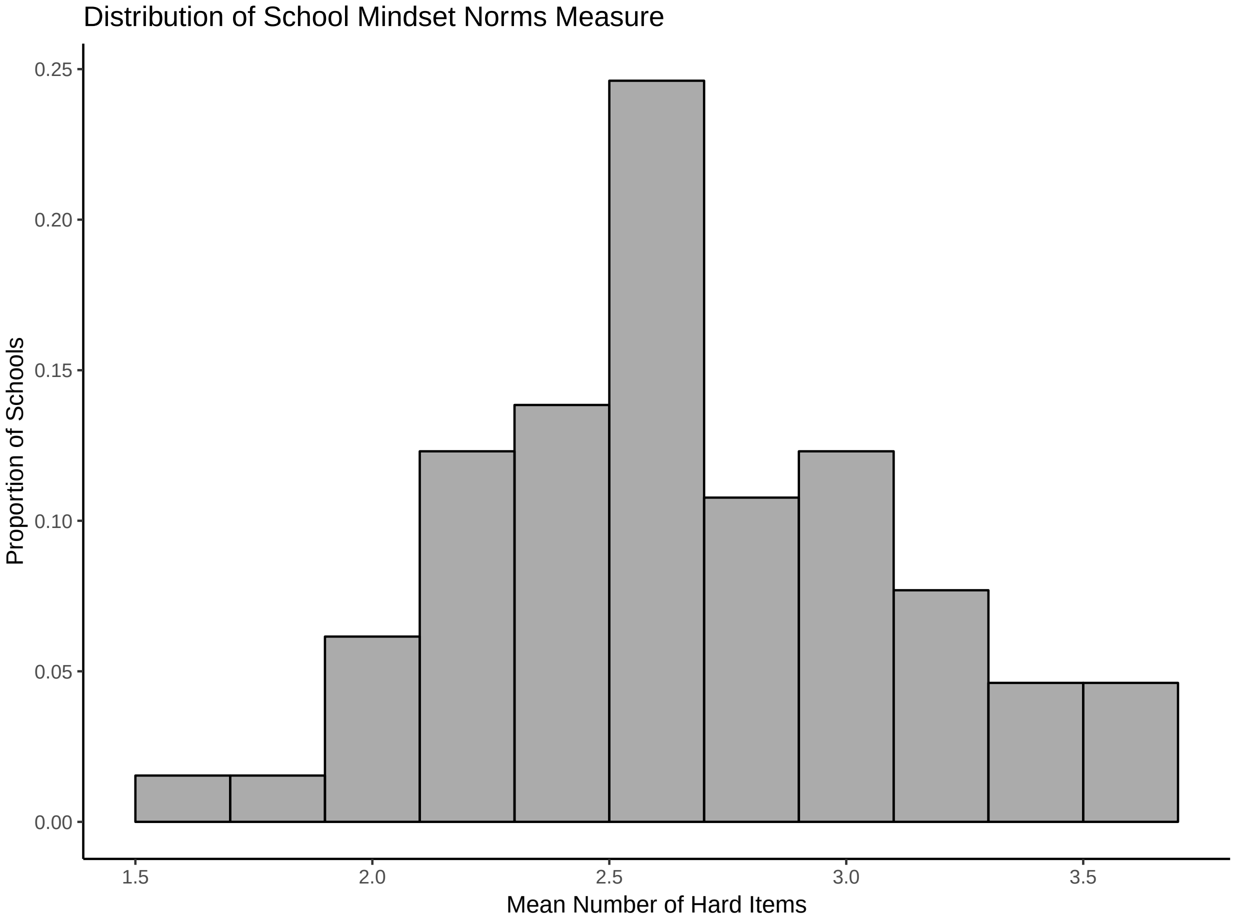

10.2 Mindset Norms and Mathematics AP: All schools

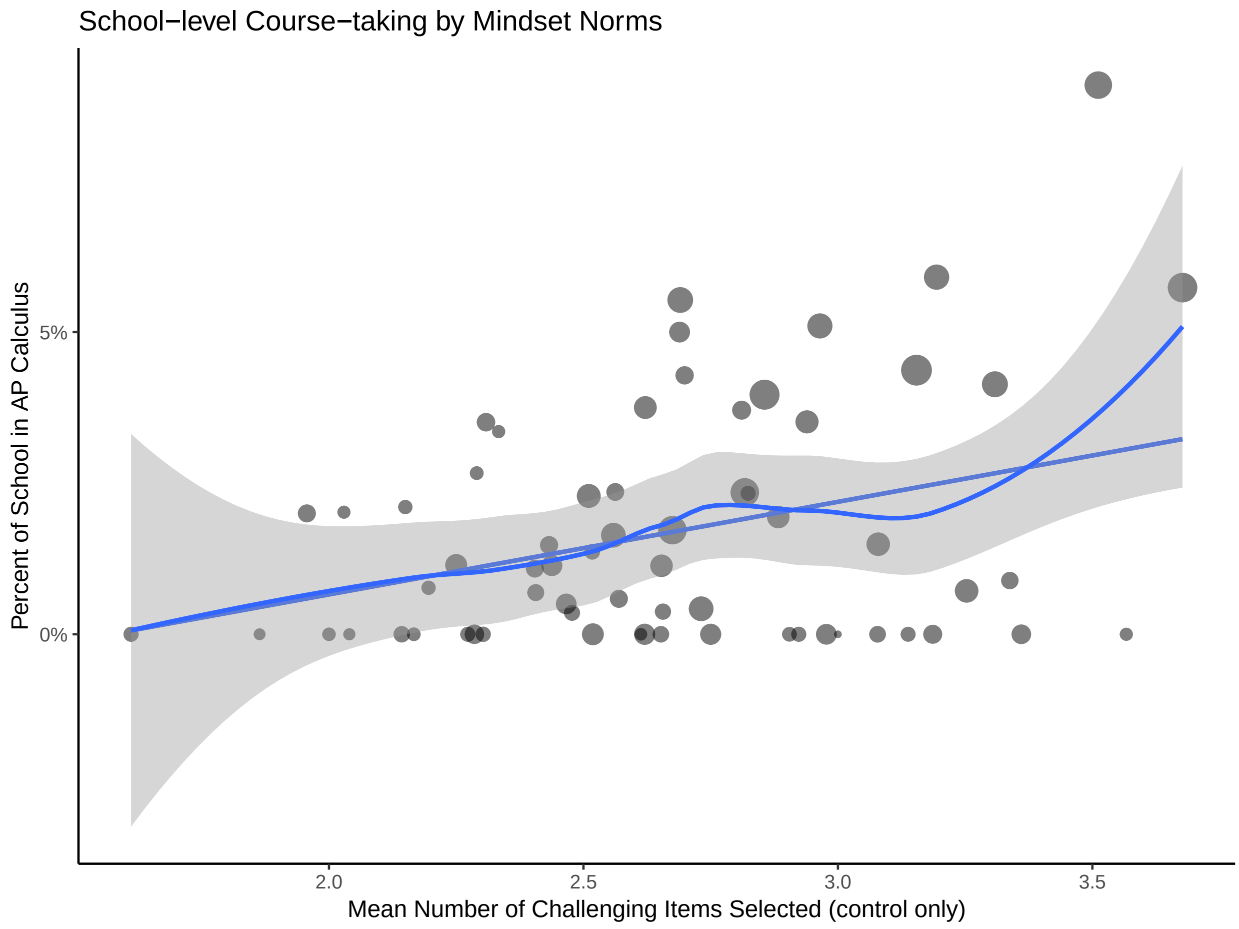

Table 28: Estimates and Standard Errors for Linear Regression Models of School-level AP Calculus Course-taking

	(1)	(2)
School Mindset Norms	0.015***	0.014***
	(0.005)	(0.005)
Mean Standardized Test Score (std)		0.004
		(0.003)
Test Missing Indicator		0.016***
		(0.006)
Mean Mathematics PSAT (std)		0.003
		(0.003)
PSAT Missing Indicator		−0.026**
		(0.010)
Proportion Black/Hispanic (std)		0.0002
		(0.003)
Constant	−0.024	−0.022
	(0.014)	(0.014)
Observations	65	65
R^2	0.114	0.350
Note:	*p<0.1; **p<0.05; ***p<0.01	
	std = standardized variable (mean 0, sd 1)	

10.3 Mindset Norms and Mathematics AP: Schools with AP Data Available Only

Here, we repeat the validity analysis excluding the schools with 0% of students taking AP. This supports the same conclusion.

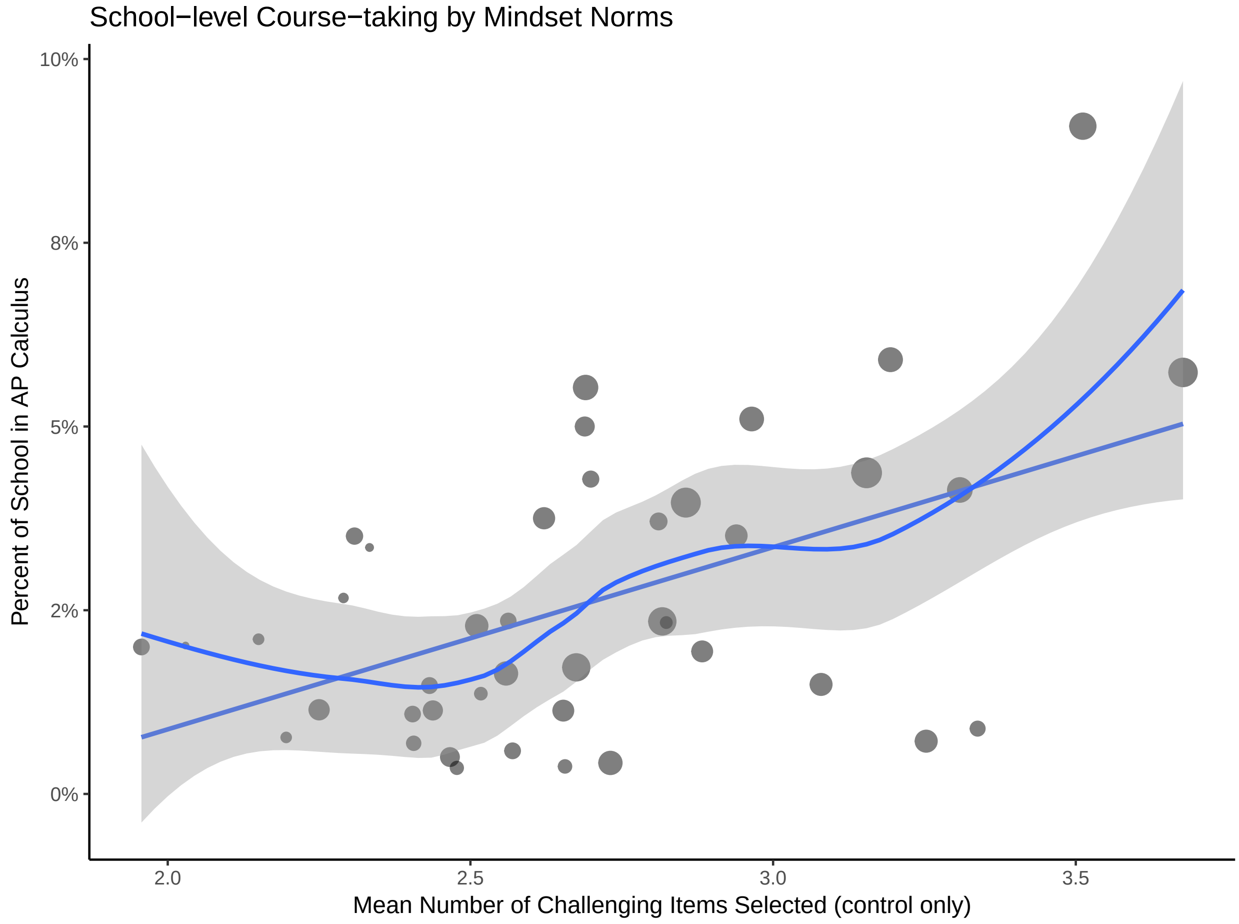

Table 29: Estimates and Standard Errors for Linear Regression Models of School-level AP Calculus Course-taking

	(1)	(2)
School Mindset Norms	0.025***	0.020***
	(0.007)	(0.007)
Mean Standardized Test Score (std)		0.003
		(0.003)
Test Missing Indicator		0.019***
		(0.006)
Mean Mathematics PSAT (std)		0.003
		(0.003)
PSAT Missing Indicator		
Proportion Black/Hispanic (std)		−0.0003
		(0.003)
Constant	−0.041**	−0.032*
	(0.018)	(0.018)
Observations	42	42
R^2	0.255	0.454
Note:	*p<0.1; **p<0.05; ***p<0.01	
	std = standardized variable (mean 0, sd 1)	

11 Cohort Analysis of i3 Evaluation Effect Sizes

The manuscript summarizes a "cohort analysis" of studies from the U.S. federal government's i3 initiative. The conclusion of this analysis was that it is rare for studies with adolescents to exceed what Kraft (2018) called a "large" effect for a randomized trial in education: .20 SD.

Here we provide greater detail about the information in the report that led to that conclusion. Abt Associates summarized the results of 67 evaluation studies funded by the U.S. Institute of Education Sciences as a part of the i3 initiative (Boulay et al., 2018). These studies are informative because they involved attempts to obtain causal effects (via random assignment experiments) on objective academic outcomes (e.g. grades or test scores) and the evaluators had to pre-register the study and pre-specify the outcome(s) of interest. Since the evaluation studies met several criteria for rigor prior to winning the funding (prior evidence, importance, and strong evaluation design) this cohort of studies is useful for generating an empirical distribution of the effect sizes for promising education interventions evaluated in rigorous randomized trials. Sixty-six of the studies listed in the Abt report prespecified their analysis plans, 48 of these evaluations examined outcomes among adolescents (middle school or high school), and 13 of these involved a program administered in an existing school and reported at least one pre-registered outcome that met the highest standard of rigor (the What Works Clearinghouse standard of "without reservations"), which qualified them for the present analysis. One of these effects (the scale-up of KIPP, a charter network) was excluded because it is not comparable to a typical educational intervention program, which involves adding programming or training in an existing

school (not starting a new school). When a program reported multiple pre-registered primary outcomes, Those effect sizes were averaged.

The unweighted average effect size in this cohort of pre-registered studies was .03 SD (when including KIPP it was .04 SD), which was "small" according to Kraft (2018). Two programs (17%) showed a significant and "large" effect (.20 SD and .23 SD, respectively). One of these effects (.20 SD) came on tests developed by the research team, not on grades or state tests. Because effect sizes on researcher-created tests are known to be larger (Cheung & Slavin, 2016) then it was less relevant to the present study as a comparison. Only one program in this cohort analysis was successful at raising adolescents' grades. Last, fully 75% (9/12) showed effects smaller than .10 SD (the growth mindset intervention effect for the targeted group of lower-achieving students), regardless of the outcome.

12 Independent Contractor's Methods for Processing the Grades Variables

12.1 Overview

A team of PhD and master's-level social science researchers and computer scientists at MDRC processed the grades data that had been delivered by the schools to ICF International (who cleaned and merged the raw grades files). MDRC's processing created the focal variables to estimate treatment impacts: pre-intervention core course (English, math, science, and social studies) grade point average (GPA) and post-intervention core course GPA.

It was necessary to harmonize this information across the schools because the grades data were provided by many different districts with many different naming conventions for course names and grading periods. MDRC's coding process drew on both human judgment and automated methods. This coding was conducted blind to students' condition assignments and blind to the impact of various decisions on estimates of treatment impact. All decisions were checked by a second coder and discrepancies were resolved through discussion or by revising the coding scheme. This was done so that the coding decisions could be reproducible and standardized. MDRC's study director can be contacted with queries about the technical details of the coding process (Pei Zhu: Pei.Zhu@mdrc.org).

The work carried out by MDRC and described here occurred in two phases:

1. Coding course names into specific subject areas (English, math, science, and social studies);
2. Coding grading periods to determine the pre- and post-intervention epochs and writing data analysis syntax to construct the pre- and post-intervention grades variables used in analyses.

12.2 Phase 1: Coding Course Names

In Phase 1, MDRC executed this aspect of the pre-analysis plan (p. 7):

"Core course designation will be made through a combination of course catalogs from schools and coding of course names. Coding of core courses will be independent of knowledge of the effect on outcomes of the study, and all syntax will be retained to enable robustness checks."

Phase 1 began by examining the schools' official course catalogues to determine the core classes dictated by the schools' curricula. When course names appeared in both the dataset and the course catalogue, then a straightforward coding of the course names was executed. When the course catalogues and course names did not match, which was the case for many courses in many schools, human coders determined core course classifications. They did so by applying what is known about high school course offerings and by examining cross-tabs of course enrollments to make logical inferences.For example, a course titled "Geo" could be geometry (i.e. math) or geology (i.e. science) or geography (i.e. social studies). But if the cross-tabs showed that "Geo" was mutually exclusive with Algebra 1 or 2 and often co-occurred with biology and world history,

then the coders might infer that the course was geometry, not geology or geography. MDRC developed routines for flagging ambiguities, and these were resolved by pairs of human coders who made the final determinations and documented the reasons for their decisions. As a last step, MDRC used text patterns to detect all the courses that seemed like they could potentially be a core course, with a focus on students who were missing a core class for a given grading period, and two coders then validated them by hand.

12.3 Phase 2: Calculating GPAs

MDRC executed the pre-registered decision rules for determining pre- and post-intervention GPA, described on page 7 of the pre-analysis plan. That is, MDRC transformed the grades file so that it had one value per student for each subject area for the pre- and post-intervention epochs. Merging and data aggregation syntax was written by a trained computer scientist and checked and commented by a team of experienced social scientists.

Depending on when the intervention was given within a school, MDRC assigned different grading period GPAs as before pre/post treatment for each school, following the analysis plan (p. 7). MDRC decided how to handle the case where a student had more than one grade for the same marking period, which could happen if schools allocate units in half-unit metrics (i.e., if the fall semester and spring semester show independent grades but are both listed as half of the "final" grade). In some cases, schools were re-contacted to clarify the delivered data. The next step involved standardizing all numeric and letter grades across all the schools to a scale of 0-4.3. Next, MDRC averaged together all the core courses that were taken by a student in a given grading period to evaluate the student's academic grading period GPA. MDRC also calculated GPAs for each subject area by grading period for the specific subjects (mathematics, science, English, and social studies).

The final product of this process was the syntax for creating a pre- and post-intervention GPA variable for each student. This was then shared with the primary investigators in October, 2018, who then ran it to create the analytic file. In the future, MDRC will carry out its own analyses and release its own report.

13 Pre-registration File

In the following pages we append the full pre-registered analysis plan file. This file is archived at: https://osf.io/afmb6/

Study Information

1. Title

1.1. Provide the working title of your study. It may be the same title that you submit for publication of your final manuscript, but it is not a requirement.

Average effects and cross-site variability of effects of a growth mindset intervention on 9th grade achievement in a national probability sample.

2. Authorship

Authors: David S. Yeager*, Paul Hanselman*, Carol Dweck, Chandra Muller, Robert Crosnoe, Barbara Schneider, Greg Walton, Dave Paunesku, Beth Tipton, Chris Hulleman, Angela Duckworth
* Primary authors

Input: Andy Gelman (Columbia), Jordan Axt (Virginia), Todd Rogers (Harvard), Mike Weiss (MDRC).

3. Primary Research Questions

3.1. Please list each research question included in this study.

RQ 1: What is the average treatment effect (ATE) of a Growth Mindset (GM) intervention on the GPA of 9th grade *students* in regular U.S. public high schools?

RQ 2: What is the conditional average treatment effect (CATE) of a GM intervention on the GPA of 9th grade *previously low-performing students* in regular U.S. public high schools?

RQ 3: How much does the CATE of a GM intervention (on the GPA of 9th grade *previously low-performing students*) vary across U.S. public high schools?

RQ 4: Do school-level factors explain the variability in the size of the CATE of the GM (on GPA for *previously low-performing students* in U.S. public high schools)?

GPA and *previously low-performing students* are defined in the measured variables section of the analysis plan.

4. Hypotheses

4.1. For each of the research questions listed in the previous section, provide one or multiple specific and testable hypotheses. Please state if the hypotheses are directional or non-directional. If directional, state the direction. A predicted effect is also appropriate here.

H 1: **ATE for all students.** We hypothesize a *very small* (near zero) positive effect of a GM intervention for 9th grade *students* in regular U.S. public high schools (on GPA).

H 2: **CATE for previously low-performing students**: We hypothesize a *moderate* positive effect of a GM intervention for *previously low-performing 9th grade students* in regular U.S. public high schools (on GPA).[1]

---

[1] Note: We hypothesize a near zero effect for previously high-performing students because:
a) growth mindset theory predicts improvements for struggling students, not students who are unchallenged (e.g. Burnette et al. 2013);
b) there is range restriction for previously high-achievers;

H 3: **Cross-school variation in CATE.** We hypothesize that there will be significant cross-school variation in the school-average effect of the GM intervention for 9th grade *previously low-performing students* in regular U.S. public high schools (on GPA).

H 4: **Explaining cross-school variation in CATE.** Research question 4 involves confirmatory analyses of previously-untested hypotheses. In particular, we hypothesize that:

H 4a. Among previously low-performing students, the school-average effect of the GM intervention will vary based on *school achievement level*. [2]

Directionally, we hypothesize that the CATE will be:

i. Smallest (and possibly zero) in the lowest-achievement schools.[3]
ii. Significant and positive in medium-achievement schools, and larger than in the lowest-performing schools.[4]
iii. Significant and positive, but of unknown relative magnitude, in the highest-achievement schools.[5]

Supplemental analyses will test for the effects for different strata of schools defined in the initial sampling plan.

H 4b. Among previously low-performing students, the school-average effect of the GM intervention will vary based on *school mindset saturation level*.

There are two competing directional hypotheses:[6]

i. *Larger effects on GPA in higher mindset saturation schools*. The reason why is that the environment reinforces the message over time. Giving the intervention in a high mindset saturation school is like "planting a seed in tilled soil".
ii. *Larger effects on GPA in lower mindset saturation schools*. The reason why is that in high mindset saturation schools students are already receiving growth mindset from their teachers and peers (because the control group is getting "treated") – the intervention is a "drop in the bucket". Meanwhile, in lower mindset saturation schools, students are most in need of a growth mindset – the intervention is like "water on parched soil."

We define *school achievement level* and *school mindset saturation level* below.

---

c) prior research that we are replicating (e.g. Paunesku et al., 2015; Yeager et al., 2016) only finds benefits for low-achieving students and does not focus on main effects in the full sample.

Thus, the ATE for the average student (RQ 1), which includes previously low- and high-performing students, is expected to be very small and positive. The effect for previously low-performers is expected to be moderate positive, relatively larger than for the full sample, and statistically significant.

[2] The conceptual hypothesis is that the rigor and standards in the school will interact with the treatment effect, under the theory that mindset interventions allow students to take better advantage of the instruction in the school.

[3] The rationale is that even though students in low-performing schools may face low levels of motivation, motivation may be less consequential for grades in schools with inadequate instruction or unsafe learning environments;

[4] The rationale is that student motivation may suffer in such schools and therefore may be lifted by the intervention, because instruction and learning environments are adequate but motivation is sub-optimal;

[5] The reason why we do not have predictions for higher-performing schools is that, on the one hand, they could have optimal motivation already and show weaker growth mindset treatment effects. Hence high-achieving schools might show the same effects as low-achieving schools. On the other hand higher-performing schools could have high rigor but a fixed mindset culture that could benefit more from a growth mindset treatment. Hence high-achieving schools might show stronger effects than low-achieving schools.

[6] These were first tested in Paunesku's dissertation.

Sampling Plan

In this section we will ask you to describe how you plan to collect samples, as well as the number of samples you plan to collect and your rationale for this decision. Please keep in mind that the data described in this section should be the actual data used for analysis, so if you are using a subset of a larger dataset, please describe the subset that will actually be used in your study.

5. Existing data

5.1. Preregistration is designed to make clear the distinction between confirmatory tests, specified prior to seeing the data, and exploratory analyses conducted after observing the data. Therefore, creating a research plan in which existing data will be used presents unique challenges. Please select the description that best describes your situation. Please do not hesitate to contact us if you have questions about how to answer this question (prereg@cos.io).

6. Explanation of existing data

6.1. If you indicate that you will be using some data that already exist in this study, please describe the steps you have taken to assure that you are unaware of any patterns or summary statistics in the data. This may include an explanation of how access to the data has been limited, who has observed the data, or how you have avoided observing any analysis of the specific data you will use in your study. The purpose of this question is to assure that the line between confirmatory and exploratory analysis is clear.

All of the present research questions concern the effect of an intervention on students' *GPA* assigned from the point of intervention through the end of 9th grade. Students' grades have been recorded by school districts but have not yet been delivered to the researchers for 62 of the 66 schools in the study. Most of the school districts have delivered their datasets to a third-party research firm, ICF international, which is cleaning and merging the data. ICF international has not yet shared the full grades dataset with the research team.

ICF shared an "early release" of 4 of the 66 schools to the research team so that the team could provide feedback on the data cleaning and merging process and make additional requests for formatting and information that could be applied to the full set of 66 schools. Furthermore, data from those 4 schools were cleaned and analyzed by the research team, to inform the pre-registered analysis plan.

In sum, 62 of the 66 schools' achievement data are not yet delivered to the research team by the third-party research firm. Therefore we are not yet able to test any of the four research questions above.

7. Data collection procedures.

7.1. Please describe the process by which you will collect your data. If you are using human subjects, this should include the population from which you obtain subjects, recruitment efforts, payment for participation, how subjects will be selected for eligibility from the initial pool (e.g. inclusion and exclusion rules), and your study timeline. For studies that don't include human subjects, include information about how you will collect samples, duration of data gathering efforts, source or location of samples, or batch numbers you will use.

A research firm selected a sample of schools and recruited them into the study. A school liaison, working with the research firm, helped students complete the materials in a school computer lab. The sampling plan is described in the methodological report for the study.

8. Sample size

8.1. Describe the sample size of your study. How many units will be analyzed in the study? This could be the number of people, birds, classrooms, plots, interactions, or countries included. If the units are not individuals, then describe the size requirements for each unit. If you are using a clustered or multilevel design, how many units are you collecting at each level of the analysis?

School level: We took a stratified random sample of approximately 150 high schools from the universe of *all* regular U.S. public high schools.[7] 76 schools agreed to participate and collected student survey data and 66 provided student record data. The primary analytic sample for the present study will be the 66 schools with student achievement records.

Student level: Students are nested within schools. Our target was to include all 9th grade students within each randomly selected school. Students are included in analyses of treatment effects provided that they (a) saw the first page of treatment or control content, and (b) have student records data (for calculating GPA).

There were approximately 16,000 students who began Session 1 in the 76 schools, but we do not yet know the sample size for the subset with student records because the student records have not yet been delivered.

9. Sample size rationale

9.1. This could include a power analysis or an arbitrary constraint such as time, money, or personnel.

We recruited as many schools as could be recruited in the period between April 2015 and February 2016 (when the final schools implemented the treatment). The plan was for all schools to complete the intervention by the second month of 9th grade, but we extended the window until February of 2016 to increase sample size.

10. Stopping rule

10.1. If your data collection procedures do not give you full control over your exact sample size, specify how you will decide when to terminate your data collection.

Our data collection procedures did not give us full control over exact sample size. Termination of data collection occurred when it was too late in the year to include more schools (February 2016).

Variables

11. Manipulated variables

11.1. Describe all variables you plan to manipulate and the levels or treatment arms of each variable. For observational studies and meta-analyses, simply state that this is not applicable.

[7] A list of all U.S. public high schools was obtained from the Common Core of Data (National Center for Education Statistics) and supplemented through private databases (see Tipton, Yeager et al., in press).

We manipulated the materials during individual computer activities students completed at school. Students were randomly assigned by the computer program to be presented with either a growth mindset treatment or a control activity.

12. Measured variables

12.1. Describe each variable that you will measure. This will include outcome measures, as well as any predictors or covariates that you will measure. You do not need to include any variables that you plan on collecting if they are not going to be included in the confirmatory analyses of this study.

Outcome Measure(s):

GPA: GPA serves as the single confirmatory outcome measure for all hypotheses discussed in this analysis plan. GPA refers to the end-of-the-school-year GPA based on grades in core courses only. Grades are defined as grades on a 0-4.33 point scale. Core courses refer to math, science, social studies, and English/Language Arts. Grades in these core courses will be averaged (unweighted) to calculate GPA. Plans for data processing of grades are provided in the *Indices* section of the analysis plan.

Analyses of other configurations of grades are possible, but the confirmatory GPA variable will drive the main "story" regarding the effectiveness of the GM intervention. We will also explore the GM intervention's effects:

- In specific subjects (e.g., Math).
- On "poor performance" at the end of 9th grade, such that 1 = D/F average in core courses, 0 = satisfactory performance (C- or above).

Attitudes: Students self-reported a number of attitudes at pre-test and post-test, and analyses of these were pre-registered prior to data delivery (https://osf.io/byc2e/). Students self-reported mindsets and we measured their behavior on the "make-a-worksheet" challenge-seeking task (as interim outcomes). We collected measures of treatment fidelity (described in the exploratory analyses).

Student-level subgroup(s):

To answer RQ 2-4, we must define who is considered a "previously low-performing student." We do that here.

Previously low-performing students are defined as students who were earning grades lower (or equal) to 50 percent of his or her 9th grade school peers, prior to random assignment.[8] At an operational level, this is a student whose pre-random assignment GPA is at or below the 50th percentile of his/her 9th grade peers.

School-level subgroup(s):

To answer research question 4, we must define school achievement-level and school mindset saturation level. We do that here.

School-level achievement is defined as a latent variable derived from school-level achievement data. When testing for non-linear differences, we break school-level achievement into three categories, which align with the sampling plan and the hypotheses described in section 4.1:

[8] At a theoretical level, this is a student whose grades are not already maximal and who might show higher grades if motivated.

i. *Lowest-achievement schools* are those schools in the bottom quartile of the school-level achievement index.
ii. *Medium-achievement schools* are those schools that fall above the 25th and below the 75th percentile on the school-level achievement index.
iii. *Highest-achievement schools* are those schools in the top quartile of the school-level achievement index.

The *school-level achievement index*, or measure, is described in section 13.

School-level mindset saturation is defined as the prevalence of growth mindset thinking in the school environment. A continuous variable will test the competing hypotheses described in section 4.1. The *school-level mindset saturation indices* are described in section 13.

Student-level Covariates

- Student male/female identification
- Student race/ethnicity
    - Dummy variables for Asian/Asian-American, Hispanic/Latino/a, Black/African-American, or other, with the referent group white students
- Student special education status (when available), dummy variable
- Student maternal education, dichotomized (1=four-year degree or higher, 0=less than a four-year degree)
- Student self-reported expectations for success (unless multi-collinearity with prior achievement is too high)
- ***Missing data***. We will not use list-wise deletion of cases that are missing covariates. We will impute missing covariates using the missing value dummy method, unless an alternative method is recommended by our statistician advisors.
- ***Collinearity***. We will remove a covariate from the models if it is too highly correlated with others, if there is excessive missing data, if it increases standard errors due to multi-collinearity, or if it prevents the model from converging.

13. Indices

13.1. If any measurements are going to be combined into an index (or even a mean), what measures will you use and how will they be combined? Include either a formula or a precise description of your method. If you are using a more complicated statistical method to combine measures (e.g. a factor analysis), you can note that here but describe the exact method in the analysis plan section.

For Outcome Measure(s):

Processing of grades for calculating GPA: Here is how we will process both pre- and post-intervention grades:

- We will analyze grades at the **term** level (e.g. fall or spring semester, or, in block schedules, a quarter). When only independent marking period grades are provided (e.g., marking periods 1-3, but not fall semester) then we will aggregate them to the term level (except in the case of missing pre-treatment data, as noted below).
- We will analyze only **core course** grades. We define core courses as math, science, social studies, and English/language arts. Non-core courses are electives, such as art, PE, computers or music. Non-core courses also include “support” classes, such as a lab class that is co-enrolled with a science class.
    - Core course designation will be made through a combination of course catalogs from schools and coding of course names. Coding of core courses will be independent of knowledge of the effect on outcomes of the study, and all syntax will be retained to enable robustness checks.

- If a school has a non-standard schedule (e.g. a block schedule) we may need to create a school-specific rule. We will annotate the syntax in the grades processing file, along with the justification. These decisions will be made prior to merging data with the randomized condition variable.
- Grades will be provided as letter grades (e.g., A, B, C). Core course grades will be re-coded on a 0 to 4.33 point scale, with 0 referring to "F" and 4.33 referring to "A+." Some schools will only report up to an A and so 4.0 will be the max grade for them. We will test the impact of putting all schools on the same scale (from 0 to 4).

GPA at the end of 9th grade.

- **Goal**: The main outcome is GPA in core courses at the end of 9th grade, weighting each core course equally. The initial plan was to average Fall 2015 and Spring 2016 achievement. However, some schools delivered the intervention in Spring 2016.
- **Confirmatory Operationalization.**
    - In schools that delivered the intervention in Fall of 2015, the outcome will be the average of Fall 2015 and Spring 2016 core course grades.
    - In schools that delivered the intervention in Spring of 2016, the outcome will be Spring 2016 core course grades only.
    - An exception will be if a school uses block scheduling and an entire quarter's grade is self-contained. In that event, we will look at the timing of the delivery of the treatment and the beginning and end of the quarter, to determine whether grades were recorded post-intervention or pre-intervention.
- **Missing data**.
    - We will use list-wise deletion of cases that are missing the primary outcome variable.
    - We will examine the impact of differential attrition on our inferences (for instance, perhaps the treatment kept marginal students from dropping out) and develop adjustments if attrition is differential.

<u>**For Student-level Subgroup(s)**</u>

<u>Previously low-performing students:</u>

- **Goal**. Conceptually, we wish to know if the treatment benefits students who were not already earning very high grades prior to receiving the intervention. The design of the study called for a fall intervention, and so we expected to use 8th grade achievement to define this subgroup. However some schools gave the intervention in the spring term of 9th grade (or perhaps after a full quarter's grades were recorded, for block schedules). Therefore grades from the fall of 9th grade are the most recent term. We will use the most recent term to create the pre-intervention low-performing student subgroup.
- **Confirmatory Operationalization.**
    - In schools that delivered the intervention in the Fall of 2015 prior GPA will be the average grade in 8th grade core courses, or if these were not provided, it will be 8th grade spring in core courses.
    - In schools that delivered the intervention in the Spring of 2016, the pre-intervention GPA will be the Fall 2015 GPA in core courses (i.e. Fall of 9th grade).
    - The exception to these two rules would be schools using block scheduling on a quarter system and where the treatment was delivered in the Fall but after an entire quarter's grades were recorded. In such cases, the completed first quarter Fall 2015 grades will be prior achievement and the remaining three quarters will be the outcome.
    - Pre-intervention GPA will be z-scored *within* schools.

To be precise, let:

$PreTreatGPA_{ij}$= the 8th grade GPA of student *i* attending 9th grade school *j* (among students where the study was implemented in in the fall of 9th grade), or the fall 9th grade GPA of student *i* attending 9th grade school *j* (among students where the study was implemented in the spring of 9th grade or after a Fall 8th grade block was finished).

$MedianPreTreatGPA_j$= the median pretreatment GPA of students attending 9th grade school *j*.

So:

$$Low\_performing_{ij} = \begin{cases} 1\ if\ PreTreatGPA_{ij} \leq Median\ PreTreatGPA_j \\ 0\ otherwise \end{cases}$$

Missing data.

- When students do not have 8th grade achievement but at least some of their grades were reported on a progress report in 9th grade prior to the delivery of the treatment, such as first quarter grades, then these will constitute pre-treatment GPA.
- When students do not have any of these grades, we will impute prior achievement values using their 8th grade test scores and self-reports of expectations for success in the coming year (cf. Hulleman & Harackeiwicz, 2009).

<u>For School-level Subgroup(s)</u>

<u>School-level achievement index</u>

- **Goal**. The goal for the school achievement variable is to understand whether treatment effects are different at school with different levels of rigor and standards. As a proxy for this, we created a latent variable of school achievement level for the purposes of stratification when randomly sampling schools to participate in this project (see Tipton, Yeager et al.).[9] This same latent variable will be used for subgroup analyses.
- **Confirmatory Operationalizations**.
  - There will be two operationalizations. The first is a continuous school achievement level variable, z-scored in the full population of approximately 12,000 regular U.S. public high schools (see Tipton, Yeager et al.).
  - A second operationalization is three categories of school-level achievement level: low (bottom quartile), medium (25th -75th), and high (top quartile), which is how this variable was used in the stratified sampling plan.
- **Missing data**.
  - There is no missing data on the school achievement level variable.

<u>School-level mindset saturation index</u>

- **Goal**. The goal is to assess whether environments with a strong mindset climate have weaker or stronger effects. However there is no established measure of mindset saturation.
- **Confirmatory Operationalizations.** We will test and report two operationalizations:
  - *Self-report.* The average "fixed mindset rating" on a 6-point scale for students in the school, measured prior to random assignment (both treatment and control group). The advantage of this measure is that it is a direct assessment of the construct – the prevalence of fixed/growth mindset thinking. The disadvantage of this measure is the potential for "reference bias" in making between-school comparisons (see Duckworth & Yeager, 2015). Another disadvantage is that peers may conform more to perceived actions than private beliefs. Then again, reference bias may be minimal for growth mindset (see West et al. 2017).

[9] As described in Figure 1 in Tipton, Yeager et al., the prior achievement variable was a composite of PSAT scores, AP scores, % AP Calculus test-takers, rating from greatschools.org (which are mostly composed of state test scores), and a state-level constant from the NAEP.

   - *Behavior*. The number of challenging math assignments that students chose on the make-a-worksheet task, in the control group. The advantage of this measure is that it may not be subject to reference bias. Another advantage is that the mindset saturation in a school might be communicated more by how students *act* than what students say they *believe* (e.g. Haimovitz & Dweck; also see Paluck, 2009). A disadvantage of this behavioral measure is challenge-seeking is only a proxy for growth mindset and is only modestly correlated with growth mindset.
- **Missing data**.
   - NA

Design Plan

14. Study type

This is a randomized controlled trial. The researcher randomly assigned treatments to study subjects.

15. Blinding

Personnel who interact directly with the study subjects (either human or non-human subjects) were not aware of the assigned treatments.

16. Study design

16.1. Describe your study design. Examples include two-group, factorial, randomized block, and repeated measures. Is it a between (unpaired), within-subject (paired), or mixed design? Describe any counterbalancing required. Typical study designs for observation studies include cohort, cross sectional, and case-control studies.

Two-group randomized block design. Within each school (block) students are randomized to treatment or control. In addition, stratified random sampling was used to select schools.

17. Randomization

17.1. If you are doing a randomized study, how will you randomize, and at what level?

Randomization occurs at the student level via the computer after students log in to the system. Students, teachers, facilitators and researchers are all blind to condition.

Analysis Plan

You may describe one or more confirmatory analysis in this preregistration. Please remember that all analyses specified below must be reported in the final article, and any additional analyses must be noted as exploratory or hypothesis generating.

A confirmatory analysis plan must state up front which variables are predictors (independent) and which are the outcomes (dependent), otherwise it is an exploratory analysis. You are allowed to describe any exploratory work here, but a clear confirmatory analysis is required.

18. Statistical models

RQ 1. ATE for all students.

To estimate the average treatment effects (ATE), we use the following fixed effects model:

$$Y_i = \alpha + \beta \cdot T_i + \gamma \cdot A_i + \sum_{k=1}^{K} \theta_k \cdot X_{ki} + \sum_{j=1}^{j} \rho_j \cdot S_{ji} + e_i \quad (1)$$

where:

Y_i = the outcome for student *i* (*GPA*)
T_i = 1 if student *i* was randomized to treatment and zero otherwise,
A_i = the prior achievement for student *i*, z-scored within schools
X_{ki} = school-mean-centered baseline covariate *k* for student *i* (see section 12)
S_{ji} = indicator variable indicating student *i* attends school *j*

The model will use person-level survey weights (which include school-level adjustments) and will not include any school-level covariates. It will use cluster-robust standard errors, clustered at the school level, to account for the nesting of students within schools. The parameter of interest is β, the average effect of the GM intervention for all students.

RQ 2: ATE for previously low-performing students.

To answer research question two we will use equation (1) on the subsample of previously low-performing students (i.e. students below the median within their school). The parameter of interest is β, the average effect of the GM intervention for previously low-performing students.

We use the above model (rather than the random effects model in RQ3 and 4) because RQ1 and RQ2 seek to estimate the effect for the average student, not the average school.

Below are the conclusions we would draw from the analyses in RQ1 and RQ2.

		**Full Sample (RQ 1)**	
		$\hat{\beta} > 0$, ***p*** **< .05**	$\hat{\beta} \approx 0$, ***p*** **> .05**
**Previously low-performing students (RQ 2)**	$\hat{\beta} > 0$, ***p*** **< .05**	1. Replicated Yeager/Paunesku low-performer effect and surprisingly showed a main effect as well. *Program was effective, on average, for the full sample and for previously low-performers.*	2. Replicated Yeager/Paunesku low-performer effect and replicated Yeager et al. non-significant main effect. *Program was effective, on average, for previously low-performing students. Results were as expected.*
	$\hat{\beta} \approx 0$, ***p*** **> .05**	3. Failed to replicate Yeager/Paunesku low-performer effect, but surprisingly showed a main effect. *Program was effective, on average, for the full-sample, but not effective, on average, for the expected subgroup.*	4. Failed to replicate Yeager/Paunesku low-performer effect, replicated non-significant main effect. *Program was not effective, on average, for all students or for low-performers.*

RQ 3: Variability in effects across schools, among previously low-achieving students.

To estimate variability in the treatment effect across schools, we will estimate a mixed effects model in the subset of previously low-performing students, using the model described by Bloom et al. (2017):

Level one (students)

$$Y_{ij} = \alpha_j + \beta_j \cdot T_{ij} + \gamma \cdot A_{ij} + \sum_{k=1}^{K} \theta_k \cdot X_{kij} + e_{ij} \qquad e_{ij} \sim N(0, \sigma_T^2) \quad (2)$$

Level two (schools)

$$\beta_j = \beta + r_j \qquad r_j \sim N(0, \tau^2) \quad (3)$$

where:

Y_{ij} = the outcome for low-achieving student *i* from school j (GPA)
T_{ij} = 1 if student *i* from school *j* was randomized to treatment and zero otherwise,
A_{ij} = the prior achievement for student *i* from school j, z-scored within schools
X_{kij} = school-mean-centered baseline covariate *k* for student *i* from school j, (see section 12)

For each school (j) this model allows for a fixed school-specific intercept (α_j), to account for the possibility of differences across schools in the proportion of students who are randomly assigned to the treatment vs. control group. This model allows for the treatment effect among low-achieving students to vary randomly across schools, β_j, with variance τ^2. Note that the model allows the student-level residual variance to be different for treatment and control group members (represented by the subscript in the term σ_T^2). These analyses will use survey weights and not include any school-level covariates. The parameter of interest for RQ 3 is τ, the standard deviation of the school-level distribution of average treatment effects.

We will conclude the intervention effects vary across schools when either a permutation test or a Q-statistic from meta-analysis shows that τ is different from zero (see Bloom, Raudenbush, Weiss & Porter, 2016). We will interpret the practical significance of our estimate of τ by comparing it to published benchmarks in program evaluation research (Weiss et al., 2017).

Here are the conclusions we would draw from the analysis in RQ3:

- $\hat{\tau} > 0$, p < .05: The effectiveness of the GM intervention varies across schools.
- $\hat{\tau} \approx 0$, p > .05: There is no discernable evidence that the effectiveness of the GM intervention varies across schools.

If $\hat{\tau}$ > 0 and p < .05, we will also estimate and graphically present the school-level distribution of average GM effects, as described in Bloom, Raudenbush, Weiss & Porter, 2016.

RQ 4: Predicting variability in effects across schools, among previously low-achieving students. Regardless of the answer to RQs 1-3, we will test whether school factors predict variation in the GM intervention's effects among schools —i.e. the β_j's in equation (3). The moderators are school achievement level and mindset saturation. All models will control for percent minority (black, Latino/a, or Native American) because these could be confounded with school achievement level and mindset saturation. These are confirmatory analyses of exploratory hypotheses – thus the approach to the analyses is more flexible than the approach for RQ 1- 3. This will also require a more cautious interpretation.

To preview, we will conduct four parametric tests:

1. School achievement as a continuous variable
2. School achievement as a categorical variable
3. Mindset saturation assessed via self-reports
4. Mindset saturation assessed via behavior

Then we will estimate a flexible, non-parametric model that likely will use Bayesian inference.

As a first test, we will examine independent, linear predictors. Specifically, we estimate a two-level mixed effects model. The level 1 model is specified in equation (2). The level 2 model is in equation (4) below:

<u>Level two (schools)</u>

$$\beta_j = \beta + \delta \cdot A_j + \pi \cdot M_j + \lambda \cdot S_j + r_j \qquad r_j \sim N(0, \tau^2) \qquad (4)$$

Where:

A_j = The grand-mean centered school achievement level for school j, coded continuously

M_j = The grand-mean centered percent minority (black, Latino/a or Native American) in school j
S_j = The grand-mean centered saturation of fixed mindset for school j

The significance and direction of δ will answer RQ 4a. The significance and direction of λ will answer RQ 4b. We do not have a substantive hypothesis about the π parameter, for minority composition, but would attempt to interpret and understand it if it was significant.

We will test whether there is a significant reduction in τ^2 as a result of the inclusion of school-level covariates (i.e. comparison of questions (3) and (4)). This will answer the research question of whether these three school-level factors in general explain variability in the GM effect among schools.

Second, we will use the school-achievement level variable coded into the three categories that constituted the sampling strata (bottom 25%, middle 50%, and top 25%) and conduct planned contrasts of subgroup ATEs. In the second model mindset saturation will still be a continuous variable.

Third, with consultation from statisticians, we will evaluate potential non-parametric models to examine the independent and interactive impact of school-level moderators on between-school variability in the treatment impact β_j in equation 4 (e.g. likely a variation on Bayesian Additive Regression Trees). These models will test robustness of results to potential confounds in the school-level moderators (such as rural/urban or poverty concentration). To avoid over-interpretation of results, we will provide the statisticians with a dataset where the name of the variable and the meaning of the value labels are masked. The initial summary of the significant moderators will be generated by the statisticians, blind to the identities of the variables or of the treatment or control values.

19. Transformations

19.1. If you plan on transforming, centering, recoding the data, or will require a coding scheme for categorical variables, please describe that process.

See indices above.

20. Follow-up analyses

20.1. If not specified previously, will you be conducting any confirmatory analyses to follow up on effects in your statistical model, such as subgroup analyses, pairwise or complex contrasts, or follow-up tests from interactions. Remember that any analyses not specified in this research plan must be noted as exploratory.

See primary analyses above and planned analyses below.

We will also examine whether the data meet the assumptions of the linear models. If they do not, we will adjust the model and possibly the estimation methods for standard errors to fit the data as appropriate. We expect the linear models with robust standard errors to be appropriate, however.

For research question 4, we will test the planned sub-groups of “low” “medium” and “high” achieving schools, and we will allow the penalized non-parametric models to tell us where subgroup effects are appearing.

Analyses of the characteristics of schools that did and did not agree to participate will be used to assess whether the answers to the RQs generalize the population of *all* 9th grade regular U.S. public high schools or only those represented by the sample that agreed to participate. We expect that non-participation will be unrelated to observable characteristics following non-response adjustment (i.e. weighting).

21. Inference criteria

21.1. What criteria will you use to make inferences? Please describe the information you will use (e.g. p-values, Bayes factors, specific model fit indices), as well as cut-off criterion, where appropriate. Will you be using one or two tailed tests for each of your analyses? If you are comparing multiple conditions or testing multiple hypotheses, will you account for this?

$p<.05$, two-tailed, for RQ1-3. For RQ1-3 we limit the potential for a multiple testing problem by using one outcome (GPA), measured in a one way, to answer these confirmatory research questions, resulting in three null hypothesis tests.

For RQ4, we reduce multiple testing by using two operationalizations of school achievement (continuous vs. dichotomous) and two operationalizations of mindset saturation (self-report and behavioral). These four null hypothesis tests will use $p <.05$. We will then use Bayesian inference with penalties for over-fitting for the flexible, non-parametric models; because the models are Bayesian, they do not involve null hypothesis tests. .

22. Data exclusion

22.1. How will you determine what data or samples, if any, to exclude from your analyses? How will outliers be handled?

Participants will be included as long as they saw the first page of the treatment or control exercises and had linked student transcript data.

23. Missing data

23.1. How will you deal with incomplete or missing data?

For academic outcomes we will default to listwise deletion for missing data. We will examine whether there was any attrition from the study (i.e. mid-semester dropouts) and whether that was differential by condition. If so, we will consult with statistical experts to develop a missing data plan (e.g. weights or propensity scores).

For prior achievement, we will impute missing grades using test scores or self-reported expectations for doing well that year (when students have data on those variables). Imputed values will be z-scored so that they are on the same metric as the grades. This approach comes in part from Yeager, Romero et al. (2016), who created a prior achievement composite with grades, test scores, and self-reported expectancy for grades. Expectancies were an intervention moderator in a related intervention (Hulleman & Harakeiwicz, 2009).

24. Planned additional analyses (optional)

These four sets of planned exploratory analyses supplement the primary research questions above and will be reported in the manuscript or supplement regardless of the outcomes:

1. **Poor performance rate**. We will report a secondary outcome of poor performance, defined as a D/F average in core courses at the end of 9th grade. We will attempt to replicate the results reported in Yeager, Romero et al. (2016) and Paunesku et al. (2015), which found reductions in poor performance rates for treated individuals (also see Yeager et al. 2014, *JEP:General*). Poor performance rates furthermore represent a conceptual replication of higher-education interventions, which found treatment effects on full-time enrollment rates (Yeager et al., 2016). Although GPA is the primary outcome, the poor performance rate is also highly practically relevant because it is a strong predictor of eventual high school graduation (see Allensworth et al. 2005).

2. **Results for different courses**: We will report results separately by course (math, English, social studies, etc.) either in the paper or in an online supplement. There might be larger effects in math and science, under the assumption that lay beliefs about fixed ability are stronger in math and science and therefore might benefit more from correction via a growth mindset treatment.

3. **Intervention fidelity**: We will assess the implementation fidelity of our treatment and control conditions with the following measures: (1) the percentage of open-ended questions that students answered during their on-line sessions, (2) the percentage of screens that students opened (and presumably viewed) during their on-line sessions, (3) the student-level response rate, (4) the amount of distraction that students reported experiencing during their on-line sessions, and (5) the amount of distraction that students reported other students experienced during their on-line sessions. We will create a composite of all or a subset of these (using factor analysis or analogous data-reduction methods) and aggregate to the student and school level. We will explore whether intervention fidelity explains differences in treatment impact, and whether it is a mechanism for potential moderation by achievement level.

4. **Strength of manipulation check**: We will explore whether different schools show different treatment effects because they were more or less successful at delivering the treatment in a way that caused students to change their attitudes and interim behaviors, as measured by the size of the treatment effect on manipulation checks (self-reported mindsets and challenge-seeking behavior, after receiving the treatment) across schools.

The below additional planned exploratory analyses test alternative research questions. They could be presented as secondary analyses for the primary paper, or they could constitute papers of their own. Although these questions are not fully developed, we pre-register them here so that they are listed prior to seeing or analyzing any results, so as to constrain researcher degrees of freedom.

5. **Student-level moderators**: (1) academically negatively-stereotyped minority students (e.g., black, Latino, native American) vs. academically non-negatively stereotyped students (white or Asian-American students), (2) females vs. males (especially in quantitative classes), (3) and students who are socioeconomically disadvantaged (defined either by parental education/occupation or by free/reduced price lunch status) vs. advantaged students, (4) school track (i.e. advanced math vs. regular math); (5) attitudinal measures obtained at the beginning of students' first on-line session, such as initial growth mindsets (to replicate the marginally-significant moderation in Blackwell et al., 2007), expectations for academic success (conceptually replicating Hulleman & Harackeiwicz, 2009), or math anxiety. In general, we will test the conceptual hypothesis that students who face more disadvantage or have greater vulnerability might also show stronger treatment effects.

6. **Interaction of achievement level and mindset saturation**: When investigating research question 4, achievement level and mindset saturation could potentially interact. The greatest treatment effects might occur in places where there is the highest school-achievement level, but the weakest school level mindset saturation.

7. **Adding convenience sample schools to increase cross-site statistical power**. A planned supplemental analysis for Research Questions 3 and 4 will combine the treatment effect estimates obtained in the pilot study (Yeager et al. 2016) and in a replication in a convenience sample of urban district schools (Hanselman et al. in prep) with the national study estimates, to increase the number of schools by 18. This will increase our power to detect cross-site variation in treatment effects. After merging these schools' data with the national sample, we will re-conduct the analyses for Research Questions 3 and 4.

8. **Timing**: We will explore whether timing during the year (e.g. August vs. January), and timing during the day moderated treatment impacts. We have a working hypothesis that receiving an intervention during a busy time (e.g., right before thanksgiving or a holiday, on a Monday or Friday, the last period of the day) will show weaker effects. We will explore the hypothesis that timing within the year is a predictor of school likelihood of compliance (under the assumption that schools that participated earlier were more willing partners), and so experiments conducted earlier in the year might show larger treatment effects.

9. **School minority composition and stereotype threat**: we will test the exploratory hypothesis that negatively-stereotyped minority students in low-minority high schools might benefit most from the treatment. This would be a conceptual replication of a study of affirmation (a different psychological intervention) by Hanselman et al., 2014 and a potential test of stereotype threat explanations for growth mindset intervention effects.

Script (Optional)

The purpose of a fully commented analysis script is to unambiguously provide the responses to all of the questions raised in the analysis section. This step is not common, but we encourage you to try to create an analysis script, refine it using a modeled dataset, and use it in place of your written analysis plan.

25. Analysis scripts (Optional)

25.1. (Optional) Upload an analysis script with clear comments. This optional step is helpful in order to create a process that is completely transparent and increase the likelihood that your analysis can be replicated. We recommend that you run the code on a simulated dataset in order to check that it will run without errors.

NA

Other

26. Other

26.1. If there is any additional information that you feel needs to be included in your preregistration, please enter it here.

Other analyses are possible with this dataset. For instance, we plan to study whether variance in the treatment impact across math classes varies due to characteristics of teachers and classrooms. We also plan to conduct correlational analyses of the math classroom data. We will strip the present dataset of the teacher identifiers so that we can pre-register those analyses prior to conducting them.

An earlier version of this pre-registration was uploaded but not approved by researchers. It was “frozen” by the OSF robots after researchers did not approve it within 48 hours, but the plan was not complete because it had not yet been reviewed by MDRC. The present version has been reviewed by MDRC and is final and complete. We have “withdrawn” the previous frozen version.
