# Supplementary material for: A national experiment reveals where a growth mindset improves achievement
Source: Nature. 2019 Aug 7;573(7774):364–9. doi: 10.1038/s41586-019-1466-y (PMC6786290; doi:10.1038/s41586-019-1466-y)
Supplement: Supplementary file 2 — Reporting Summary [file 41586_2019_1466_MOESM2_ESM.pdf]

nature research

Corresponding author(s): David S. Yeager, dyeager@utexas.edu, Paul Hanselman, paul.hanselman@uci.edu

Reporting Summary

Nature Research wishes to improve the reproducibility of the work that we publish. This form provides structure for consistency and transparency in reporting. For further information on Nature Research policies, see Authors & Referees and the Editorial Policy Checklist.

Statistical parameters

When statistical analyses are reported, confirm that the following items are present in the relevant location (e.g. figure legend, table legend, main text, or Methods section).

n/a	Confirmed	
☐	☒	The exact sample size (*n*) for each experimental group/condition, given as a discrete number and unit of measurement
☐	☒	An indication of whether measurements were taken from distinct samples or whether the same sample was measured repeatedly
☐	☒	The statistical test(s) used AND whether they are one- or two-sided<br>*Only common tests should be described solely by name; describe more complex techniques in the Methods section.*
☐	☒	A description of all covariates tested
☐	☒	A description of any assumptions or corrections, such as tests of normality and adjustment for multiple comparisons
☐	☒	A full description of the statistics including central tendency (e.g. means) or other basic estimates (e.g. regression coefficient) AND variation (e.g. standard deviation) or associated estimates of uncertainty (e.g. confidence intervals)
☐	☒	For null hypothesis testing, the test statistic (e.g. *F*, *t*, *r*) with confidence intervals, effect sizes, degrees of freedom and *P* value noted<br>*Give P values as exact values whenever suitable.*
☐	☒	For Bayesian analysis, information on the choice of priors and Markov chain Monte Carlo settings
☐	☒	For hierarchical and complex designs, identification of the appropriate level for tests and full reporting of outcomes
☐	☒	Estimates of effect sizes (e.g. Cohen's *d*, Pearson's *r*), indicating how they were calculated
☐	☒	Clearly defined error bars<br>*State explicitly what error bars represent (e.g. SD, SE, CI)*

Our web collection on statistics for biologists may be useful.

Software and code

Policy information about availability of computer code

Data collection	Data were collected via the Qualtrics survey platform.
Data analysis	Data were analyzed in R and Stata. All syntax files are stored on a secure server with the raw data, housed at the University of Texas at Austin Population Research Center.

For manuscripts utilizing custom algorithms or software that are central to the research but not yet described in published literature, software must be made available to editors/reviewers upon request. We strongly encourage code deposition in a community repository (e.g. GitHub). See the Nature Research guidelines for submitting code & software for further information.

Data

Policy information about availability of data

All manuscripts must include a data availability statement. This statement should provide the following information, where applicable:
- Accession codes, unique identifiers, or web links for publicly available datasets
- A list of figures that have associated raw data
- A description of any restrictions on data availability

Data, syntax, and documentation are available to researchers who agree to terms of data use, including analysis on a secure server and prohibitions against any analysis that risks exposing the identity of participating students (i.e., deductive disclosure)

April 2018

Field-specific reporting

Please select the best fit for your research. If you are not sure, read the appropriate sections before making your selection.

☐ Life sciences ☒ Behavioural & social sciences ☐ Ecological, evolutionary & environmental sciences

For a reference copy of the document with all sections, see nature.com/authors/policies/ReportingSummary-flat.pdf

Behavioural & social sciences study design

All studies must disclose on these points even when the disclosure is negative.

Study description	This study involves secondary data analysis of an intervention evaluation conducted on behalf of schools in the U.S. The program evaluation was conducted in partnership with districts that were interested in knowing whether a growth mindset intervention would benefit their students.
Research sample	The sample involves approximately 12,500 students attending 65 U.S. public schools.
Sampling strategy	Schools were selected from a list of over 12,000 Regular U.S. public schools using a stratified random sampling method.
Data collection	A third party research firm collected all data from students in school computer labs and acquired administrative data in partnership with school districts. The firm cleaned and merged all data and delivered de-identified datasets to the UT Austin PRC. A different third party research firm then processed the data to produce an analytic file.
Timing	Data were collected during the 2015-2016 academic year, between August 2015 and June 2016.
Data exclusions	Data were excluded only for non-response, missing data, or ineligibility, as coded by the third-party research firm. Exclusions were carried out in accordance with the pre-registered analysis plan, as noted in the main text. Exclusions are described in full in the supplemental material.
Non-participation	Non-participation (and disclosures relevant to a CONSORT report) appear in the supplemental material.
Randomization	Participating students were randomly assigned at the person level to treatment or control by the survey software.

Reporting for specific materials, systems and methods

Materials & experimental systems

n/a	Involved in the study
☒	☐ Unique biological materials
☒	☐ Antibodies
☒	☐ Eukaryotic cell lines
☒	☐ Palaeontology
☒	☐ Animals and other organisms
☒	☐ Human research participants

Methods

n/a	Involved in the study
☒	☐ ChIP-seq
☒	☐ Flow cytometry
☒	☐ MRI-based neuroimaging
